# Bacterial gene 5′ ends have unusual mutation rates that can mislead tests of selection

**DOI:** 10.1371/journal.pbio.3003569

**Published:** 2025-12-15

**Authors:** Sofia Radrizzani, Juan Rivas-Santisteban, Namshik Han, Laurence D. Hurst

**Affiliations:** 1 Milner Centre for Evolution, Department of Life Sciences, University of Bath, Bath, United Kingdom; 2 Milner Therapeutics Institute, Jeffrey Cheah Biomedical Centre, University of Cambridge, Cambridge, United Kingdom; 3 Systems Biology Department, Centro Nacional de Biotecnología, CSIC, Madrid, Spain; 4 Cambridge Centre for AI in Medicine, Department of Applied Mathematics and Theoretical Physics, University of Cambridge, Cambridge, United Kingdom; 5 Wellcome-MRC Cambridge Stem Cell Institute, Jeffrey Cheah Biomedical Centre, University of Cambridge, Cambridge, United Kingdom; National Centre for Biological Sciences, INDIA

## Abstract

Despite early assumptions of neutrality, numerous mechanisms are now thought to cause selection on synonymous mutations, commonly supported by a low evolutionary rate at synonymous sites (*K*_s_). This has been best evidenced in the first ~10 codons of genes in *E. coli,* where *K*_s_ is less than around half that of the gene body. Diverse lines of evidence support the hypothesis that these first ~10 codons are under selection for high AT content which causes low mRNA stability that in turn enables ribosomal initiation. There remains one enigmatic discrepancy, however, namely that the low *K*_s_ domain extends far beyond the first 10 codons. Here we ask why this is. As we see no evidence that the zone influencing protein levels has been misestimated, we consider three further hypotheses: that reduced *K*_s_ is a) owing to overlapping genes, b) reflects an extended slow translational “ramp,” and c) is mutational. We reject the first two as in both *E. coli* and *Bacillus sp.* the extended low *K*_s_ domain persists on analysis of non-overlapping genes and in *Bacillus*, where fast optimal codons tend to be A/T-ending, a fast-to-slow codon trend is seen. We fail to falsify the third hypothesis. Employing mutation accumulation data for *E. coli* we show that the 5′ end has a lower mutation rate, with the first 10 codons having a rate around half that of the gene body, this then steadily increasing following the trend seen for *K*_s_. Compositional variation is likely to explain some of the difference, the 5′ end lacking GC-rich runs while these are most mutagenic. We conclude that even a highly reduced *K*_s_ is not always adequate to substantiate selection on synonymous mutations. This result has broad implications for inference of the causes of evolutionary rate variation.

## Abstract 2

A reduced rate of synonymous site evolution is often presented as key evidence for selection on synonymous mutations. However, this study shows that for the paradigmatic example in bacteria, where the rate is especially low in the first 10 codons of genes, this is well explained by mutation, not selection.

## Introduction

Owing to the degeneracy of the genetic code, many coding sequence (CDS) mutations change the identity of a codon but not the encoded amino acid. As they leave the protein sequence unchanged, such synonymous mutations were commonly assumed to be evolutionarily neutral [1,2], hence also originally being termed “silent” mutations. However, subsequently, over-usage, especially in highly expressed genes (HEGs), of the synonymous codons that match the more abundant tRNAs was observed in many organisms, leading both to the concept of the translationally “optimal” codon and to the understanding that synonymous mutations can be under selection [3–6]. It remains a matter of debate as to whether selection on such optimal codons is mediated by selection on ribosomal speed or ribosomal accuracy (see, e.g., [7–11]). Since then, there has been evidence for many further mechanisms by which synonymous mutations, and in turn codon choice, influences gene expression or more generally can be under selection (see reviews, e.g., [12–16] and references therein). A non-exhaustive list of these mechanisms includes modulation of splicing [17–19], destruction of functionally relevant miRNA pairing sites [20] or their creation [21], modulation of RNA stability and structure [22–25], and modulation of protein folding via alteration of ribosomal velocity (see reviews, e.g., [26,27]). More generally, RNA or DNA binding by proteins or RNAs can be affected by synonymous mutations, including intra mRNA self-pairing affecting mRNA stability [22,25,28,29]. Most of these mechanisms operate locally within CDSs, potentially causing localized sequence conservation [30,31], but selection for “optimal” codons can apply across the bulk of a gene’s CDS. Understanding these mechanisms is important not just for understanding the modes of selection operating on gene sequences, above and beyond classical selection on the protein product, but also for improved diagnostics, disease etiology determination, and transgene (heterologous gene) design.

Classically, a central piece of evidence that particular genes, or gene regions, are subject to stronger selection on synonymous mutations is a low synonymous rate of evolution (*K*_s_), this being expected when synonymous mutations are removed from populations by purifying selection. For example, lower *K*_s_ in genes with higher codon usage bias [32], in alternatively spliced exons [31], in exonic splice enhancer motifs [18,33], or in miRNA binding sites [20,34] has been employed to infer the action of such purifying selection. Similarly, the degree of conservation at any given synonymous site is employed in tools to infer the likely pathogenicity of synonymous mutations [35–37], and methods that don’t employ conservation as an input variable report that predictions nonetheless correlate with conservation [38].

Here, we revisit the best-evidenced incidence of selection on synonymous mutations and in the process question whether a reduced *K*_s_ is adequate to substantiate selection on synonymous mutations. Our exemplar concerns the 5′ CDS end of genes in *Escherichia coli* (*E. coli*). Here, molecular evolutionary analysis, *in silico* analysis of native genes, and, importantly, many experimental manipulations of native and transgenes all reinforce the same narrative. It was first observed that the first 10 or so codons of *E. coli* native genes have a distinct nucleotide content compared to those more downstream in the CDS, as they are characterized by high A/T content, especially A, and low G/C content, especially G [39]. The same analysis identified a much lower synonymous substitution rate (*K*_s_) in the 5′ end, consistent with functionality and purifying selection on synonymous mutations to maintain the high AT content [39]. We estimate that the minimum *K*_s_, seen at codon 2, is less than a quarter of that seen in the gene body, with the average *K*_s_ across the first 10 codons about a half that of the gene body (see below). In turn, it was suggested that this high A/low G content may reflect selection for reduced mRNA stability in this domain [39], a trend now considered to be phylogenetically universal [40].

This low stability effect and the influence of the first ~10 codons have been repeatedly confirmed in *E. coli* large-scale transgene experiments [41–47]. While the degree to which the transcript as a whole employs translationally optimal codons is either not a significant predictor of protein level or a very weak one at most [41,43], the stability of the 5′ region is highly influential. For example, in comparing over 100 GFP transgenes varying exclusively at synonymous sites, that differed by orders of magnitude in their protein output, RNA stability of the region −4 bp to +37 bp around the start codon (i.e., the first 12 or so codons in the CDS) was found to be highly correlated with protein abundance [43]. Similarly, Goodman and colleagues [42] showed that when the synonymous codon usage of the first 10 codons was altered, the predicted RNA stability in this domain, across 14,000 transgenes, strongly predicted protein level. This was confirmed in an even larger scale analysis of 244,000 transgenes where the 5′ stability spanning the first 10 codons was seen to be the strongest predictor of protein abundance by some magnitude [41]. All such studies indicate that the first 8–12 codons are influential in determining protein levels [41–47], but for brevity we refer to the first ~10 codons. In native mRNAs, the local folding energy in the first ~30 CDS nucleotides is weaker than downstream [48,49], as would then be predicted.

While there seems little doubt that low stability is influential, the exact mechanistic logic of the low stability effects remains debated with multiple mutually compatible reasons as to why low 5′ mRNA stability might promote translation: it is less energetically demanding for the ribosome’s helicase activity [50]; strong folds may obscure the Shine-Dalgarno sequence [51]; RNA folding mediates susceptibility to degradation [52] or it modulates ribosomal velocity and efficient queuing [53].

Recent evidence provides additional support to the model that the high AT content at 5′ ends is owing to the activity of selection favoring A and T. Notably, from mutation accumulation (MA) experiments, Long and colleagues [54] determined *E. coli*’s neutral-mutation bias equilibrium [54], deviations from which imply the action of some process other than drift [54]. Consistent with selection favoring AT at the 5′ end, they find neutral equilibrium AT content to be 0.52 for *E. coli* [54]*,* which is lower than the observed at synonymous sites in native genes which in the 5′ CDS reaches up to 0.62 at codon third sites (see [39] and our analysis below). This is consistent with selection favoring G/C->A/T mutations. More recently, average nucleotide diversity among native *E. coli* genes at 4-fold synonymous sites was also shown to be low at 5′ ends, also in accordance with strong purifying selection [63].

While the above evidence provides what appears to be an unusually complete and robust understanding of selection favoring A/T at synonymous sites at 5′ ends, there remains at least one unexplained discrepancy: while both mononucleotide usage trends and transgene studies point to the first ~10 codons as being influential, early studies showed that synonymous site conservation trends in *E. coli* don’t follow this “10-codon” rule [39]. It is true that rates of synonymous substitutions start exceptionally low in proximity of the initiating 5′ start codon [39]. However, they plateau further downstream around codon 30 [39] (in our analysis below, we see until codon 60). Similarly, the nucleotide diversity among native *E. coli* genes at 4-fold synonymous sites increases within approximately the first ~60 codons, after which it stabilizes [63]. Understanding the causes of this anomaly is of importance in transgene design as it raises the question of whether modifications further downstream than codon 10 can further optimize protein production. It also questions whether the low *K*_s_ values are necessarily indicative of selection on synonymous mutations or at least in the manner proposed.

Under the philosophy that one should treasure one’s exceptions [64] (or at least anomalies), here we consider a series of possible explanations for the extended low *K*_s_ domain. One possible explanation is that we have misunderstood the size of the 5′ domain that affects protein titer (meaning either absolute protein levels or protein per mRNA molecule). To address this, we first examine why the first ~10 codons are considered distinct. These analyses reinforce the centrality of the first ~10 codons not least on its influence on protein levels.

As we see no clear evidence that the functionally distinct domain extends far beyond the first ~10 codons, we consider three further hypotheses to explain the much longer domain of reduced *K*_s_: that it reflects the impact of gene-gene overlaps (where the 5′ end of any given CDS overlaps with the start or end of a neighboring CDS); that it reflects selection for an extended “ramp” of slow translating codons (explained more fully below); that it is mutational in origin.

The overlap model is intrinsically attractive. As gene-gene overlaps are likely to be more common in proximity to the start codon than further into the gene body, they could predict a gradually rising *K*_s_, especially if the overlaps involve non-synonymous sites in one gene being synonymous in the other (and vice versa). The effect may be of significance as one third of bacterial annotated genes are overlapping [65] and genes containing overlaps are more conserved [66]. To examine this, we derive the trend in *K*_s_ as a function of distance from the start codon in both *E. coli* and *Bacillus sp.* and ask whether the extended 5′ domain remains when overlapping genes are excluded. We find that not only is the extended *K*_s_ domain also seen in *Bacillus,* but that in neither species do overlapping genes explain the extended *K*_s_ domain.

The discovery that the 60-codon low *K*_s_ domain is also seen in *Bacillus* permits a novel means to interrogate the second hypothesis, namely that the extended *K*_s_ domain accords with a hypothesized translational “ramp” that, given trends in codon usage, is conjectured to extend up to around codon 50–60 [53,67,68]. Given that codon adaptation is reduced towards the 5′ end of genes in *E. coli* (i.e., there is over-use of non-optimal codons), Tuller and colleagues hypothesized that this was an adaptation to slow ribosomes to enable a more orderly initiation process (a “ramp” [67]). The ramp model was extended to include positive charge on the N-terminal peptide, this hypothesized to slow ribosomes owing to an interaction with the negatively charged exit tunnel [53].

While potentially attractive in proposing effects running into the gene body, we consider the ramp model to be not especially likely, as it has met with considerable challenges. The original central evidence, namely that ribosome protection assays report higher ribosome density towards the 5′ end, was found to be both an artifact of higher initiation rates of shorter peptides [69] and of sample preparation (and not replicated when the method of ribosome stalling was adjusted [70]). The positive charge effect can be accounted for as an epiphenomenon of the fact that membrane proteins orientate so that excess positive charge near hydrophobic membrane-spanning regions is on the cytoplasmic side of the membrane, the positive-inside rule [71]. Importantly in the current context, any trend for non-optimal codon usage is argued to be a necessary correlate of CDS A/T richness, predominantly associated with RNA stability effects [72], *E. coli*’s optimal codons being mostly G/C-ending. Consistent with this, in pairwise consideration of constructs of different codon optimality controlling for RNA stability, there are no deterministic effects on transgene output [72]. In large-scale transgene data, protein output is independent of 5′ codon non-optimality when allowing for RNA stability [42]. Osterman and colleagues also altered tRNA availability and saw no effect of the influence of the relevant codons to transgene output [47]. We recently showed in multiway partial correlation analysis of 244,000 transgenes that, if anything, higher codon adaptation in the first 10 codons is predictive of higher protein production, although the effect is extremely weak [73].

It was further noticed that the trend to prefer non-optimal codons in the first 10 codons applied exclusively to cases where the optimal codon is G/C-ending [72]. When the optimal codon is A/T-ending, it is enriched at 5′ ends, indicating A/T presence, not codon non-optimality, to be the key force. In this same context, *Bacillus* provides an exceptional test case as its optimal codons are most commonly A/T-ending. Wei and colleagues [74] determine from transcriptional levels of tRNAs (as opposed to tRNA copy numbers [75]), paired with enrichment patterns in HEGs, the unambiguously translationally optimal codon for any given codon block, i.e., set of synonymous codons (see their Table 1 [74]). For 17 blocks that had agreement between measures in *E. coli*, only 4 optimal codons are A/T-ending [74]. A 5′ A preference thus leads to on average over-use of rare/non-optimal codons, as noticed [72]. By contrast, in *Bacillus,* of 14 resolvable blocks, only 4 optimal codons are G/C-ending [74]. Thus, if patterns of codon preference/avoidance are driven by ribosomal slowing, *Bacillus* should have G/C-rich third sites at 5′ ends to provide low usage of optimal codons, while if codon optimality is irrelevant and A/T content (potentially for low RNA stability) matters, then it should have high codon optimality at the 5′ end and hence have the very opposite of a ramp: fast codons should give way to slower ones. We see over-use of “fast” optimal codons running until about codon 10–15. After this, there is no change in codon usage despite the low *K*_s_ persisting to around codon 60. Comparing between synonymous codons, third site nucleotide content robustly predicts the observed patterns, with A/T-ending codons preferred at CDS ends, G/C ones avoided, regardless of optimality. The *Bacillus* data thus contradicts all expectations of the ramp model. Neither model accounts for the dimension of the *K*_s_ domain (up to codon 60) in either species.

**Table 1:** Estimated genomic AT content at mutational equilibrium determined by simple method across different species and data sets.

Species name	RefSeq accession	Data set	Experiment	A/T >G/C	G/C >A/T	A/T >A/T	G/C >G/C	Sum non-neutral	Sum all	AT*	AT	Codons 2–11			Codons 12-60			Codons 60+			Intergenic
												AT	AT3	AT3 max	AT	AT3	AT3 max	AT	AT3	AT3 max	AT
***E. coli***	GCF_000005845.2	Wei and colleagues (2022) [56]	MA	470	590	125	239	1,060	1,424	0.55 ± 0.016	0.49	0.56	0.55	0.62	0.48	0.45	0.47	0.48	0.44	0.46	0.58
***E. coli***	GCF_000019385.1	Zhang and colleagues (2018) (high depth) [57]	DS	3327	21,063	109	700	24,390	25,199	0.86 ± 0.012	0.49	0.56	0.55	0.62	0.48	0.45	0.47	0.48	0.44	0.46	0.58
***E. coli***	GCF_000019385.1	Zhang and colleagues (2018) (low depth) [57]	DS	21,444	44083	9680	19,077	65,527	94,284	0.67 ± 0.014	0.49	0.56	0.55	0.62	0.48	0.45	0.47	0.48	0.44	0.46	0.58
***E. coli***	GCF_000005845.2	Foster and colleagues (2015) (PFM2, ED1a, IAI1) [58]	MA	326	618	61	69	944	1,074	0.65 ± 0.015	0.49	0.56	0.55	0.62	0.48	0.45	0.47	0.48	0.44	0.46	0.58
***E. coli***	GCF_013166975.1	Bhawsinghka and colleagues (2023) [59]	DS	21	176	17	103	197	317	0.89 ± 0.012	0.49	0.56	0.55	0.62	0.48	0.45	0.47	0.48	0.44	0.46	0.58
***M. smegmatis***	GCF_000283295.1	Castañeda-Garcia and colleagues (2020) [60] Kucukyildirim and colleagues (2016) [61]	MA	336	515	16	50	851	917	0.43 ± 0.016	0.33	0.36	0.25	0.32	0.32	0.15	0.19	0.32	0.14	0.14	0.39
***B. subtilis***	GCF_000186085.1	Sung and colleagues (2015) [62]	MA	159	155	28	8	314	350	0.56 ± 0.016	0.56	0.63	0.64	0.69	0.56	0.57	0.59	0.56	0.56	0.57	0.64

Method to derive equilibrium AT (“AT*”) involves consideration of proportion of mutations directed G/C->A/T among the sum of non-neutral mutations (i.e., those directed G/C->A/T and A/T->G/C) normalized by genomic nucleotide content, see Methods. The error range included represents the standard deviation of the mutational equilibria calculation for 95% bootstrap bounds of 1,000 re-samplings. See column “data set” for respective references for data retrieval. Column “experiment” indicates the approach by either mutation accumulation (MA) experiments, or spontaneous mutation data with Duplex Sequencing. “AT” is the observed genomic AT content for each genome accession. We then include average AT content, average AT content at codon third sites (AT3), and maximum AT3 (AT3 max) in three gene regions: codons 2–11 (first 10 codons after the start codon), codons 12–60, and codons 60+. Finally, we include average AT content of intergenic regions only. All the AT content measures for specific regions for *E. coli* are for accession GCF_000005845.2, for *M. smegmatis* for accession GCF_000283295.1, and for *B. subtilis* for accession GCF_000186085.1. The data underlying this table can be found in https://doi.org/10.5281/zenodo.17378284.

Our final hypothesis is that the reduced *K*_s_ reflects differences in the mutation rate. We test this using MA data in *E. coli* [56]. From this, we discover that indeed, the first 60 codons have an unusually low mutation rate, with the first 10 codons having a rate around a half that of the gene body, the rate then gradually rising. This proportional difference is comparable to that seen for the synonymous rate of evolution. We replicate this result using independent data sets. In part, this effect is explained in *E. coli* by skewed nucleotide content at the 5′ end as this is AT-rich and the more mutable nucleotide runs are GC-rich. Taken together the analyses question inferences drawn from classical analysis of reduced synonymous rates of evolution.

## Results

### The first ~10 codons are unusual in both nucleotide content and in their influence on protein levels

Before asking about possible alternative explanations, we start by asking whether there is evidence that the 5′ zone that may be considered distinct may have been misestimated. We consider trends in nucleotide usage, in RNA stability, in influence on protein expression, and on cellular growth of 5′ CDS codons.

#### The first 10 codons of native genes show distinct nucleotide content trends compared to downstream codons.

**Fig 1:**
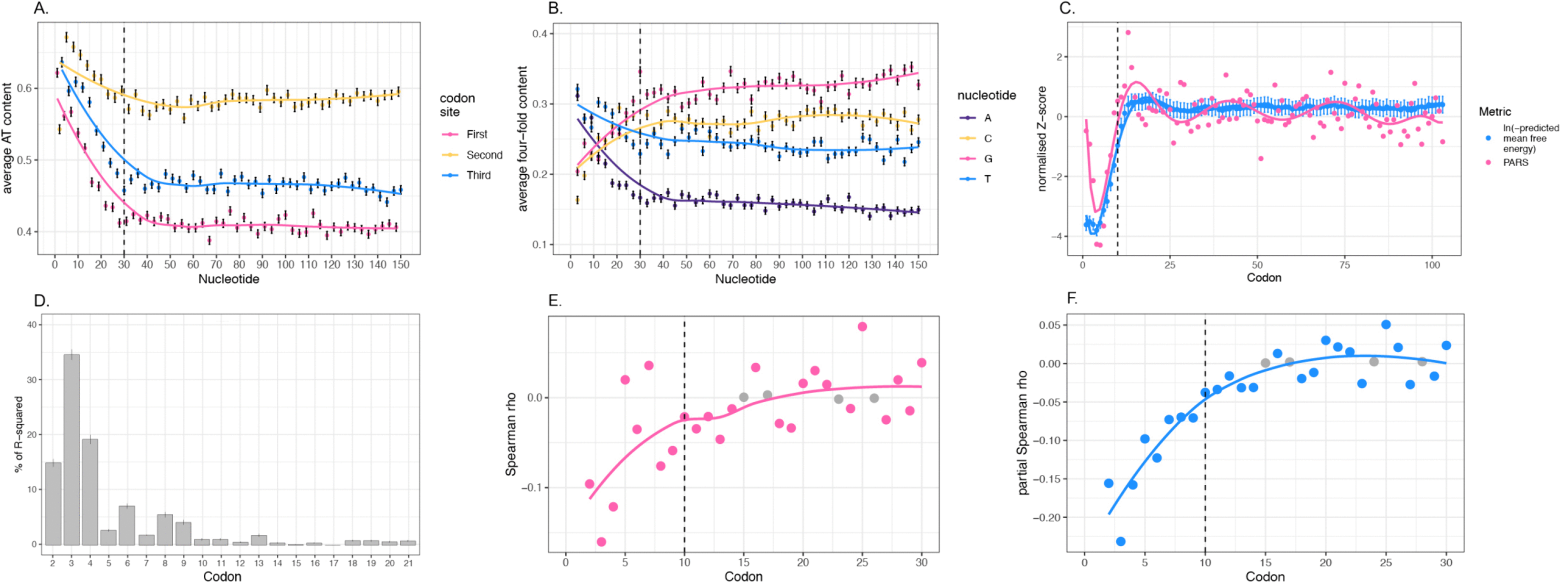
Examination of the uniqueness of the first ~10 codons in *Escherichia coli* CDSs. **A.** AT content by nucleotide position, divided into the three codon sites. **B.** Content for each of the four nucleotides by position at third sites of 4-fold degenerate codons. In both A and B, content is averaged at each nucleotide position across 5,098 native genes. The *x* axis represents nucleotide positions relative to the start codon (i.e., the first nucleotide of the codon after the start codon is at position 1). Error bars indicate the standard error of the mean (SEM). Dashed vertical black line marks the first 10 codons. Locally estimated scatterplot smoothing (LOESS) regression lines are included. **C.** Average mRNA stability by codon position. Stability was determined by two measures. First, in blue, it was computationally predicted using ViennaRNA R package [55] through a sliding window approach scanning the CDS of native genes (starting at nucleotide position −15). The *x* axis represents the position at the middle of each 30 bp window. Second, experimental PARS data was employed (in pink), data from Del Campo and colleagues [49]. Dashed vertical black line marks the first 10 codons. A polynomial regression of order 10 is shown for both. For ease of comparison, in both cases Z normalized data is employed with a low (negative) figure indicating lower stability. **D.** Relative influence of GC content at each codon position on protein per RNA levels (protein/RNA) in transgene constructs. Relaimpo analysis was conducted using the lmg model with 1,000 bootstraps, 90% bootstrap confidence intervals are shown as error bars. Codon positions on the *x* axis refer to absolute numbers (where start codon is 1). Transgene data from Cambray and colleagues [41]. **E.** Full Spearman correlations between GC content and protein per RNA (protein/RNA) levels in transgenes for each codon position. **F.** Partial Spearman correlations controlling for the influence of all other codon positions in the available construct sequence (codons 2–30) such that, for instance, the relation between transgene expression (as protein/RNA) and GC content at codon 2 is found independently of the relation with GC content in codons 3–30. In E, F, colored points represent rho values with a *P*-value ≤ 0.05, while gray points are non-significant. Locally estimated scatterplot smoothing (LOESS) regression lines are included. Codon positions on the *x* axis refer to absolute codon numbers (e.g., the start codon is codon 1). Dashed vertical black line marks the first 10 codons. Transgene data from Cambray and colleagues [41]. Plots D–F were repeated using Cambray protein levels only, not normalized by RNA (see S2 Fig). The data underlying this Figure can be found in htps://doi.org/10.5281/zenodo.17378284.

For completeness and the convenience of the reader, we start by illustrating AT content variation across the 5′ regions of 5,098 native *E. coli* genes, similar to that done previously (e.g., [39]). At all codon positions there is low GC content in the first few codons, which gradually increases up to codon 10 (Fig 1A). Beyond this region, AT content stabilizes and remains relatively uniform across nucleotide positions (Fig 1A). We note that this pattern holds true across all three codon sites, although in the 5′ domain the second site is the most AT-rich, an observation we return to later (see Discussion and S1 Text) as this is indicative of protein-level effects [76] (see S1 Fig for content by codon position relative to the start codon, by position within codons, i.e., sites 1, 2, and 3, for all four nucleotides individually).

We also illustrate the nucleotide trends only at synonymous sites in the strictest sense by finding average content at third sites of 4-fold degenerate codons by position (Fig 1B). There is a difference in 5′ nucleotide content when compared to core in all four nucleotides, with 5′ ends characterized by high A/T and low G/C, all plateauing around codon 10. Codon usage at codon positions 2–10 is significantly different to downstream, with an over-representation of A-ending codons independent of the amino acid used [77]. As reported (e.g., [39]), we also find that the bigger differences are in A and G content, compared to T and C (Fig 1B).

#### The first 10 codons of native genes show lower mRNA stability compared to downstream codons.

Given that the nucleotide content is different at 5′ ends and in gene cores, we seek to confirm the previously reported explanation thought to underpin this: mRNA stability trends within the CDS. Through computational prediction of mRNA stability using a 30 bp sliding window approach, we find the predicted stability by codon position. We observe that predicted stability is lowest at 5′ ends and gradually increases until plateauing shortly after codon 10 (Fig 1C), consistent with previous mRNA stability and secondary structure studies, both computational [43,67] and experimental [49], as well as nucleotide content trends (Fig 1A and 1B). We note that minimum of low stability is around codon 3, in agreement with experimental mRNA stability data [49] (Fig 1C).

#### The first 10 codons are most influential on transgene expression.

The above analyses highlight (and confirm) the distinct nature of the first ~10 codons. We next look at the influence of 5′ codons as regards transgene protein production. For this analysis, we employ data from the large-scale study by Cambray and colleagues [41], which includes 244,000 transgenes expressed in *E. coli* with randomized codon usage in the 5′ CDS up to codon 33. Given the variability in expression levels associated with synonymous codon usage, we investigate which codon positions have the greatest influence on the protein per mRNA (protein/RNA) measures they report.

Using a Relative Importance of Regressors in Linear Models (relaimpo) analysis [78], we assess the influence of GC content at each codon position while accounting for the effects of other positions. Although Cambray and colleagues [41] provide sequences for each transgene of 33 codons, as a relaimpo analysis becomes exponentially more computationally intensive for each codon position added, we consider the first 20 codons following the start codon (with 21 variables, relaimpo analysis considers in excess of 10^19^ different models, with 30 variables it goes beyond most computing power at 10^32^). We nonetheless opt to employ relaimpo as it comes with the advantage that it allows us to evaluate the explanatory contribution of each predictor, while considering covariances between each. Results indicate that codons 2–9 exhibit the highest relative influence on expression, with codon 3 showing the greatest impact (Fig 1D). The centrality of codons 2–4 broadly supports experimental analyses [44,79].

To further investigate the positional effects observed in the relaimpo analysis, particularly those beyond codon 21, and to determine directionality of effects, we consider Spearman correlations between protein/RNA levels and GC content for each codon position (S1 Table). We consider both full (Fig 1E) and partial (Fig 1F) Spearman correlations, with the latter controlling for the effects of other codon positions. As expected, correlations were significantly negative for codons 2–14, consistent with the 5′ AT preference in native bacterial genes (Fig 1A and 1B). Notably, correlations plateau near zero around codon 15 (Fig 1E and 1F). For both modes of analysis, comparable results are observed employing protein level (unnormalized to mRNA levels) (S2 Fig).

We then ask whether the correlation between observed nucleotide content and expression (protein per RNA) is also translated into mRNA stability. We thus split the sequence provided by Cambray and colleagues [41] for each transgene and find the correlation between predicted mRNA stability of each sequence subset and transgene expression. We find the computationally predicted stability within the first 10 codons to be significantly more positively correlated to expression when compared to predicted stability of the two sequence subsets (of the same size) downstream (S2 Table).

The transgene analysis comes with the caveat that we are considering influence on protein per RNA (or protein level), while the fitness costs of protein manufacture may be more relevant. However, replacing protein measures with effects on cellular growth rates, we find that GC content—the variable most obviously changing across the 5′ domain—has almost no predictive power, the GC content of codons 2–33 explaining ~2%–3% of variation in fitness (adjusted *r*^2^ = 0.028). In replacing codon GC content with Codon Adaptation Index (CAI), the effect is weaker still (adjusted *r*^2^ = 0.018). Spearman and Spearman partial correlations suggest no obvious significant trend as regards influence on fitness (S3 Fig).

From the above, we surmise that there is no good evidence that the functionally important domain extends far beyond the first 10 codons (for consideration, and rejection, of the ramp model see below). The mystery thus remains as to why conservation at synonymous sites extends far beyond the first 10 codons [39]. We now consider our three further hypotheses, starting with the gene-gene overlap hypothesis.

### Extended synonymous site conservation is not explained by gene–gene overlaps

Prior to consideration of the effects of gene overlap, we first seek to describe *K*_s_ trends using close comparators across all genes (not available to prior analyses [39]). We performed ortholog alignments between three closely related species that provide a “sweet spot” of *K*_s_ estimation (one where the amount of change is low enough that neither alignment nor saturation are an issue, but where there is enough change to be informative), these being *E. coli, Escherichia fergusonii (E. fergusonii)*, and *Salmonella enterica (S. enterica)*. We reconstructed via maximum likelihood the ancestral sequence of the *E. coli* and *E. fergusonii* common ancestor with *S. enterica* as outgroup species. To determine conservation by codon position (relative to the start codon), we separately extract each codon for each ortholog and concatenate it to the codons at that same position for all other orthologs. We do this for the first 150 codons following the start codon to give 150 codon-specific alignments, each containing sequences of at least 1,400 codons (one per ortholog that is at least 180 codons in length so as to avoid capturing 3′ effects). We exclude (rare) cases where all three species have different codons, as it is likely for there to be uncertainty in the ancestral sequence reconstruction at those positions. For each codon position, we then find the synonymous substitution rate (*K*_s_) between the focal species and the ingroup common ancestor from the by-codon position alignment. We can also compute non-synonymous substitution rates (*K*_a_), and the ratio between the two (*K*_a_/*K*_s_), the latter permitting us to address the question of the extent to which the low *K*_s_ might distort measures of protein-level selection.

**Fig 2:**
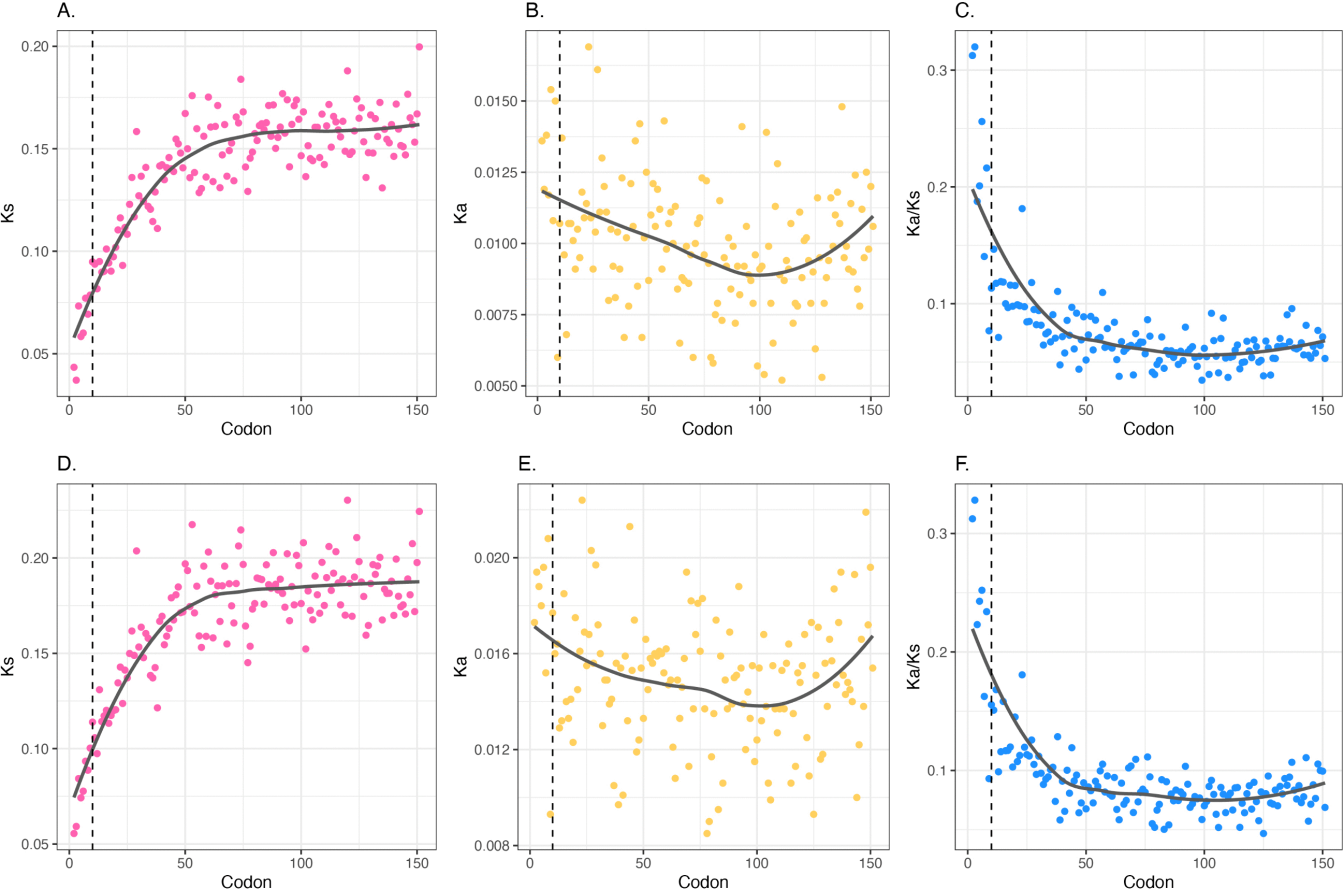
Substitution rates by 5′ codon position comparing the *Escherichia coli– Escherichia fergusonii* ancestor to *E. coli.* **A.** Synonymous substitution rates (*K*_s_); **B.** non-synonymous substitution rates (*K*_**a**_), and **C.** the ratio between the two (*K*_a_/*K*_s_). A–C plots include orthologs that are at least 180 codons in length (*n* = 1,443). **D–F.** same as A-C but only including non-overlapping orthologous genes that are at least 180 codons in length (*n* = 1,310). For all panels, the *x* axis represents absolute codon position (i.e., the start codon is codon 1). Dashed vertical black line marks the first 10 codons. Locally estimated scatterplot smoothing (LOESS) regression lines are included. Note that codon position here is by reference to the codon position in the alignment. Removal of alignment indels in the focal lineage prior to codon position categorization makes no meaningful difference (Pearson correlation between *K*_s_ with indels v *K*_s_ without = 0.99 *P*-value = 1.31 × 10^−142^, likewise for *K*_4_: S8 Fig). The data underlying this Figure can be found in https://doi.org/10.5281/zenodo.17378284.

We find synonymous substitution rates (*K*_s_) to be lowest at the most 5′ codons and increase until approximately codon 60, after which they asymptote (Fig 2A). This is slightly more downstream than the plateauing around codon 30 that was previously reported from highly diverged *E. coli*–*Salmonella* sequence [39]. *K*_a_ rates do not show as clear of a trend (Fig 2B), and *K*_a_/*K*_s_ ratios appear to be primarily driven by *K*_s_ rather than *K*_a_ levels (Fig 2C), which if anything are actually higher in the first few codons than the ones slightly 3′ (Fig 2B). While the most 5′ codons do have the lowest *K*_s_ values, there is no evident change in regression slope around or immediately after codon 10 (Fig 2A). Similar substitution rate patterns were observed when comparing the ancestor to *E. fergusonii* (S4 Fig). Trends for 4-fold degenerate codons (S5 Fig) are similar to those resulting from consideration of all codons (Fig 2). We also see high similarity in trends between operonic and non-operonic genes (S6 Fig). For the set of genes that have an ortholog, the 5′ GC trends are the same as the broader native gene set (S7 Fig).

With this benchmark, next we consider the influence of overlapping CDSs. To account for this, we consider reference genome annotations and repeat the conservation analysis for *E. coli* orthologous genes only considering those that are not overlapping (meaning no other CDS is fully or partially encoded by the 5′ sequence of the focal CDS). We perform the same conservation analysis as above only for non-overlapping orthologous genes (2,027 of 2,385 orthologs) and find that, although absolute *K*_s_ values are marginally higher, the *K*_s_ trend is unaffected (Fig 2D–2F, as above we plot those that are at least 180 codons in length). This is consistent with the hypothesis that conservation at synonymous sites is not fully explained by gene overlaps. In no small part, this reflects the fact that among native genes the majority are not overlapping (669 of 4494 genes show 5′ overlaps, around 15%), and of those that are, most involve small 5′ overlaps (median overlap size = 3 bp, see S9 Fig).

While the above 15% figure is approximately as expected if 30% of genes show an overlap [65] (the others being 3′ overlaps), to determine the generality of results we ask whether the results are replicated in other species. We identified one set of *Bacillus* species with appropriate in and outgroup distances (*B. toyonensis* (focal ingroup) and *B. anthracis*, with *B. mycoides* as outgroup). This also allows us to test whether we observe the same trends in gram-positive bacteria, which differ from gram-negative bacteria (such as *E. coli*) in a number of ways, including lack of co-transcriptional translation in *Bacillus sp.* [80] and a previously reported different codon usage (specifically for *Bacillus subtilis (B. subtilis)* when compared to *E. coli*) [81].

**Fig 3:**
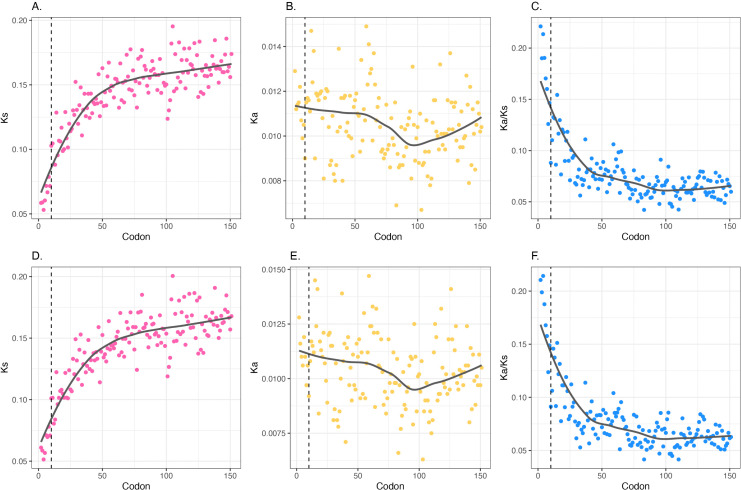
Substitution rates by 5′ codon position comparing the *Bacillus toyonensis–Bacillus anthracis* ancestor to *B. toyonensis.* **A.** Synonymous substitution rates (*K*_s_); **B.** non-synonymous substitution rates (*K*_a_), and **C.** the ratio between the two (*K*_a_/*K*_s_). A–C plots include orthologs that are at least 180 codons in length (*n* = 1,933). **D–F.** Same as A–C but only including non-overlapping orthologs that are at least 180 codons in length (*n* = 1,703). For all panels, the *x* axis represents absolute codon position (i.e., the start codon is codon 1). Dashed vertical black line marks the first 10 codons. Locally estimated scatterplot smoothing (LOESS) regression lines are included. Note that codon position here is by reference to the codon position in the alignment. Removal of alignment indels in the focal lineage prior to codon position categorization makes no meaningful difference (Pearson correlation between *K*_s_ with indels v *K*_s_ without = 1 *P*-value = 2.08 × 10^−156^, while for *K*_4_ = 0.99 *P*-value 8.81 × 10^−146^: S8 Fig). The data underlying this Figure can be found in https://doi.org/10.5281/zenodo.17378284.

Among the *Bacillus* species, we observe the same trends in all genes and in non-overlapping orthologous genes (2,970 of 3,305 orthologs): *K*_s_ starting low 5′ and gradually increasing until plateauing around codon 60 (Fig 3A–3F). For the distribution of overlaps at 5′ ends in native *B. toyonensis* genes see S10 Fig: as for *E. coli*, most genes are not overlapping (479 of 5229 genes show 5′ overlaps, around 9%) and overlaps are commonly short (median overlap size: 3 bp). *B. toyonensis* also replicates similar native GC content along the CDS as in *E. coli*, i.e., plateauing around codon 10 (S11 Fig), suggesting the *K*_s_ trends cannot be explained by different mononucleotide trends in orthologous genes. We further confirm that the extended conservation trend cannot be explained by gene overlaps and furthermore that it is not unique to *E. coli* (or gram-negative bacteria).

### Extended synonymous site conservation cannot be explained by a translational ramp

Having established that in *Bacillus* and *Escherichia* there is both an extended window of low *K*_s_ and that this cannot obviously be explained by overlapping genes, we sought to consider a further prominent model. Given that translationally optimal codon usage is reduced towards the 5′ end of genes in *E. coli*, Tuller and colleagues hypothesized that this was an adaptation to slow ribosomes to enable a more orderly initiation process, what they call a ramp [67]. To investigate this, we consider the trends in usage of unambiguously translationally optimal codons, these matching the most abundant tRNA in the transcriptome and showing classical HEG enrichment patterns.

**Fig 4:**
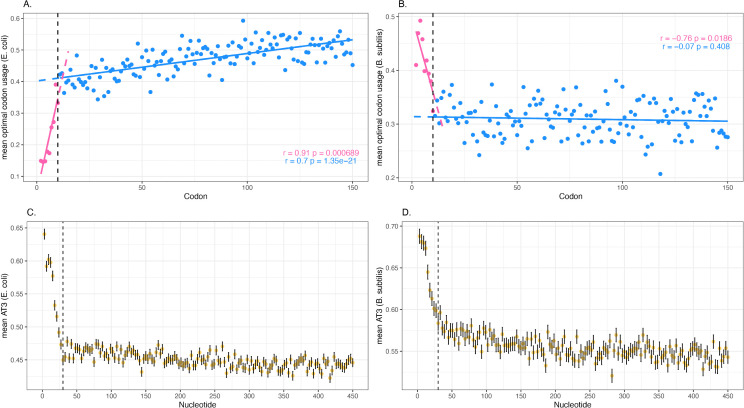
Deviation in usage of optimal codon trends as a function of distance from the CDS start for A. *Escherichia coli*, B. *Bacillus subtilis.* Linear regression and Pearson correlation with respective *P*-value displayed. Lines, points, and statistics in pink consider the first 10 codons (inclusive), those in blue are for all other codon positions. **C.** Trends in AT content at codon third sites across all native *E. coli* genes. **D.** Same as C, but for *B. subtilis*. Note that the *x* axes in all four panels match in terms of position within the CDS as codon position refers to absolute number (where the start codon is codon 1); nucleotide position 1 refers to that following the start codon. Dashed vertical black line marks the first 10 codons. The data underlying this Figure can be found in https://doi.org/10.5281/zenodo.17378284.

We employed the optimal codon calls of Wei and colleagues [74] (see Methods). Plotting the usage of optimal codons against position we see, as previously seen, a tendency in *E. coli* to relatively underuse optimal codons in the 5′ end, this starting to plateau after about codons 10–20 (Fig 4A). It continues up to and past codon 150. In *Bacillus,* optimal codons tend to be A/T-ending so provide a highly informative test case. Indeed, the ramp model still predicts avoidance of optimal codons, while the low stability-A/T epiphenomenon model predicts the opposite of the slow-to-fast ramp. As predicted by the low stability-high A/T model, codon adaptation is extremely high in the 5′ end of *Bacillus* (Fig 4B). After codon 10–20, the trend is flat to negative. Both trends are the opposite of the predictions of the ramp model. As regards optimal codon usage and GC3, in both *Bacillus* and *E. coli*, the trends in operonic genes are the same as those in non-operonic genes (S12 Fig) indicative of effects beyond the ribosome engaging the most 5′ end of the polycistronic RNA.

The centrality of nucleotide content above codon optimality is underscored by analysis of further species in which codon optimality can be defined by reference to tRNA levels [74]. There are four other species for which we have a good definition of optimal codons that reflect a broad range of GC contents: *Leptospira interrogans* GC3 ~29%, *Bacteroides thetaiotaomicron* GC3 ~45%, *Synechocystis sp.* GC ~50% and *Mycobacterium tuberculosis* with GC3 ~80%. In contrast to *E. coli* and *Bacillus*, these are all slow-growing [74]. We consider trends in both GC3 and optimal codon usage as a function of distance from the start codon. All four species show GC3 steeply going from low to high in the first ~10 codons (S13 Fig). For patterns of optimal codon usage there is no similar uniformity. *Mycobacterium* resembles *E. coli* with codon adaptation running from low to high, then gently further rising. *Bacteroides* resembles *Bacillus* with codon usage bias running high to low, again contra the ramp hypothesis. In the other two species, within the first 10 codons the regression line is negative (high to low) but not significantly so and codon adaptation more broadly appears to monotonically gently rise. These results further support the importance of low GC content in proximity to the initiating codon, and underscore that lack of generality of the *E. coli* pattern of optimal codon usage that inspired the ramp hypothesis.

The trend for AT content of the third site rather than codon optimality to be predictive of usage trends [72] is especially well reinforced when considering analysis of the trends on optimal codon usage within each synonymous block, in the first ~10 codons. Indeed, while the overall trends are opposite in *E. coli* and *Bacillus* (Fig 4A and 4B), what unifies both is that nucleotide content (for trends see Fig 4C and 4D), not codon optimality, is strongly predictive: in both species, when the optimal codon is G/C-ending there tends to be an increase in its usage moving 5′ to 3′ across the first ~10 codons, while when it is A/T-ending the trend is reversed.

Specifically, in *E. coli,* there are 5 of the 17 synonymous codon blocks nominated by Wei and colleagues [74] where the translationally optimal codon is A/T-ending. In all 5, the correlation between codon position and relative usage is negative, significantly so in 3 (S14 Fig). By contrast, of the 12 G/C-ending optimal codons, the trend is positive in 11, significantly so in 10. The one exception is serine’s TGC that shows no obvious trend. If we consider the trends being predicted by nucleotide content as being a “success”, then this pattern (16 successes, 1 fail) is highly significant (binomial test *P*-value = 0.00028). Similarly, in *Bacillus*, of 4 G/C-ending optimal codons, all show a positive slope, 3 significantly so (S15 Fig). Of the remaining 10 A/T-ending ones, 8 have a negative slope, 5 of which are significant (S15 Fig). The two exceptions are leucine’s 4-fold block and valine, neither of which are significant. Applying the same rules as in *E. coli*, this amounts to 12 in agreement with the nucleotide model and 2 against (binomial test *P*-value = 0.01). A simple model in which A/T-ending codons are highly used at 5′ ends, this decaying as one moves out of the 5′ domain, while G/C-ending optimal codons are under employed, this decaying as ones moves out, has overall strong support (28 in support, 3 against: *P*-value = 5 × 10^−6^), even though we take a conservative approach and consider any trend whether significant or not. Just considering cases where the trend is significant (at *P* < 0.05), the split is 21-0 (*P*-value = 9 × 10^−7^). Thus, codon usage trends within the first ~10 codons are predicted by nucleotide content of the synonymous base, not by translational optimality, in contradiction of the ramp model’s original claims [67].

The ramp model not only attempts to explain slow codons (non-optimal) giving way to faster codons, but it also suggests that ribosomes will gradually accelerate after the first section of the CDS [67]. There should thus be a tendency for a positive slope on optimal codon usage by the position after the initial ~10 codons. In *E. coli*, whose codon usage was employed to inspire (rather than test) the model, there may be such a tendency (Fig 4A). At the level of the codon block, all but 2 (of 17) show increasing usage of the optimal codon, the two exceptions both ending in A/T (S14 Fig). *Bacillus* data, however, strongly contradicts the model. There is no evidence for a positive trend overall and, if anything, the trend is the reverse of that predicted (Fig 4B). Indeed, considered at the codon block level (S15 Fig), the trend in the downstream section is for ribosomal deceleration (a negative slope for codon optimality) when the optimal codon is A/T-ending (9 of 10 blocks), and acceleration (positive slope) when the optimal codon is G/C-ending (3 of 4 blocks) (prediction from nucleotide content binomial test: *P*-value = 0.01, 12-2 split, 10 of 14 show deceleration, opposite of the prediction). This too suggests that in the downstream domains it is also nucleotide content, not optimality, that matters.

We conclude that the *Bacillus* data rejects all aspects of the ramp model: there is no preference for slow codons as such, no trend for early codons to be rarer than downstream and the trend after the first ~10–20 codons is not for increasing codon adaptation. As the synonymous substitution rate does nonetheless increase well beyond the limited ~10–20 codon domain, the ramp hypothesis provides no explanation for trends in *Bacillus*. In addition, while in *E. coli K*_s_ plateaus at codon 60, codon adaptation keeps monotonically increasing. For all these reasons, we reject the ramp as a viable explanation both for codon usage trends in 5′ domains and for the zone of reduced *K*_s_ in both species.

### A mutational model for *E. coli* is not falsified

**Fig 5:**
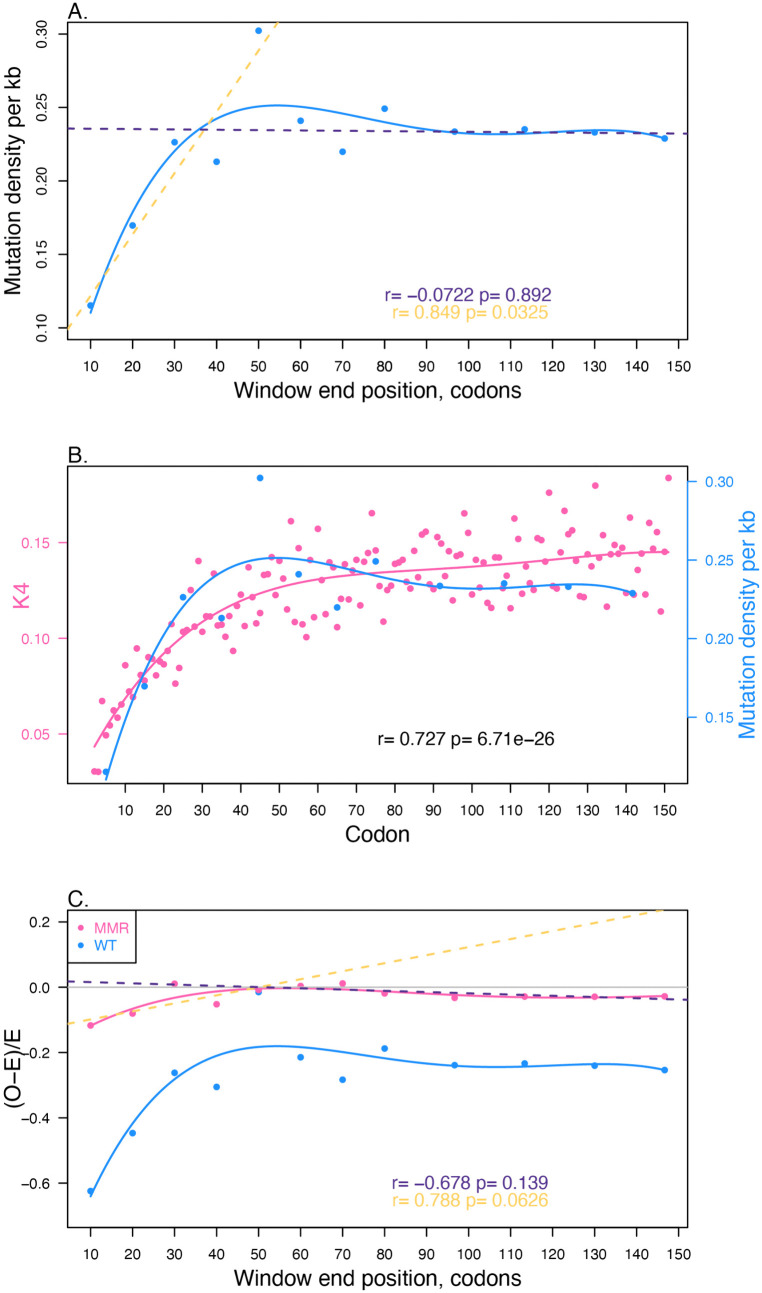
Mutation profile as a function of distance from the gene’s start. **A.** Mutation density (mutations per kilobase, kb) as a function of within gene position. The amount of sequence with each genic window, across all CDS, was determined, the density then being the number of mutations per bp, here scaled to kb. The blue line is a polynomial regression of degree 4. Yellow dashed line and yellow statistic is for the first 60 codons, dark purple dashed line and dark purple statistics is for the rest of the gene. Pearson correlation provided. **B.** Comparison of *K*_4_ values by codon and mutation density from wild-type (WT) lines. Mutation density is in blue with positions specified by middle position of the window. *K*_4_ data per codon is in pink. Lines reflect polynomial regression of degree 4. To determine pseudo-significance, we interpolate values for each codon by fitting to the blue polynomial line. These values are then correlated against the observed *K*_4_ values (Pearson correlation shown). **C.** Deviation from null (*O* − *E*)/*E* for WT (alternative metric for data in panel A) in blue and from MA lines that have MMR deleted in pink. The first 60 codons are positively correlated for the WT data (statistics as panel A), but the MMR deletion data is not (Pearson correlation *r* = 0.78, *P*-value = 0.06). Dark purple dashed line is regression for data post-60 codons for MMR-deficient data, yellow dashed line for data within 60 codons. The pink line is the polynomial regression (of order 4) for MMR-deficient, the blue for WT. The horizontal gray line marks (*O* − *E*)/*E* = 0. In all panels mutational data from Wei and colleagues [56]. The data underlying this Figure can be found in https://doi.org/10.5281/zenodo.17378284.

Although selection was the first explanation considered for the low 5′ synonymous substitution rate [39], spontaneous mutation may yet explain variation in rates. We start by employing data from MA experiments in *E. coli* [56]. We divide all CDSs in windows and for each window determine the sum number of mutations observed in that window across all genes and the sum number of base pairs within such windows. We can then determine the density of mutations per window (i.e., mutations per kb of sequence across all MA lines). We find that the first 10 codons have a density of 0.115 per kb which compares with 0.231 for the gene body (post-codon 70), the former being 50% of the latter (Fig 5A). We note that this is quantitatively similar to the ratio of the mean *K*_4_ (i.e., *K*_s_ at 4-fold degenerate sites) for the first 10 codons (excluding the start codon) and the mean of those post-70 codons (for *K*_4_, ratio = 0.0853/0.185 = 0.46). The first 60 codons have a positive slope on the line while the mutational density after that point is not significant (Fig 5A), mirroring that which is seen for *K*_4_ (Fig 5B). The same trend is seen when considering genes without overlaps (S16 Fig). We have replicated the low 5′ mutation rate using Zhang and colleagues’s [57] higher resolution spontaneous mutation data generated with Duplex Sequencing that employs 3.5 × 10^4^–3.8 × 10^4^ reads (but may be biased [59]), and MA data from Foster and colleagues [58] (S17 Fig). The same trend is not, however, so in evidence in Zhang and colleagues’s samples sequences at “lower” depth (<1,500 reads) for reasons unclear (S17D–S17F Fig). Nonetheless, these results ensure that we cannot reject the hypothesis that the *K*_s_ trends running until codon 60 is a consequence of low mutation rate. Low *K*_s_ therefore need not be indicative of purifying selection.

### Mismatch repair does not fully explain reduced 5′ mutation rates

Given the low 5′ mutation rate, the next question is what might be causing it? It is classically reported in *E. coli* that mismatch repair (MMR) is directed to the genes [58,82], this giving intergenic sequence a higher density of observed mutations (see S18C Fig). Might something similar explain the especially low rate at 5′ ends? MMR via mutS and mutL are transcriptionally coupled [83]. In transcription-coupled repair (not involving MMR), a stalled RNA polymerase is the signal for the recruitment of repair enzymes (reviewed in [84]). If we suppose there to be some constant rate at which an RNA polymerase will prematurely abort (e.g., encounter with a DNA polymerase), thus not signal 3′ errors, then it is possible that 5′ mismatches are more likely to be repaired, rather than resolved as mutations (post-replication).

To address this issue, we consider the mutational profile in MA lines that have MMR deleted [56]. This data was provided in the same experiment as our original wild-type (WT) MA data [56], thus controlling for numerous potential confounding variables. In this data, the first 10 codons have a mutational density of 10.6 per kb, which compares with 11.7 in the gene body (post-codon 60), i.e., about 90% of the rate. While the contrast between 50% (as above in the WT) and this 90% figure seems at first sight indicative of a role for MMR, caution is required in this interpretation. A statistical consequence of an absolutely higher rate is that the difference in relative mutational density between 5′ and gene body regions could trivially be lesser. Consider, for instance, that in the WT the 5′ end has a mutation rate *x* (per unit of sequence), the gene body then having a rate 2*x*. Now imagine that loss of MMR forces all sites to have 100*x* more mutations (approximately as seen). The 5′ end will then have 100*x* + *x* mutations and the gene body 100*x* + 2*x* mutations. The relative mutation rates in the 5′ end are now not 50% that of the gene body but 101/102 the rate. Thus the transition from WT having 50% of the rate to the MMR knockout having over 90% of the rate could be explained by nothing more than an equal increase in the absolute number of mutations, but with the 5′ having a lower—in absolute terms—mutation rate (the difference between 5′ and gene body would be *x* in both cases). That the ratio is ~90% not 99% could, nonetheless, be consistent with MMR operating differentially across gene bodies.

That this naïve model is incomplete is suggested by the fact that in the WT the absolute difference in mutation rate is of the order of 0.1 per kb, while in MMR-deficient lines it is ~1 per kb, i.e., not a simple addition. As the absolute difference is in fact higher in the absence of MMR, one could conclude that MMR if anything suppresses the differences between 5′ and gene body. We suggest, however, that close analysis of the dynamics of MMR would be needed to address these issues. We thus are agnostic as to effects of MMR on the 5′ v gene body difference.

What, however, is clearer is that MMR alone is not adequate to explain all the deviation seen in mutation rates. To enable comparison with the WT lines, we convert data to deviation from expected, where in each genome we consider the total number of mutations in the genome as a whole and the amount of sequence in each gene window summed across all genes. From the proportion of sequence in any given window we then derive the expected number of mutations as the product of the proportion of sequence within a window, multiplied by the total number of mutations. From this, we compute (Observed − Expected)/Expected ((*O − E*)/*E*), this being relatively insensitive to sample sizes. As can be seen, MMR-deficient lines have (*O* − *E*)/*E* values close to zero across the gene body (Fig 5C). The WT data is a transposition of that in Fig 5A with deviation scores that are highly negative in gene bodies, this reflecting MMR’s activity in gene bodies (and the consequential higher intergenic rate). While the trend in the MMR-deficient condition in the first 60 codons is marginally not significant (Pearson correlation *r* = 0.78, *P*-value = 0.07) there are significantly fewer mutations in the first window than expected by chance. There are 1,398 of 48,141 mutations that occur in CDS in the first 10 codons. These same 30 bp are 3.227% of all CDS and as such we would expect 1556 mutations if the distribution were random. The observed number is lower than this (*χ*^2^ = 16.583, *P*-value <0.00005, df = 1). If we sum all mutations across the 5′ end, only when we get to the window ending at codon 60 does the 5′ end not have fewer mutations than expected (at *P*-value<0.05), allowing for multiple testing.

### Compositional variation likely explains some of the variance in evolutionary rates

The above evidence suggests that even if MMR is more efficient in the 5′ domain, even allowing for this, there is still a lower mutational density than expected under a model of mutations being randomly distributed within genes. What else might affect this? One possibility is GC-associated mutation bias. To appraise this, in the first instance we predict, given the observed genomic mutational frequencies for each nucleotide, the mutation rates at each codon position normalized by the nucleotide content at the same position across all *E. coli* genes. Our expectation is that this positional trend is unlikely to replicate the *K*_s_ trend (that plateaus at codon 60) as it is simply going to follow nucleotide content trends (that alter at codon ~10–15) and represent their known mutability. To determine the per nucleotide rates, we determine the number of mutations for each nucleotide genomically, dividing it per occurrence of the ancestral nucleotide in the *E. coli* reference genome. We then scan each reference CDS and find the expected by-position mutation rates by multiplying nucleotide counts at each position across all genes by the observed genomic mutational frequencies previously calculated. Predictably, we find expected mutational frequencies to follow nucleotide trends of native genes, representing the known nucleotide mutagenicity (S19 Fig). Indeed, as C is known to be highly mutagenic and A to be lowly mutagenic [85], and that native genes are A-rich at 5′ ends especially in their first ~10 codons (Fig 1), the predicted mutation rates follow the 10-codon effect (S19 Fig). We see the same trend at all three codon sites (S19A–S19C Fig). This hints at why the 5′ domain might have a low *K*_s_. For potentially better resolution of mutational properties, we also consider the larger mutational sample of Zhang and colleagues [57] and observe similar trends (S19D–S19F Fig).

One key weakness with the above approach is that we have determined observed and predicted mutational rates at each codon position only considering mononucleotide context. However, as mutation rates are influenced by immediate flanking nucleotides [54,86], we also perform a trinucleotide mutation analysis considering all instances where a trinucleotide N_1_N_2_N_3_ mutates to N_1_M_2_N_3_, M being a point mutation. N_1_, N_2_, and N_3_ can be any one the four nucleotides and need not be the same as each other. We analyze all 64 possible trinucleotides across the genome.

**Fig 6:**
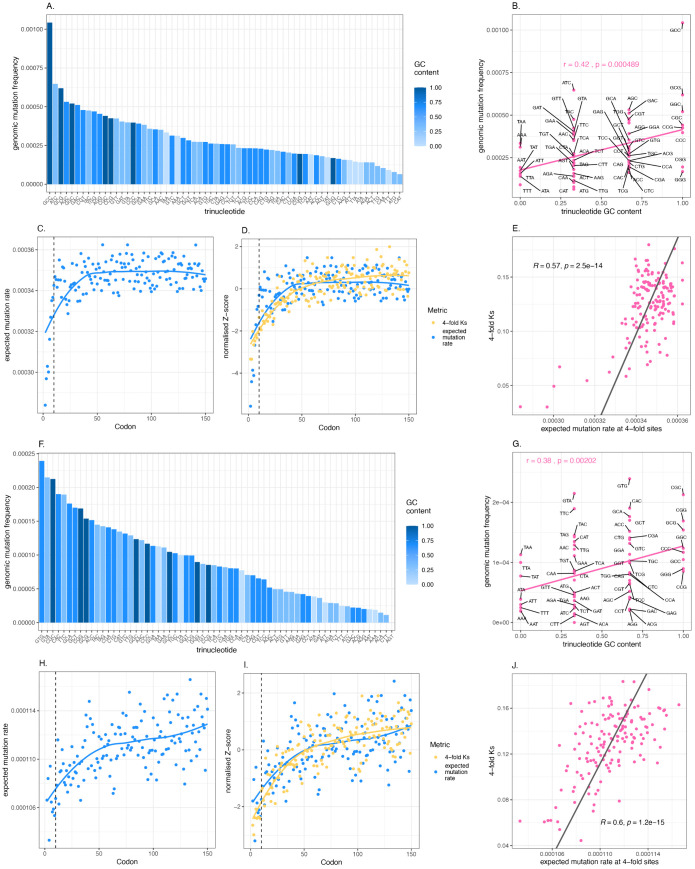
The influence of trinucleotide context on mutation and substitution. **A.** Observed genomic trinucleotide mutational frequencies rank-ordered from most to least frequent in *E. coli*. Mutational data for *E. coli* from Wei and colleagues [56]. Mutation frequency refers to mutation count per occurrence of ancestor trinucleotide. Trinucleotide mutations are such that the middle base is the mutated base. Trinucleotides on the *x* axis are rank-ordered by frequency and bars are color-coded by trinucleotide GC content. **B.** The same genomic mutation frequencies as in A, plotted against trinucleotide GC content. Line represents linear regression and Pearson correlation with respective *P*-value is also shown. **C.** Expected trinucleotide mutation rates by codon position, predicted by trinucleotide genomic mutational rates and genomic trinucleotide content. Trinucleotide mutations are such that the middle base is the mutated base, and it occurs at third sites in 4-fold degenerate codons. **D.** Comparison of expected mutational rates in C with *E. coli* conservation trends by codon position at 4-fold degenerate sites (4-fold *K*_s_, *K*_4_, as seen in S5 Fig). Both metrics are normalized by Z score. **E**. Comparison of expected trinucleotide mutation rates and *K*_4_ by position without Z transformation. Pearson correlation data is shown. Line is the orthogonal (major axes) regression line. **F–J** as A–E but for *Bacillus sp.*, with mutational data for *B. subtilis* from Sung and colleagues [62], and conservation trends at 4-fold sites in *B. toyonensis*. For C–D and H–I position on the *x* axis refers to absolute number of codons (where the start codon is position 1), and the dashed vertical black line marks the first 10 codons. Locally estimated scatterplot smoothing (LOESS) regression lines are also shown. The data underlying this Figure can be found in https://doi.org/10.5281/zenodo.17378284.

We first determine which trinucleotides are most prone to mutation by finding their genomic mutation frequencies normalized to their ancestral (pre-mutational) occurrence (Fig 6). We find that the *E. coli* genomic mutational profile is largely dominated by GC-rich trinucleotides (Fig 6A), consistent with previous findings (see, e.g., [57,87]). The most commonly mutating trinucleotide for Wei and colleagues data is GCC (Fig 6A), and trinucleotide mutation frequency is significantly positively correlated to trinucleotide GC content (Pearson correlation *r* = 0.42, *P*-value = 0.0005, Fig 6B). Considering Zhang and colleagues data, the most mutating trinucleotides are also GC-rich and especially CpG-containing, CpG indicating the dinucleotide (S20A Fig). The mutation frequency distribution is less homogeneous compared to that of Wei and colleagues data. Indeed, the four most commonly mutating trinucleotides (per occurrence of the trinucleotide) in Zhang and colleagues are CpG-containing and account for 61% of normalized mutation rates (S20 Fig). The two most mutating are GCG and CGC (accounting for 48%) which are more likely to be creating further CpG dinucleotides if flanked by G or C (S20A Fig). Here, we expand the analysis to *Bacillus* as well, and compute observed trinucleotide mutation rates for *B. subtilis* MA data [62]. We find that the tendency for GC richness among the most commonly mutating trinucleotides persists (Fig 6F and 6G).

We then ask how trinucleotide mutation rates translate in terms of expected by-position mutation rates as done above (S19 Fig) for mononucleotides. To capture solely synonymous mutation trends, we only consider cases where the mutated base occurs at a third site of 4-fold degenerate codons. We find the resulting trend (Fig 6C) to replicate the form of the *K*_s_ trend, with expected trinucleotide mutational rates starting low at the 5′ end and increasing until plateauing around codon 60 (Fig 6D and 6E). We observe the same whether we consider Zhang and colleagues’s [57] *E. coli* samples sequenced at higher (S20A–S20E Fig) or lower (S20F–S20J Fig) depth. The trends also match when considering *K*_s_ and 4-fold trinucleotide mutability in *Bacillus sp*. (Fig 6F–6J). In both species, the observed *K*_s_ and trinucleotide predicted rates are strongly correlated (Fig 6E and 6J). Observing the difference in expected trinucleotide mutation frequencies by position at 5′ ends and gene body, we see that the average mutation frequency in codons 2–10 is around 91% that of codons 60+ in *E. coli* MA data (~95% for *Bacillus*). Although this does not replicate the 50% difference seen in the observed trinucleotide mutation rates trends (Fig 5), this suggests we can use underlying trinucleotide trends to explain at least in part the lower mutational density of 5′ windows. A GC effect likely explains why in MMR-deficient lines the 5′ end still has a low mutation rate.

Our approach, extrapolating from well-resolved genome-scale data, comes with a caveat in that we have assumed that the 5′ end and CDS core have the same mutational properties, and that they are also the same as what we see genomically. We can test this in several ways, but only in *E. coli* mutational data from Zhang and colleagues as this uniquely has a high enough number of mutational calls. First, we consider the observed mutational rates at 5′ and core in *E. coli*, separating the observed mutations in the two regions (i.e., not by taking the global sample). Given the yet limited mutational sample size, we are unable to consider mutations only in the first 10 codons and thus consider the lowest round number of codons from genes’ start that allows analysis: the first 20 codons, while we consider gene cores as the rest of the CDS. We find the two mutational profiles to be significantly positively correlated (S21D Fig, Spearman rho = 0.8, *P*-value = 3.5 × 10^−13^). The correlation is also positive for Wei and colleagues [56] data though, likely due to lack of data resolution as mentioned above, not significant (S21A Fig, Spearman rho = 0.15, *P*-value = 0.44). Moreover, as above for genomic frequencies, we find the observed trinucleotide mutational frequencies given their ancestral occurrence in each gene region, and also find that the higher mutation rates tend to be in GC-rich trinucleotides (S21E and S21F Fig). We consider 5′ and core mutations at the level of the dinucleotide and consider the predicted mutational equilibrium (i.e., if the only activity was mutational bias and neutral evolution). For this, we see no difference between the 5′, the core and the global genomic predicted equilibria (*P* > 0.2 in all pairwise comparisons, S3 Table; we return to this below). We thus find no evidence for different normalized mutational profiles in the 5′ and core. The observed differences in expected mutation rates are thus better explained as owing to differences in relative frequencies of different trinucleotides, not the mutational properties of those trinucleotides.

### No robust evidence for selection for high AT from mutational equilibrium analysis

While we can account for the trend in *K*_s_ as a function of spontaneous mutation rates, can we exclude the hypothesis that synonymous mutations at the 5′ end are under selection? The transgene data strongly suggest that synonymous mutations can have a meaningful effect on protein levels (see [41–43] and Fig 1D and 1E) and the commonality of GC3 running from low to high in the first 10 codons in species with both high and low GC content (S13 Fig) is indicative of a selectively crafted GC profile.

An alternative way to ascertain whether there is selection (or biased gene conversion) acting at 5′ ends in favor of AT mutations is to determine the AT content expected at neutral mutational equilibrium and compare it to the AT content observed in native genes. Assuming the species is at nucleotide content equilibrium, an observed AT content higher than that predicted at mutational equilibrium would be consistent with selection on G/C->A/T mutations, if lower it would be consistent with selection favoring A/T->G/C mutations, while if the equilibrium is approximately the same as the observed composition then there is no need to evoke selection [54,87,88,89]. Long and colleagues [54] determined from MA data a genomic AT content at mutational equilibrium (AT*) of 0.52 for *E. coli* and therefore lower than natively observed at 5′ ends, this being consistent with selection favoring a higher AT content, the canonical model, as we discussed in the Introduction. With more data, we can now return to this issue. While we find that few mutational data sets are consistent with selection favoring G/C->A/T mutations (the canonical model), we unfortunately discover that different sets predict strikingly different equilibrium nucleotide contents, rendering strong conclusions impossible.

In *E. coli*, we estimate the equilibrium AT content to range from 0.55 to 0.89 depending on the experiment (Table 1). The highest figures (0.86 and 0.89) are derived from Duplex Sequencing approaches, but even the MA line data estimates (0.55 and 0.65) are significantly different one from another (Table 1), and all higher than the prior estimate of 0.52 [54]. The average observed AT3 content at the 5′ end is 0.55 with a maximum at any codon position of 0.62 (Table 1). If the lowest MA estimate (AT* = 0.55) is correct, then selection on G/C->A/T mutations could be evoked to account for the codon position with 0.62 AT content. If we instead believe the higher estimates, these would be indicative of selection being directed A/T->G/C, otherwise the observed AT content in *E. coli* is close to neutral on the average (i.e., around 0.55). In *B. subtilis*, MA experiments with more limited data estimate the equilibrium to be around 0.56 [54], while AT3 in the first 10 codons is a bit higher (Table 1), also consistent with weak selection favoring G/C->A/T mutations.

While these data are strikingly contradictory, we can exclude the possibility that the discrepancies are owing to problems in the methods to estimate the equilibria. Above we perform the simplest model that considers the ratio of G/C->A/T mutations per G/C to the sum of G/C->A/T mutations per G/C and A/T->G/C mutations per A/T (as performed by Long and colleagues [54], see Methods). For larger data sets, we can also consider a more complex mononucleotide-based method considering all 12 possible mutational classes and solving the relevant simultaneous equations (see Methods), as well as a dinucleotide model with a 16 x 16 mutational matrix with 256 parameters (mutations from one dinucleotide to another, per ancestral occurrence of the focal dinucleotide, see Methods). All methods applied to Zhang and colleagues’s mutational data [57] agree that the AT*~85% (for full genomic data from the samples sequenced at higher depth, AT* by simpler G/C<->A/T method = 0.86; AT* by full mononucleotide method = 0.86, AT* by dinucleotide method = 0.85, see S3 Table and S2 Text).

**Fig 7:**
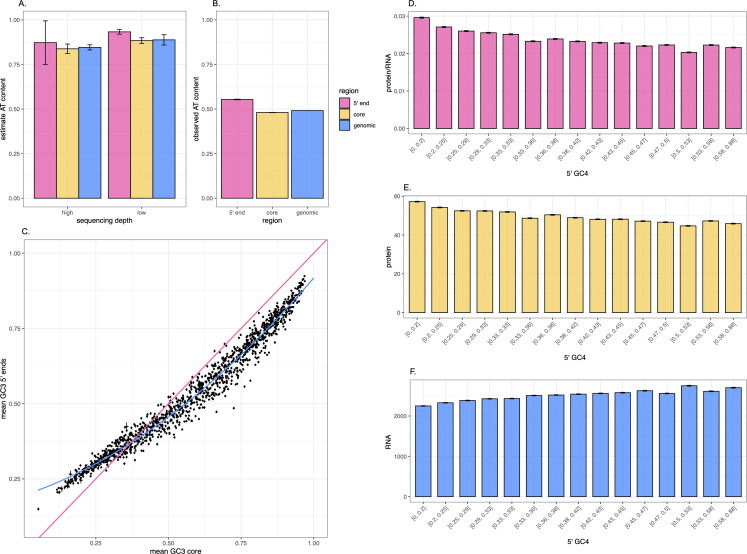
AT content within and across genomes and its influence on transgene activity. **A.** Estimated AT content expected at mutational equilibrium determined from rates of spontaneous mutation at 5′ ends, at gene cores and overall genomically in Zhang and colleagues data [57]. Nucleotide content determined using a dinucleotide approach for both samples sequenced at low and high depth, see Methods. Error bars represent standard deviation of the mutational equilibria calculation for 95% bootstrap bounds of 1,000 re-samplings. **B**. Observed AT in *E. coli* native genes. Error bars represent standard error of the mean (SEM). In both panels A and B 5′ ends are taken to be the first 20 codons and gene core is the rest of the CDS, while genomic refers to the whole genome (including non-protein-coding sequences). Legend describing gene regions applies to panels A and B exclusively. **C.** GC3 trends at 5′ ends and gene core in 1,355 bacterial species. The linear pink line represents a line of slope 1 and intercept 0 (i.e., perfect correlation between the two gene regions). The blue line is a quadratic fit to the plotted values. Error bars represent SEM. **D–F.** Expression levels of transgenes as a function of 5′ GC4 content for D. protein per RNA E. protein and F. RNA. GC4 refers to GC content at third sites of 4-fold degenerate codons. We take the 5′ end to mean the first 10 codons following the start codon. Each bin contains either 15,522 or 15,523 transgenes. Error bars represent SEM. Transgene data retrieved from Cambray and colleagues [41]. The data underlying this Figure can be found in https://doi.org/10.5281/zenodo.17378284.

While some AT* estimates above (Table 1) suggest a model of selection in favor of A/T->G/C mutations, albeit weak, this comes with the caveat that we assume the mutational biases are identical in all gene regions. We can also employ Zhang and colleagues’s higher resolution spontaneous mutation data [57] to ask whether there might be possible differences in mutational spectra in different genomic/gene regions. To this end, we derive AT* content by considering a) the global genomic mutational profile, b) the profile of mutations seen in the first 20 codons of genes, and c) the profile of mutations seen in the gene cores (everything in CDS after the first 20 codons). For the genomic AT*, we estimate a value of 0.85 ± 0.015 (standard deviation of 1,000 bootstraps), for the gene cores we estimate AT* = 0.84 ± 0.027 and for the 5′ domain AT* = 0.87 ± 0.12. All three (Fig 7A) are considerably higher than observed AT contents (Fig 7B) and none significantly different from any other (see bootstrap error bars in Fig 7A). As an additional check, we also perform the mutational equilibria test considering samples sequenced at a lower depth, and find that the predicted AT content also remains high (AT* = 0.88 ± 0.028 genomically, 0.93 ± 0.013 at 5′ ends and 0.88 ± 0.016 at cores, *P*-value = 0.54 for test of difference between the two, see S3 Table). We thus see no reason to suppose that we cannot extrapolate from genomic mutational profiles.

There are multiple possible explanations as to why AT* estimates might be so variable, none of which we can satisfactorily resolve (S2 Text). We note, however, that estimates of mutational equilibria from MA lines are relatively close to the observed AT content of intergenic sequence (*E. coli* AT* ~0.6, intergenic AT=0.58; *Bacillus* AT* ~0.56, intergenic AT=0.64; *Mycobacterium smegmatis* AT* = 0.43, intergenic AT = 0.39; Table 1). This inclines us to suppose that the MA data is more likely to be nearer the truth. Nonetheless, given the large variation in mutation equilibrium estimates, even between MA line estimates (0.55 and 0.65), the safer conclusions are that a) in this instance, the deviation from equilibrium test appears to be too highly contingent on some details of experimental protocol to make for robust inference and b) we see no robust evidence for G/C->A/T mutations being favored by selection, with alternative estimates consistent with neutrality or with selection favoring AT->GC mutations.

### High 5′ GC3 in AT-rich organisms supports selection for raised GC

If the AT content is actually lower than expected, as some values of the mutational equilibrium estimates would suggest, this would be consistent with selection in favor of A/T->G/C mutations (the opposite of the canonical model). This could mean that the process is so heavily biased at equilibrium for AT that, while higher AT and lower stability are favored at 5′ ends compared with gene cores, 5′ ends nonetheless are under selection for a non-minimized stability and non-maximized AT. Were such a model correct, we might also expect that bacteria with extreme AT pressure might evolve 5′ ends with a higher GC3 content than the gene core (most bacteria have GC3 of the 5′ end lower than that of the core [40]). We thus determine trends in average GC3 across 1,355 bacteria species (see S4 Table for species list). We confirm this expectation observing that GC3 at the 5′ end is higher than that at the core when core GC3 is lower than about 35% (Fig 7C). We note that the same effect was tested for, but not found, by Allert and colleagues, who report high 5′ AT in AT-poor genomes using a more limited sample of genomes (*n* = 816) [45].

### Selection for higher GC content is not expected to increase protein production

The next natural question is then whether higher GC content at 5′ ends might influence gene expression. Perhaps maximal gene expression is also found when the AT content is high, but not exceptionally high? To answer this, we consider again the transgene data by Cambray and colleagues [41] and ask whether those constructs with very low levels of 5′ GC at 4-fold degenerate sites produce lower levels of protein per RNA than those transgenes with slightly higher GC. We find that they do not (Fig 7D). Rather, transgene expression (protein per RNA) steadily decreases as 5′ GC4 content increases. Interestingly, this trend seems to be driven by protein levels rather than RNA, as GC4 poor constructs tend to produce the highest levels of protein (Fig 7E), though the lowest of RNA (Fig 7F). Nonetheless, our results suggest that if there is selection acting on 5′ ends towards GC, it is not simply favoring higher protein expression per RNA. Other factors such as ribosomal usage efficiency or noise reduction may also be playing a role, as previously discussed for both bacteria and eukaryotes [90–92]. This observation could also be pointing to an alternative explanation to selection for GC, as lower AT content could also simply be a result of biased gene conversion directed towards GC [87].

### Analysis of *Mycobacterium smegmatis* suggests that *K*_s_ is more likely to mislead when mutation and selection operate in the same direction

**Fig 8:**
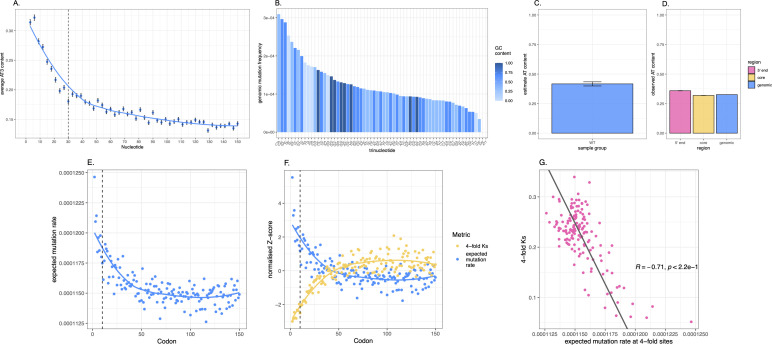
*Mycobacterium smegmatis* mutation and conservation analysis. **A.** AT content by nucleotide position in native *M. smegmatis* genes. The *x* axis represents nucleotide positions relative to the start codon (i.e., the first nucleotide of the codon after the start codon is at position 1). Error bars indicate the standard error of the mean (SEM). Dashed vertical black line marks the first 10 codons, and a locally estimated scatterplot smoothing (LOESS) regression line is shown. **B.** Observed genomic trinucleotide mutational frequencies rank-ordered from most to least frequent in *M. smegmatis*. Mutational data from Kucukyildirim and colleagues [61]. Mutation frequency refers to mutation count per occurrence of ancestor base. Trinucleotide mutations are such that the middle base is the mutated base. Trinucleotides on the *x* axis are rank-ordered by frequency and bars are color-coded by trinucleotide GC content. **C.** Estimated AT content expected at mutational equilibrium determined from rates of spontaneous mutation overall genomically in MA data from Kucukyildirim and colleagues [61]. Nucleotide content determined for WT samples using a simultaneous equations approach for mononucleotide changes, see Methods. Error bars represent standard deviation of the mutational equilibria calculation for 95% bootstrap bounds of 1,000 re-samplings. **D**. Observed AT content in the *M. smegmatis* native genome. Error bars represent SEM. 5′ ends are taken to be the first 20 codons and gene core is the rest of the CDS, while genomic refers to the whole genome (including non-protein-coding sequences). Legend color-coding gene regions applies to panels C and D exclusively. **E.** Expected trinucleotide mutation rates by codon position, predicted by trinucleotide genomic mutational rates and genomic trinucleotide content. Trinucleotide mutations are such that the middle base is the mutated base, and it occurs at third sites in 4-fold degenerate codons. **F.** Comparison of expected mutational rates in C with *M. smegmatis* conservation trends (obtained from three-way analysis with *M. goodii* and *M. septicum* as outgroup) by codon position at 4-fold degenerate sites (4-fold *K*_s_, *K*_4_, as seen in S5 Fig). Both metrics are normalized by Z score. For panels E and F, position on the *x* axis refers to absolute number of codons (where the start codon is position 1), and the dashed vertical black line marks the first 10 codons. Locally estimated scatterplot smoothing (LOESS) regression lines are also shown. **G**. Comparison of expected trinucleotide mutation rates and *K*_4_ by position without Z transformation. Pearson correlation data is shown. Line is the orthogonal (major axes) regression line. The data underlying this Figure can be found in https://doi.org/10.5281/zenodo.17378284.

In *E. coli* (and *Bacillus sp.*), it seems likely that part of the lower rate of mutation at 5′ ends is owing to compositional differences. This is in no small part because the 5′ end is AT-rich and AT residues in these two species are less mutagenic than GC-rich residues. Thus, in the case of *E. coli,* as mutational bias at the 5′ end is in the same direction as putative selection for low stability (G/C->A/T), by Ocham’s razor we should not interpret the low *K*_s_ as unambiguous evidence for selection on synonymous mutations. As seen above, however, at extremes of nucleotide content the conclusions drawn from *E. coli* need not apply: in very AT-rich genomes the 5′ ends are more GC-rich than the gene cores (Fig 7C). Potentially the most interesting case comes when the mutation bias is anticorrelated to the direction of selection. To consider what might happen in this instance, we consider an unusual species in which the mutation bias is the reverse of the common GC->AT bias. *M. smegmatis* is exceptional in that it has lost *mutL* and *mutS* and has a mutation bias that is highly AT->GC biased [61]. While it has lost two key MMR enzymes, it has gained a replacement in NucS [60]. The unusual mutation bias is likely reflected in part in it having an exceptionally high GC content (around 80% GC, Table 1). We find that its 5′ CDS is also GC-rich, just not as GC-rich as the gene core (Fig 8A). Thus, like *E. coli* and *B. subtilis*, it too has accelerating GC content (and hence decelerating AT content) moving 5′ to 3′, consistent with selection for reduced mRNA stability.

With MA mutational data [60,61] we have also determined the mutability of all trinucleotides and unusually, the GC-rich ones are not the most mutagenic (Fig 8B, Pearson correlation for trinucleotide GC content v trinucleotide mutability: *r* = 0.04, *P*-value = 0.756). The predicted mutational equilibrium of this species is an AT* of 0.43 (Fig 8C and 8D, Table 1, see also Discussion; Long and colleagues report AT* = 0.42 [54]). Employing the observed 5′ CDS, we can then predict the expected profile of substitutions under a mutation bias model (Fig 8E). Exceptionally, this then predicts a higher rate of mutation at the 5′ end, it being less GC-rich. This is supported by MA data [60,61], although the trend is much less clear than in *E. coli* (S22 Fig). Despite this, the first 60 codons have a mutation rate higher than the rest of the gene body (*χ*^2^ = 8.96, *P*-value = 0. 0027, df = 1). Thus, unlike *E. coli,* the 5′ domain appears to have a higher mutation rate owing to the reversed mutation bias, but a preference for AT richness (relative to the gene body).

If in this species *K*_s_ reflects the mutation process alone, then it should be higher at the 5′ end. By contrast, if there is selection for higher 5′ relative AT content, then selection (low *K*_s_) should be especially in evidence as it runs counter the mutation bias. We find that the profile of substitutions (*K*_s_) shows a reduced *K*_s_ in the 5′ end (Figs 8F, 8G, and S23), consistent with selection opposing the mutation bias. However, while the predicted mutational equilibrium of this species is an AT* of 0.43, through all parts of gene bodies, including 5′ ends, the observed AT is much lower than this (Table 1). This is consistent with selection, but selection (or biased gene conversion) in the direction of the mutation bias favoring A/T->G/C mutations, the strength of the effect is just weaker in the 5′ end than on the gene body. Why this species has such a dramatically high GC content in the CDS is unclear. Considering MA lines with NucS deleted, we find that variation in mutation rate across the gene body disappears, implicating mutational repair bias in the intragenic variation in mutation rate (*χ*^2^ = 0.36, *P*-value = 0.548, df = 1; S22 Fig).

## Discussion

Synonymous codon usage has a direct influence on protein levels, making it a focus in transgene design for optimizing protein production [43,45,46,93–95]. Notably, the 5′ end of the CDS in bacteria plays a particularly significant role by affecting mRNA stability and translation initiation [42–46]. Evidence for this has come from many angles, not least of which is the much-reduced *K*_s_ at 5′ ends [39], consistent with selection to preserve an AT-rich low stability mRNA. However, despite the established importance of this region, the nature of selection on synonymous sites is not as clear-cut as the simplest narrative purports, as the low *K*_s_ extends well beyond the claimed zone of influence (the first ~10 codons) to approximately codon 60 in our analysis (Fig 2). As we found no evidence to suppose that the zone of influence affecting protein level wasn’t the first ~10 codons (Fig 1), we considered a series of possible explanations. We report no evidence consistent with the gene overlap hypothesis (Figs 2 and 3) and the data from *Bacillus* reject the ramp hypothesis (Fig 4), as does the evidence from the other well-resolved bacteria that all show strong GC trends in the first few codons but no consistent pattern of bias in optimal codon usage (S13 Fig). By contrast, for *E. coli*, the best resolved case, we cannot reject a mutational model and multiple independent data sets report a low mutation rate extending through to the gene body past codon 10. The magnitude of the difference in mutation accords with the difference in *K*_s_ (Figs 5 and 6). Broadly, this result supports evolutionary models [96] that suppose that, in attempting to explain variation, be it phenotypic [97] or molecular [96], the role of mutational biases needs to be incorporated.

The causes of this low 5′ mutation rate appear in part to be a trinucleotide base mutation bias. As *K*_s_ and the mutation bias—both observed and predicted from genomic trinucleotide mutational profiles—are so well correlated, by Ocham’s razor we have no need to evoke selection to explain the *K*_s_ trend seen in *E. coli*. By contrast, in *M. smegmatis*, where the observed and trinucleotide predicted mutational trends are opposite to the *K*_s_ trend (Fig 8), here we cannot exclude selection acting on synonymous mutations at the 5′ end. More generally, the interpretation of *K*_s_ trends are problematic when the mutation bias and presumed selection bias both are in the same direction (GC->AT), as in *E. coli*.

While we conclude that even the dramatically low *K*_s_ at *E. coli* 5′ ends is not necessarily evidence for selection on synonymous mutations, the fact that *K*_s_ trends are replicated by mutational ones does not necessarily imply the absence of selection, it simply indicates that *K*_s_ may not be a sensitive or appropriate measure for it. Indeed, as exposed by the case of *M. smegmatis*, when as in *E. coli* the direction of mutation and of selection are coaligned, in the absence of selection the expected profile of the substitutional process is the same as that in the presence of selection. A further possible explanation is that if selection is weak, we expect a time lag between a mutation appearing and it being eliminated by purifying selection [98]. Employment of *K*_s_ between closely related species may not provide adequate time to resolve such weak selection, especially when the mutational and selective processes co-align. Thus, it is quite possible that there is weak selection on a multiplicity of features (RNA stability, DNA structure [99], translational optimality, etc.), while at the same time *K*_s_ is too weak a test to resolve such effects. Indeed, our result underscores the importance of considering the complex nucleotide context of synonymous sites, and associated mutational biases, when attempting to interpret *K*_s_ results and evolutionary rates more generally, as they have the potential to provide a misleading signal. Indeed, as synonymous rate estimators tend to be either mononucleotide [100,101] or codon [102] dependent, mutational determinants that span codons will not be explicitly considered, even if rate variation is permitted [103,104]. Codon pair biases are, however, biologically important and their alternation has fitness consequences [105].

While variation in mutation rate in *E.coli* may well affect synonymous substitution rates, such mutations often being under weak selection, we do not expect the mutation rate to greatly affect the rate of non-synonymous substitution as this is subject to stronger selection, this being evidenced by *K*_a_/*K*_s_ typically being much less than one (e.g., Fig 3C and 3F). Variation in the strength of selection across sites then is expected to be the dominant cause of variation of non-synonymous rates of evolution. It is nonetheless notable that the low mutation rate in affecting *K*_s_, inflates *K*_a_/*K*_s_ leading to artefactual evidence of relaxed purifying selection or an increased rate of adaptive evolution on 5′ ends.

While this presents a salutary cautionary tale, we still need to ask why, then, is GC content at the 5′ end so low (Figs 1 and 7)? While the simple model would be that GC increases RNA stability that in turn is counter-selected, A content at the most influential codon site (the second site) as regards the physicochemical properties of amino acids [76,106], is higher than at the much freer to evolve synonymous sites (Fig 1). This suggests that selection for particular properties of protein N-terminal regions may explain some of the nucleotide skew. There are indeed skews in the physicochemical properties of N-terminal regions [76,77], although there is some disagreement as to what form it takes, with some reports indicating enrichment of hydrophilic amino acids [77], some the opposite [76]. We find enrichment of hydrophilic amino acids compared to downstream (S1 Text). More generally, such skews in amino content squeeze out GC-rich runs thus forcing low synonymous rates of evolution in *E. coli*, comparable results being seen in *Bacillus* (S1 Text). The low mutability of 5′ ends in *E. coli* is explained not solely by selection on nucleotide content to enable low RNA stability, but rather, or additionally, for certain amino acid features that happen to force out mutable GC-rich runs (S1 Text).

While the *K*_s_ trend in *E. coli* and *Bacillus* appears to be driven in part by complex differential mutabilities, there may yet be selection that acts differentially on synonymous mutations at 5′ ends, as implied by the results from *M. smegmatis* (Figs 8, S22, and S23). However, if the higher AT equilibrium predictions in *E.coli* are to be believed (AT*>0.65), the nature of this selection may be the opposite of what has been presumed, i.e., it may be favoring A/T->G/C mutations rather than the opposite. Curiously, in *M. smegmatis*, the equilibrium analysis supports the same and the cross-species analysis also supports this, revealing that species with lower GC3 in gene cores tend to have higher GC3 at 5′ ends (Fig 7C). We find, however, that significantly low GC4 in transgene 5′ ends confers higher protein expression per RNA (Fig 7D), meaning that there could be additional selective pressures shaping nucleotide content at 5′ synonymous sites, potentially related to noise reduction, ribosomal retention, or maintenance of transcription factors and ribosome binding motifs [39,90–92]. An alternative explanation for a pressure to promote A/T->G/C mutations is biased gene conversion, but why this would operate more strongly on gene cores than 5′ ends is less than transparent.

At least one further set of enigmas is unresolved by our analysis, these concerning trends on codon adaptation extending in the gene body. In *E. coli*, codon adaptation increases monotonically up to codon 150 (where it doesn’t asymptote, Fig 4A). By contrast, in *B. subtilis,* after the 10 codon initial section, codon adaptation is flat (Fig 4B) and, when seen at the codon block level (S15 Fig), any weak trends accord with optimal codon A/T termination, with A/T being avoided towards gene cores and G/C being favored. These trends are not obviously anything related to a ramp, not least because the ramp predicts increasing codon adaptation not seen in *B. subtilis* (or *Bacteroides*). In both *E. coli* and *Bacillus* these trends are largely uncoupled from *K*_s_ trends. In part, these trends may be considered to reflect different utilization of optimal codons, *E. coli* being overall well optimized, *B. subtilis* less so [81], despite both being considered to be fast replicators [74]. Why this is remains obscure. Understanding this difference and why codon adaptation monotonically increases in *E. coli* will likely hold important truths about the biology of these two species. For now, we suggest the discrepancy to not seem to be attributable to the mutational profile in *B. subtilis,* as data from MA experiments show similar trends as in *E. coli* (Fig 6). Similar analysis in other bacterial species would permit further generalization of the explanation of the reduced *K*_s_ rate until codon 60.

Perhaps the greatest enigma revealed by our analysis is the diversity of equilibrium estimates, for which there may be multiple causes (S2 Text). For example, deep Duplex Sequencing may find errors that will not be resolved as mutations as they may have been repaired after the sequencing has been performed, a problem unlikely to affect accumulated mutations down MA lines. However, we see that in the deepest analyses the (more MMR affected) CDS still has lower mutation rates than intergenic sequences, consistent with repair-resolved mutations (S2 Text). Analysis of the best resolved mutations confirms the same (S2 Text). As we cannot resolve the causes of the variation, in the interim, we suggest that the current evidence does not robustly support a model of selection for high AT content, this also being consistent with what is seen in the AT-rich bacteria (Fig 7), but this issue requires further verification. That in *E.coli* we observe *K*_s_ trends aligning with mutational data regardless of the mutation experiment design (Duplex Sequencing, MA), seen also for predicted mutational profile in *Bacillus* (Figs 6 and S20), suggests that the variation between data sets in mutation equilibrium predictions does not invalidate our suggestion that *K*_s_ may not be a reliable metric for reporting selection on synonymous sites when selection and mutation bias align. This is likely because in all data sets in *E. coli* and *Bacillus,* the GC-rich runs are the most mutagenic, there is just disagreement about how much more mutagenic. Note also that mutation equilibrium estimates are based on G/C<->A/T mutation counts, while *K*_s_ trends will also factor in A<->T and G<->C mutations.

That estimates of mutational equilibria from MA lines are relatively close to the GC content of intergenic sequence (*E. coli* AT* ~0.6, intergenic AT 0.58; *Bacillus* AT* ~0.56, intergenic = 0.64; *M. smegmatis* AT* = 0.43, intergenic 0.39: Table 1) inclines us to suppose that the MA data is more likely to be nearer the truth than the results from Duplex Sequencing. More generally, here we have restricted analysis to consideration of the relationship between variation in *K*_s_ across genes and mutability of the trinucleotides. We can also ask about whether similar problems will impact inferences of evolutionary rates of other classes of sequence, the most comparable of which is likely to be intergenic DNA, non-synonymous mutations commonly being under stronger purifying selection, as evidenced by low *K*_a_/*K*_s_ ratios [107] (although see Figs 2 and 3 for incidences where classical *K*_a_/*K*_s_ has the potential to mislead owing to distorted *K*_s_). As regards the absolute rates of mutation of non-coding sequence, while above we have controlled for trinucleotide content in comparing relative rates of mutation, this partially disguises a secondary effect, namely genic sequences in *E. coli* have a higher GC content than intergenic sequences (Table 1) and so would be expected to have a raw mutation rate that, if all else were equal, would be higher than in intergenic sequences. More generally, the relative trinucleotide content of intergenic DNA and CDS are moderately correlated, as expected given selection on amino acid content (correlation of trinucleotide occurrence rates in intergenic sequences versus genic sequences, Pearson correlation *r* = 0.39, *P*-value = 0.0016, S18C Fig). The relative evolutionary divergence of non-coding DNA will then be a complex interplay of a lower mutation rate owing to their relative AT richness, a higher mutation rate owing to lesser repair, purifying selection, for example, on ribosomal binding sites and non-coding RNAs [108], and possibly adaptive evolution in promoters [108]. Allowance for trinucleotide differential mutability in the interpretation of divergence data would be important in this context too.

## Methods

### Nucleotide content trends by codon position in native genes

Reference genomes were retrieved from RefSeq NCBI [109]. For *E. coli*, native gene analysis were performed on the reference genome downloaded on 13th September 2023 with accession GCF_000008865.2. For *B. subtilis*, the reference genome was downloaded on 16th May 2025 with accession GCF_000186085.1, and for *B. toyonensis* on 16th January 2025 with accession GCF_016605985.1. For *M. smegmatis*, the reference genomes were downloaded with accession GCF_000283295.1.

All genes underwent the necessary sequence checks: starting with a start codon (any allowed by translation table 11 [110]), ending in a stop codon, being in multiple of three, containing only canonical bases and no internal stops. Average nucleotide content was then determined for each codon position along the CDS, with division into the three codon sites. For 4-fold content, the average content for each nucleotide was determined as a proportion of all 4-fold degenerate sites.

### mRNA stability prediction for native genes

mRNA stability was predicted using the ViennaRNA R package v 2.0 [55] using a sliding window approach with windows of 30 bp and shifts of 3 bp (to give a by codon mRNA stability prediction). The analysis was conducted from base pair -15 until codon position 150. This was conducted for all *E. coli* native genes and an average per codon position was determined.

### Evaluating impact of codon position on transgene expression levels

All protein and RNA transgene expression data, as well as transgene sequences, was retrieved from Supplementary Data 15 of Cambray and colleagues [41]. PNI was used as protein measure and RNA_SS_ as RNA measure. The R package relaimpo (v 2.26) [78] was used to assess the relative influence of GC content on PNI per RNA_SS_ (protein/RNA) for each 5′ codon position until 20 codons after the start codon (with lmg model protein/RNA ~ GC_codon_2 + GC_codon_3, etc.). This approach quantifies the contribution of each predictor variable while accounting for covariance among them. The analysis was also repeated using protein only rather than protein/RNA.

### Correlation and partial correlation analysis

The Spearman correlation between PNI/RNA_SS_ and GC content was found by codon position for the first 32 codons following the start codon (maximum length of sequence provided by Cambray and colleagues for each of their 244,000 constructs) using the rcorr function in the R package Hmisc [111]. A partial correlation was also determined to examine the effect, while accounting for the influence of all other codon positions, using the R package ppcor [112]. Similarly, a correlation and partial correlation analysis was performed between GC content and cell fitness (Cambray and colleagues’s W_RI_ [41]), and CAI values taken to represent the enrichment of a codon relative to its synonyms in the 10 codons at genic cores in highly compared to lowly expressed native genes (nHEGs v nLEGs) according to amassed protein abundance data [113]. CAI is represented through log odds ratios such that a higher value means higher usage in nHEGs.

The same packages were used to determine Spearman correlation and partial correlation between predicted mRNA stability and transgene expression. mRNA stability was predicted using the ViennaRNA R package v 2.0 [55]. Correlation with expression was separately found between stability of three transgene sequence regions (codons 2–11, 12–21, and 22–31), provided by Cambray and colleagues [41].

### Calculation of optimal codon enrichment trends

Wei and colleagues [74] (their Table 1) provide a list, for *E. coli* and *B. subtilis* separately, of translationally optimal codons for any given block of codons coding for the same amino acid. Unusually, they determine the optimal codon by reference to the abundance of iso-acceptor tRNAs within the transcriptome (as opposed to copy number [75]) and require that the nominated optimal codon confirm with classical patterns expected of optimal codons (HEG enrichment, etc.). Their metric is thus comparable to tAI [75,114,115] but outperforms prior measures. They nominate 17 such optimal codons in *E. coli* and 14 in *B. subtilis*. As in *E. coli*, they could define one for the 3-fold degenerate isoleucine block, their codons belong to groups that are either 2-, 3-, or 4-fold degenerate. In *B. subtilis*, groups are either two- or 4-fold degenerate. We make the classical presumption, as assumed by the supporters of the ramp hypothesis [67], that codon optimality equates to faster processing.

To define the degree of usage of optimal codons in any set of codons (e.g., in a CDS), we implement the following approach. For every codon nominated as optimal, we recover both its identity (from their Table 1 [74]) and the degree of redundancy of the block of synonyms. We add to a list of optimal codons the nominated optimal codon and add to a list of qualifying codons all codons within in the same block (i.e., that code for the same amino acid). For example, if TGC is nominated as optimal for cysteine, TGC is added to the list of optimal codons, and both TGC and TGT are added to the list of qualifying codons, and both classed as belonging to the 2-fold degenerate class of codons. Blocks with no nominated optimal codon are ignored.

We then consider any relevant list of codons and score optimal usage within this list. For example, to determine 5′ to 3′ codon optimality trends in native genes, we employ all second site codons, all third site codons, etc. up to codon 150 for genes longer than 450 bases. In this instance, all second site codons are added to one list, all third site codons their own list, etc. For each such list, we consider each codon in turn and ask whether it exists within the list of qualifying codons for that species. If it does, we add one to the count of codons of that codon’s block degeneracy (*d*) giving us a count of the total number of qualifying codons of each relevant degeneracy (*T*_d_). If it belongs to the optimal codon group, we add one to the count of optimal codons for the relevant degeneracy (*O*_d_). For example, if, as in *E. coli*, TGC is optimal for cysteine, each time we encounter TGC we add one to the optimal codon count of 2-fold degeneracy and one to the list of 2-fold qualifying codons. Occurrence of TGT increments the latter count and not the former. At the end of the process, *T*_d_ is a count of the total number of qualifying codons of degeneracy *d* and *O*_d_ the count of optimal codons of degeneracy *d*.

The meaning of counts of *O*_d_ of different degeneracy has different meanings dependent on degeneracy: a null expectation is that 4-fold optimal codons will be proportionally employed (*O*_d_/*T*_d_), just less than 2-fold ones because there are more alternatives for 4-fold codons. Thus, for each degeneracy block of class *d*, we calculate deviation, *D*_d_ = (*O*_d_ − (*T*_d_/*d*))/(*T*_d_/*d*). In principle, unlike *χ*^2^, this metric should not be biased by sample sizes. As a simple example, if we record 100 usages of the optimal codon in all 4-fold degenerate groups with 400 instances of usages of any 4-fold degenerate codon, the *O*_d_ = 100, *T*_d_ = 400, *d* = 4, so we record *D*_4_ = 0. To determine the mean deviation, we considered the weighted mean *D* value across all applicable degeneracy classes (*d* = 2,3,4 for *E. coli*, *d* = 2,4 for *B. subtilis*). Thus, for *B. subtilis,* weighted mean (*D*) = (*D*_4_.*T*_4_ + *D*_2_.*T*_2_)/(*T*_4_ + *T*_2_). Employment of weighted means is desirable as the proportion of 4-fold degenerate codons varies by codon position. The weighted mean provides our metric of the usage of optimal codons for any given list of codons.

We repeat these analysis for the four others species whose optimal codons are defined using the genome accession numbers as provided by Wei and colleagues [74] (their Table 2) i.e., *Mycobacterium tuberculosis* (NC_000962), *Synechocystis sp.* (NC_017277), *Bacteroides thetaiotaomicron* (NC_004663), and *Leptospira interrogans* (AE016823).

### Orthologous database retrieval

To perform a conservation analysis, RefSeq genomic coding and protein sequences were firstly downloaded from NCBI [109]. For gram-negative bacteria: *Escherichia coli* (GCF_000005845.2), *Escherichia fergusonii* (GCF_020097475.1), and *Salmonella enterica* (GCF_000006945.2) downloaded on 18th October 2024. For gram-positive bacteria: *Bacillus toyonensis* (accession: GCF_016605985.1), *Bacillus anthracis* str. ‘Ames Ancestor’ (accession: GCF_000008445.1), *Bacillus mycoides* (accession: GCF_000832605.1) downloaded on 16th January 2025. For Mycobacteria: *Mycobacterium smegmatis* (accession: GCF_000283295.1), *Mycobacterium goodii* (accession: GCF_022370755.2), *Mycobacterium septicum* (accession: GCF_046506965.2). Bacterial proteomes were processed with OrthoFinder (v 2.5.5) under standard configurations [116]. The species tree utilized for posterior *K*_a_/*K*_s_ calculations was directly obtained in this step from the OrthoFinder output [117].

### Alignment of orthologous sequences

Putative orthologous protein sequences were matched to the corresponding RefSeq CDS using their protein IDs. Subsequent three-way alignments were performed for each orthogroup corresponding to single-copy genes using MAFFT (v 7.526) under standard configurations for nucleotide and protein sequences [118]. Codon-based alignments were performed using PAL2NAL (v 14) software [119]. All aligned orthologous genes then underwent necessary sequence checks: starting with a start codon (any allowed by translation table 11 [110]), ending in a stop codon, being in multiple of three, containing only canonical bases and no internal stops. We also limited the analysis to orthologous genes that were at least 180 codons long (to see trends in the first 150 codons without capturing 3′ effects). The orthologs that passed checks were then used to reconstruct the ancestral state between *E. coli* and *E. fergusonii* (with *S. enterica* as outgroup), and between *B. toyonensis* and *B. anthracis* (with *B. mycoides* as outgroup) for gram-negative and gram-positive bacteria, respectively. We used a codon-based maximum likelihood approach implemented in iqtree2 [120] under -asr mode. We repeat the analysis for *M. smegmatis*, reconstructing the ancestral state between it and *M. goodii* (with *M. septicum* as outgroup).

### Conservation analysis by codon position

For our conservation analysis, we consider *E. coli* and the ancestral state, *B. toyonensis* and the ancestral state, and *M. smegmatis* and the ancestral state. To determine conservation by codon position, we separately extract each codon for each ortholog and concatenate it to the codons at that same position for all other orthologs. We do this for the first 150 codons following the start codon. This results in 150 codon-specific files. We exclude cases where all three species have different codons, as it is likely for there to be uncertainty in the ancestral sequence reconstruction at those positions. We repeat the process extracting only those codons coding for 4-fold degenerate amino acids. Since not all orthologous genes have 4-fold degenerate codons at every position, the resulting codon-specific files vary in sequence lengths. We then determine the synonymous (*K*_s_) and non-synonymous (*K*_a_) nucleotide substitution rates, and the ratio between the two (*K*_a_/*K*_s_) using the CODEML program in PAML (v 4.10.7) [121]. Performing the process for each codon-specific file allows us to then assess substitution rates per codon position.

### Conservation analysis for non-overlapping genes

We repeat the conservation analysis considering only genes that do not present overlaps. We consider reference genome annotations and repeat the same conservation analysis as described above for orthologous genes only considering those that are not overlapping.

### Conservation analysis of operonic and non-operonic genes

Annotation of operonic genes for *E. coli* is from Mao and colleagues [122] with the data file downloaded from Supplement File 1. *E. coli* annotation is from GCF_000005845.2 to correspond. Seven genes are called in the operon file that aren’t in the reference genome. By manual inspection, these were present in NC_000913.3 but have been discontinued. *B. subtilis* operon annotation was from Geissler and colleagues [123] and downloaded from https://zenodo.org/records/4305872. From file BSGatlas-v1.1.xlsx, we employed the operon sheet of the Excel file and converted to csv. We obtained the corresponding genome file (CDS) for ASM904v1 from NCBI [109] along with the annotation GFF file.

### Mutation accumulation and spontaneous mutation data retrieval

We employ different sources of MA data for *E. coli*. Our main analysis employs that of Wei and colleagues [56], which contains mutation records for both WT and MMR-deficient *E. coli* (RefSeq accession GCF_000005845.2). We also employ MA data of Foster and colleagues [58] merging mutation records from three *E. coli* strains PFM2, ED1a, and IAI1 (all considered WT and referred back to the same reference genome, GCF_000005845.2). We obtain spontaneous mutation data from Zhang and colleagues [57], who use *E. coli* strain ATCC 8,739 (RefSeq accession GCF_026016785.1). Their experimental design involves parallel samples grown from separate single colonies which are then sequenced via Duplex Sequencing at very high depth, allowing them to output a large catalog of spontaneous mutations across the whole *E. coli* genome. Zhang and colleagues data contains 12 independent samples, all of which are sequenced with “lower” depth (>1,500) and two of which are also sequenced at “higher” depth (3.5 × 10^4^–3.8 × 10^4^) [57]. Except for where it is indicated, e.g., predicting AT content at mutational equilibrium, we perform our main analysis with those higher depth samples as deeper sequencing allows to capture even the rarest mutations before selection had any filters on them. We employ another set of spontaneous mutation data recorded via Duplex Sequencing by Bhawsinghka and colleagues (for *E. coli* GCF_013166975.1) [59].

We additionally perform mutational data analysis for *B. subtilis* (GCF_000186085.1) employing MA data from Sung and colleagues [62]. For *M. smegmatis* we combine WT MA data from Kucukyildirim and colleagues [61] and Castañeda-Garcia and colleagues [60] (GCF_000283295.1). From Castañeda-Garcia and colleagues we also employ MA data for MMR-deficient *M. smegmatis.*

### Analysis of mutation trends along the CDS

To determine observed mutational trends along the CDS, we divide all CDSs into fixed-size windows. For each window, we find its mutation density as the total number of mutations observed across all genes normalized by the total number of base pairs within that window (i.e., mutations per kb of sequence). Where possible, we do this both for WT and MMR-deficient mutation data (see above for data retrieval and RefSeq accessions of reference data downloaded from NCBI [109]).

To enable better comparison with the WT lines, we also convert data to represent deviations from expected values. For each genome, we first consider the total number of observed mutations in the entire genome and the total sequence length of each gene window summed across all genes. We then calculate the expected number of mutations in a given window as the product of the window’s proportion of the total sequence and the total number of mutations. We subsequently compute the deviation as (Observed − Expected)/Expected (i.e., (*O* − *E*)/*E*). As this metric expresses relative deviation, it is largely independent of differences in total mutation counts or sample sizes across genomes.

We next consider mutational trends relative to the sequences’ underlying nucleotide content. We first determine observed trinucleotide mutation frequencies, where the mutated base is the middle base of the trinucleotide. This allows to control for flanking nucleotides. For each of the 64 possible trinucleotides, we find the mutation counts and divide them per occurrence of the trinucleotide to give the trinucleotide mutation frequencies. Given the limited mutational sample size, we are unable to find observed frequencies for trinucleotides by codon position or codon site. For Wei and colleagues [56] and Zhang and colleagues [57] data, we instead differentiate between mutations at 5′ ends (first 20 codons following the start codon) and those gene cores (rest of the CDS). We also consider the total genomic trinucleotide mutation frequencies.

Although higher data resolution would be required to directly assess mutation frequencies by codon positions or codon site, we can infer positional trends along the CDS by estimating the expected mutation frequencies at each codon position based on the observed genome-wide mutation frequencies. In order to do this, we first determine the genomic frequencies by finding the number of mutations for each mononucleotide and dividing it per occurrence of the ancestral base in the *E. coli* reference genome. We then scan each reference CDS and find the expected by-position mutation rates by multiplying nucleotide counts at each position by the observed genomic mutational frequencies previously calculated. We also perform the same test for trinucleotides where the mutated (central) base is at third sites of 4-fold degenerate codons.

As repair is directed to the genes, we also ask whether the mutation rate of any given central base of each of the 64 possible trinucleotides is higher in intergenic sequence compared to annotated genes. We consider anything annotated within the full *E. coli* reference genome as a possible gene, including RNA, and define intergenic as the regions between these (extracted using Gff-Ex v2.3 [124]). For both classes of sequences, we determine using bedtools (v 2.31) [125] (via pybedtools v 0.12.0 [126]) whether a mutation was intergenic or genic, and compute for each trinucleotide their rate of mutation per occurrence of that trinucleotide in the relevant sequence class. We thus derive two 64-element vectors (each entry being a trinucleotide), one for normalized mutation rates for genic and one for intergenic sequence. We then compare via paired *t* test.

We determine trends in observed and expected mutation frequencies for *E. coli*, *B. subtilis*, and *M. smegmatis* (see above for data set information).

### Native trends in trinucleotides

Analyses on native trinucleotide usage were performed on the reference genome downloaded for *E. coli* ATCC 8,739 on 28th November 2024 from RefSeq NCBI [109] with accession GCF_026016785.1, as linked to mutational data from Zhang and colleagues [57]. For each codon position, we find the summed content of trinucleotides ANT, ANA, TNT, TNA (where N is any of the four nucleotides). We consider the instances where the middle base of the trinucleotide is located at codon third sites, and we retain the codon position of that base to find trends along the CDS.

### Analysis of amino acid chemical properties by codon position

We determine the average score at each codon position across eight amino acid chemical properties, four that Jin and colleagues [76] have previously found to be associated to second sites (hydropathy, chemical composition of the side chain, molecular volume, polarity), and four that supposedly aren’t associated (molecular weight, melting point, isoelectric point, refractivity). We first retrieved the reference scale for each property: hydropathy scores determined according to the Kyte–Doolittle scale where low values represent hydrophilic amino acids (see Table 2 in [127]). Scales were retrieved from [128] for isoelectric point, defined as the pH at which the amino acid loses the electric charge, melting point, polarity, molecular volume, and chemical composition of the sidechain, defined as the atomic weight ratio of hetero (noncarbon) elements in end groups or rings to carbons in the side chain. For molecular weight, scale retrieved from [129]. Finally, for refractivity, defined as the amount of refraction per gram of amino acid, the scale was retrieved from McMeekin and colleagues [130] in Jones [131].

For each chemical property, we find the average index for each codon position taking into account the observed amino acid frequencies at that position across native genes of *E. coli* (reference genome downloaded on 25th November 2024 from RefSeq NCBI [109], accession: GCF_000008865.2). We also find the average index for each codon position taking into account the amino acid frequencies expected from mononucleotide, calculated as probabilities based on the frequencies of mononucleotides found at the codon third site of 4-fold degenerate amino acids. A comparison between observed and expected allows to determine whether observed trends are present just by considering the codon distribution that occurs by chance (expected).

### Predicting AT content at mutational equilibrium

The neutral AT equilibrium can be estimated using a simple method which involves finding the relative mutation rates of G/C to A/T and A/T to G/C. However, we also apply a more comprehensive approach also used by Rice and colleagues [132] that treats each base as an independent state and allows to determine the equilibrium frequencies of all four nucleotides while also accounting for nucleotide skews [133]. The method can be illustrated by consideration of the mononucleotide model. We for instance define *G* as the frequency of G and *T* as the frequency of T. The mutation rate from G to T is denoted as G2T, expressed per occurrence of the ancestral base. We describe frequencies for each nucleotide N after a given period (*N′*) and, to determine equilibrium frequencies, we solve for conditions where *N′* = *N*, leading to the following equations:

*G* (1 − G2T − G2C − G2A) = *A* (A2G) + *T* (T2G) + *C* (C2G)

*C* (1 − C2T − C2G − C2A) = *A* (A2C) + *T* (T2C) + *G* (G2C)

*A* (1 − A2T − A2C − A2G) = *G* (G2A) + *T* (T2A) + *C* (C2A)

*T* (1 − T2G − T2C − T2A) = *A* (A2T) + *G* (G2T) + *C* (C2T)

Here, the left-hand side of each equation represents the loss rate given the current nucleotide abundance, while the right-hand side represents the gain rate at equilibrium (i.e., we solve for the state where gain = loss). The 12 flux parameters (i.e., G2T, C2G, etc.) are derived from the mutation profile, calculated as the observed number of mutations per occurrence of the ancestral nucleotide. For any given mutational matrix, we solve for four simultaneous equations, ensuring that one nucleotide frequency is determined as 1 minus the sum of the other three (e.g., *T* = 1 − *A* − *C* − *G*). The equations are solved using NumPy [134]. To resolve the ambiguity of which strand the mutation is happening on, we consider A + T and G + C expected content at equilibrium rather than the four nucleotides separately.

To estimate confidence intervals, we perform a bootstrap resampling procedure, drawing different sets of mutations with replacement from the original set of mutations. We repeat this 1,000 times. For each resampled data set, equilibrium frequencies are recalculated, allowing us to establish confidence bounds.

For the central analysis, we expand this approach and consider 16 × 16 mutational matrixes including each dinucleotide mutating to each other, rather than mononucleotides. This results in 15 simultaneous equations which we solve for. For equations and means to solve them see scripts at https://doi.org/10.5281/zenodo.17378284.

We repeat the whole process for 5′ ends (which, due to data sample limits, we take to be first 20 codons following the start codon, i.e., the lowest round number that allows analysis), gene cores (the rest of the CDS), intergenic regions (mutations with annotated positions that fall in between CDSs), as well as genomically (including everything, also non-protein-coding sequences). For completeness, we perform the analysis for both those samples in Zhang and colleagues [57] that were sequenced with a higher and lower depth (S3 Table). The other mutation data sets described above lack sufficient resolution for a by region analysis.

As a sanity check, we also perform the test through the simpler G/C<->A/T approach considering mutations that were called with different significance (that Zhang and colleagues [57] report). We divide the mutations by quartiles of mutational calling *P-value* (Q1 meaning lowest *P-value*, i.e., higher significance), and find the estimate neutral AT content for each mutation group separately (S2 Text).

We perform a further test repeating the analysis employing a separate data set by Bhawsinghka and colleagues [59], who collect spontaneous mutation data with Duplex Sequencing in WT *E. coli* as well as mutants defective in stress response-related mechanisms (mutL and mutT). For this data, we compute mutation equilibrium AT content using the full simultaneous equations method considering mononucleotides, as explained above.

We estimate mutational equilibrium nucleotide content through the simplest mononucleotide method for *E. coli* MA data [56,58], as well as *E. coli* spontaneous mutation data recorded via Duplex Sequencing [57,59]. For *B. subtilis*, using data from Sung and colleagues [62], and for *M. smegmatis*, combining data from Kucukyildirim and colleagues [61] and Castañeda-Garcia and colleagues [60]. See Table 1 for all AT* results computed using the simple method. For *M. smegmatis* merged data, we also compute AT* using the full simultaneous equations method based on mononucleotide changes (Fig 8C).

### Cross-species nucleotide content by codon position

For the analysis of 5′ and core GC3 content across bacteria species, we firstly retrieved all reference CDSs for 1,355 bacterial species (see S4 Table for full list of species names and accession). Sequences were downloaded on 13th September 2023 from RefSeq NCBI [109]. These represent a selection of all the available reference genomes on RefSeq NCBI, keeping one species per genus. For each species, we then found the average GC content at codon third sites across all genes for 5′ ends (first 10 codons following the start codon) and gene cores (rest of the CDS).

### Comparison between transgene GC4 content and expression

For the analysis of 5′ GC4 content and transgene expression, we used data from Cambray and colleagues [41], as above. For each transgene, we found the average GC content at third sites of 4-fold degenerate codons for the first 10 codons following the start codon. We also retrieved an expression measure as protein per RNA where PNI was used as protein measure and RNA_SS_ as RNA measure (see Supplementary Data 15 in Cambray and colleagues [41]).

### Z score normalization

For value normalization, we use a *Z* score approach, i.e., (observed − mean)/standard deviation.

## Supporting information

S1 Fig Nucleotide content trends across 5′ codons in native *E. coli* genes. For all four nucleotides, content is averaged at each nucleotide position across 5,098 native genes. The *x* axis represents nucleotide positions relative to the start codon (i.e., the third nucleotide of the start codon is labeled as position 0). Error bars indicate the standard error of the mean (SEM). Dashed vertical black line marks the first 10 codons. Locally estimated scatterplot smoothing (LOESS) regression lines are included. The data underlying this Figure can be found in https://doi.org/10.5281/zenodo.17378284.
(PDF)

S2 Fig The extent to which codon GC content by position is associated with protein level. As with in-text Fig 1D–1F, except that the metric is protein level not protein per RNA. **A**. Relaimpo analysis. The model explains 7.4% of the variation in protein level. **B**. Spearman correlation analysis. **C**. partial Spearman correlation. The data underlying this Figure can be found in https://doi.org/10.5281/zenodo.17378284.
(PDF)

S3 Fig Spearman correlations between cell fitness and GC content or CAI in transgenes for each 5′ codon position. **A.** Full Spearman correlations for GC content. **B.** partial Spearman correlations for GC content controlling for the influence of all other codon positions in the available construct sequence (codons 2–30) such that, for instance, the relation between transgene expression and GC content at codon 2 is found independently of the relation with GC content in codons 3–30. **C, D** same as A, B, but for Codon Adaptation Index (CAI) at each position. Here, CAI measures the enrichment of a codon relative to its synonyms in the 10 codons at genic cores in highly compared to lowly expressed native genes (nHEGs v nLEGs) according to amassed protein abundance data. CAI is represented through log odds ratios such that a higher value means higher usage in nHEGs. In all plots, colored points represent rho values with a *P*-value ≤ 0.05, while gray points are non-significant. Locally estimated scatterplot smoothing (LOESS) regression lines are included. Codon positions on the *x* axis refer to absolute codon numbers (e.g., the start codon is codon 1). Dashed vertical black line marks the first 10 codons. Transgene data from Cambray and colleagues [41]. The data underlying this Figure can be found in https://doi.org/10.5281/zenodo.17378284.
(PDF)

S4 Fig Substitution rates by 5′ codon position comparing the *Escherichia coli–Escherichia fergusonii* ancestor to *E. fergusonii*. A. Synonymous substitution rates (*K*_s_); **B.** non-synonymous substitution rates (*K*_a_), and **C.** the ratio between the two (*K*_a_/*K*_s_). The *x* axis represents absolute codon position (i.e., the start codon is codon 1). A–C plots include orthologs that are at least 180 codons long (*n* ~ 1,400). Dashed vertical black line marks the first 10 codons. Locally estimated scatterplot smoothing (LOESS) regression lines are included. The data underlying this Figure can be found in https://doi.org/10.5281/zenodo.17378284.
(PDF)

S5 Fig Substitution rates by 5′ codon position for 4-fold degenerate codons comparing the *Escherichia coli–Escherichia fergusonii* ancestor to *E. coli*. A. Synonymous substitution rates (*K*_s_); **B.** non-synonymous substitution rates (*K*_a_), and **C.** the ratio between the two (*K*_a_/*K*_s_). The *x* axis represents absolute codon position (i.e., the start codon is codon 1). Dashed vertical black line marks the first 10 codons. Locally estimated scatterplot smoothing (LOESS) regression lines are included. The data underlying this be found in https://doi.org/10.5281/zenodo.17378284.
(PDF)

S6 Fig Substitution rates by 5′ codon position comparing the *Escherichia coli–Escherichia fergusonii* ancestor to *E. coli*. A. Synonymous substitution rates (*K*_s_); **B.** non-synonymous substitution rates (*K*_a_), and **C.** the ratio between the two (*K*_a_/*K*_s_). A–C plots include operonic orthologs that are at least 180 codons in length (*n* = 1,080). **D–F.** Same as A–C but only including non-operonic orthologous genes that are at least 180 codons in length (*n* = 473). For A–F panels, the *x* axis represents absolute codon position (i.e., the start codon is codon 1). Dashed vertical black line marks the first 10 codons. Locally estimated scatterplot smoothing (LOESS) regression lines are included. Note that codon position here is by reference to the codon position in the alignment. **G.** The *K*_s_ trends seen in operonic (A) and non-operonic (D) genes plotted against each other. Orthogonal regression line and Pearson correlation shown. The data underlying this Figure can be found in https://doi.org/10.5281/zenodo.17378284.
(PDF)

S7 Fig GC content trends across 5′ codons in orthologous *E. coli* genes. GC content is averaged at each nucleotide position across orthologs that are at least 180 codons long (*n* ~ 1,400). The *x* axis represents nucleotide positions relative to the start codon (i.e., the third nucleotide of the start codon is labeled as position 0). Error bars indicate the standard error of the mean (SEM). Dashed vertical black line marks the first 10 codons. Locally estimated scatterplot smoothing (LOESS) regression lines are included. The data underlying this Figure can be found in https://doi.org/10.5281/zenodo.17378284.
(PDF)

S8 Fig Comparison of *K*_4_ and *K*_s_ values for alignments with and without indels in the focal lineage alignment. For *E. coli* (**A–G**) and *Bacillus* (**H–N**), we calculate K by codon in one instance assigning codon position by position within the alignment and in the second instance by first removing aligned codons where the focal lineage has an indel. In each group, the first Figure (A, H) is *K*_s_ for the full alignment, the second (B, I) *K*_s_ for the indel removed case with the following figure (C, J) a scatter plot comparing the two with orthogonal regression lines and Pearson correlation. The following sets (D, E, F), (K, L, M) are the same, but for *K*_4_. Plots G and N show the proportion of genes/alignments with an indel at each codon position. The data underlying this Figure can be found in https://doi.org/10.5281/zenodo.17378284.
(PDF)

S9 Fig Distribution of size of 5′ overlaps in *Escherichia coli* native genes. Plot includes all 5′ overlapping genes in the reference genome (669 of 4494 genes, around 15%). The *x* axis represents base pairs (bp) for which each gene is overlapping another one, and the *y* axis the number of genes that overlap by that bp length. Median overlap size is 3 bp. The data underlying this Figure can be found in https://doi.org/10.5281/zenodo.17378284.
(PDF)

S10 Fig Distribution of size of 5′ overlaps in *Bacillus toyonensis* native genes. Plot includes all 5′ overlapping genes in the reference genome (479 of 5229 genes, around 9%). The *x* axis represents base pairs (bp) for which each gene is overlapping another one, and the *y* axis the number of genes that overlap by that bp length. Median overlap size is 3 bp. The data underlying this Figure can be found in https://doi.org/10.5281/zenodo.17378284.
(PDF)

S11 Fig GC content trends across 5′ codons in orthologous *Bacillus toyonensis* genes. GC content is averaged at each nucleotide position across orthologs that are at least 50 codons long (*n* = 2,809). The *x* axis represents nucleotide positions relative to the start codon (i.e., the third nucleotide of the start codon is labeled as position 0). Error bars indicate the standard error of the mean (SEM). Dashed vertical black line marks the first 10 codons. Locally estimated scatterplot smoothing (LOESS) regression lines are included. The data underlying this Figure can be found in https://doi.org/10.5281/zenodo.17378284.
(PDF)

S12 Fig Comparison of codon usage bias and GC content in *E. coli* and *B. subtilis* between operonic genes. For *E. coli*, **A.** is non-operonic genes’ GC3 by position, **B**. the operonic GC3, and **C.** the comparison of the two employing orthogonal regression and Pearson correlation. **D–F** is the same, but for codon usage bias. **G–L** are the same as A–F, but for *B. subtilis*. The data underlying this Figure can be found in https://doi.org/10.5281/zenodo.17378284.
(PDF)

S13 Fig Trends in GC3 content and codon usage bias for four species of bacteria: A and B for *Mycobacterium tuberculosis*, C and D for *Synechocystis* sp., E and F for *Bacteroides thetaiotaomicron*, and G and H for *Leptospira interrogans.* Codon optimality scores obtained from Wei and colleagues [74]. The data underlying this Figure can be found in https://doi.org/10.5281/zenodo.17378284.
(PDF)

S14 Fig Deviation in usage of optimal codon trends as a function of distance from the CDS start, by amino acid block, for *Escherichia coli.* Linear regression lines (and respective displayed Pearson correlation and *P*-value) in color consider the first 10 codons (inclusive), those in black are for all other codon positions. Plots with lines, statistics, and titles in pink show those amino acid blocks where the optimal codon is A/T-ending, those in blue have a G/C-ending optimal codon. Optimal codons and degeneracy for each block are indicated in the plot title. Note the 6-fold degenerate amino acids are divided into a 4-fold and a 2-fold block. The data underlying this Figure can be found in https://doi.org/10.5281/zenodo.17378284.
(PDF)

S15 Fig Deviation in usage of optimal codon trends as a function of distance from the CDS start, by amino acid block, for *Bacillus subtilis.* Linear regression lines (and respective displayed Pearson correlation and *P*-value) in color consider the first 10 codons (inclusive), those in black are for all other codon positions. Plots with lines, statistics, and titles in pink show those amino acid blocks where the optimal codon is A/T-ending, those in blue have a G/C-ending optimal codon. Optimal codons and degeneracy for each block are indicated in the plot title. Note the 6-fold degenerate amino acids are divided into a 4-fold and a 2-fold block. The data underlying this Figure can be found in https://doi.org/10.5281/zenodo.17378284.
(PDF)

S16 Fig Mutation profile as a function of distance from the gene’s start. Same as Fig 5, but for *E. coli* non-overlapping genes only. **A.** Mutation density (mutations per kilobase, kb) as a function of within-gene position. The amount of sequence with each genic window, across all CDS, was determined, the density then being the number of mutations per bp, here scaled to kb. The blue line is a polynomial regression of degree 4. Yellow dashed line and yellow statistic are for the first 60 codons, dark purple dashed line and dark purple statistic are for the rest of the gene. Pearson correlation provided. **B.** Comparison of *K*_4_ values by codon and mutation density from WT lines. Mutation density is in blue with positions specified by mid-position of the window. *K*_4_ data per codon is in pink. Lines reflect polynomial regression of degree 4. To determine pseudo-significance, we interpolate values for each codon by fitting to the blue polynomial line. These values are then correlated against the observed *K*_4_ values (Pearson correlation shown). **C.** Deviation from null (*O* − *E*)/*E* for WT (alternative metric for data in panel A) and from MA lines that have MMR deleted. The first 60 codons are positively correlated for the WT data (statistics as panel A), but the MMR deletion data is not (Pearson correlation *r* = 0.78, *P*-value = 0.06). Dark purple dashed line is regression for data post-60 codons for MMR-deficient data, yellow dashed line for data within 60 codons. The pink line is the polynomial regression for MMR-deficient the blue for WT. The horizontal gray line marks (*O* − *E*)/*E* = 0. In all panels mutational data from Wei and colleagues [56]. The data underlying this Figure can be found in https://doi.org/10.5281/zenodo.17378284.
(PDF)

S17 Fig Mutation profile as a function of distance from the gene’s start. Same as Fig 5, but for WT *E. coli* mutational data from Zhang and colleagues [57] for high-depth samples (**A–C**), and for low-depth samples (**D–F**), and for *E. coli* mutational data from Foster and colleagues [58] (**G–I**). In all, panels yellow dashed lines and yellow statistic consider the first 50 codons, while dark purple considers the rest of the gene (unlike the codon 60 threshold set in Fig 5). The data underlying this Figure can be found in https://doi.org/10.5281/zenodo.17378284.
(PDF)

S18 Fig Trinucleotide centered mutability in genic and intergenic sequence. Observed trinucleotide mutation frequencies in **A.** genic regions and **B.** intergenic regions. Mutation frequency refers to mutation count per occurrence of ancestor base. Trinucleotide mutations are such that the middle base is the mutated base. Trinucleotides on the *x* axis are rank-ordered from most to least frequent. Mutation data for *E. coli* from Wei and colleagues [56] WT samples. **C.** The same trinucleotide frequencies in A and B, plotted against each other. Pink is a linear regression line, Pearson correlation, and respective *P*-value displayed. Dark gray line is a regression line with slope 1 and intercept 0 (i.e., perfect correlation between the two regions). Note if the pink line sits above the perfect correlation line, it represents higher trinucleotide mutation frequencies in intergenic regions. **D–E.** The same trinucleotide frequencies in A–B, plotted against trinucleotide GC content for genic and intergenic trends, respectively. Pearson correlation and respective *P*-value displayed. **F–J** same as A–E, but for MMR-deficient Wei and colleagues [56] samples. **K–O** same as A–E, but for mutation data for *Escherichia coli* from Zhang and colleagues [57] for samples sequences at higher depth. **P–T** same as K–O, but for samples sequenced at lower depth. The data underlying this Figure can be found in https://doi.org/10.5281/zenodo.17378284.
(PDF)

S19 Fig Mutational rates by codon position expected by genomic mutational rates. Mutation refers to mononucleotide changes. Position on the *x* axis refers to codons. Dashed vertical black line marks the first 10 codons. For **A–C**, mutation data for *E. coli* from Wei and colleagues [56], and for **D–F** mutational data for *E. coli* from Zhang and colleagues [57] samples sequenced at higher depth. The data underlying this Figure can be found in https://doi.org/10.5281/zenodo.17378284.
(PDF)

S20 Fig The influence of trinucleotide context on mutation and substitution. **A.** Observed genomic trinucleotide mutational frequencies rank-ordered from most to least frequent in *Escherichia coli*. Mutational data for *E. coli* from Zhang and colleagues [57] samples sequenced at higher depth. Mutation frequency refers to mutation count per occurrence of ancestor base. Trinucleotide mutations are such that the middle base is the mutated base. Trinucleotides on the *x* axis are rank-ordered by frequency and bars are color-coded by trinucleotide GC content. **B.** The same genomic mutation frequencies as in A, plotted against trinucleotide GC content. Line represents linear regression and Pearson correlation with respective *P*-value is also shown. **C.** Expected trinucleotide mutation rates by codon position, predicted by trinucleotide genomic mutational rates and genomic trinucleotide content. Trinucleotide mutations are such that the middle base is the mutated base, and it occurs at third sites in 4-fold degenerate codons. **D.** Comparison of expected mutational rates in C with *E. coli* conservation trends by codon position at 4-fold degenerate sites (4-fold *K*_s_, as seen in S5 Fig). Both metrics are normalized by Z score. **E**. Comparison of expected trinucleotide mutation rates and *K*_4_ by position without Z transformation. Pearson correlation data is shown. Line is the orthogonal (major axes) regression line. **F–J** as A–E, but for *E. coli* from Zhang and colleagues [57] samples sequenced at lower depth. For panels C, D, H, and I, position on the *x* axis refers to absolute number of codons (where the start codon is position 1), and the dashed vertical black line marks the first 10 codons. Locally estimated scatterplot smoothing (LOESS) regression lines are also provided. The data underlying this Figure can be found in https://doi.org/10.5281/zenodo.17378284.
(PDF)

S21 Fig Trinucleotide mutational frequencies at 5′ ends and gene cores. **A**. Comparison between each possible trinucleotide mutation in the two gene regions. Points are color-coded by ancestral base. **B.** Trinucleotide mutational frequencies at 5′ ends rank ordered from largest to smallest. **C.** Trinucleotide mutational frequencies in gene cores rank-ordered from largest to smallest. Bars in B and C are color-coded by GC content within the trinucleotide. For all plots, trinucleotide mutations are such that the middle base is the mutated base. For A–C mutational data from Wei and colleagues [56], and **D–F** are the same but with mutation data from Zhang and colleagues [57]. For all panels 5′ ends include the first 20 codons, while gene cores refer to the rest of the CDS. The data underlying this Figure can be found in https://doi.org/10.5281/zenodo.17378284.
(PDF)

S22 Fig Mutation profile as a function of distance from the gene’s start for *Mycobacterium smegmatis.* **A.** Mutation density (mutations per kilobase, kb) as a function of within gene position. The amount of sequence with each genic window, across all CDS, was determined, the density then being the number of mutations per bp, here scaled to kb. The blue line is a polynomial regression of degree 4. Yellow dashed line and yellow statistic are for the first 60 codons, dark purple dashed line and dark purple statistic are for the rest of the gene. Pearson correlation provided. **B.** Comparison of *K*_4_ values by codon and mutation density from WT lines. Mutation density is in blue with positions specified by mid-position of the window. *K*_4_ data per codon is in pink. Lines reflect polynomial regression of degree 4. To determine pseudo-significance, we interpolate values for each codon by fitting to the blue polynomial line. These values are then correlated against the observed *K*_4_ values (Pearson correlation shown). **C.** Deviation from null (*O* − *E*)/*E* for WT (alternative metric for data in panel A) and from MA lines that have MMR deleted. The first 60 codons are positively correlated for the WT data (statistics as panel A), but the MMR deletion data is not (Pearson correlation *r* = 0.78, *P*-value = 0.06). Dark purple dashed line is regression for data post-60 codons for MMR-deficient data, yellow dashed line for data within 60 codons. The pink line is the polynomial regression for MMR-deficient, the blue for WT. The horizontal gray line marks (*O* − *E*)/*E* = 0. In all panels mutational data for WT is combined from Castañeda-Garcia and colleagues [60] and Kucukyildirim and colleagues [61], while data for MMR-deficient samples is from Castañeda-Garcia and colleagues [60] only. The data underlying this Figure can be found in https://doi.org/10.5281/zenodo.17378284.
(PDF)

S23 Fig Substitution rates by 5′ codon position comparing the *Mycobacterium smegmatis-M. goodii* ancestor to *M. smegmatis.* A. Synonymous substitution rates (*K*_s_); **B.** non-synonymous substitution rates (*K*_a_), and **C.** the ratio between the two (*K*_a_/*K*_s_). A–C plots include orthologs that are at least 180 codons in length (*n* = 1,698). For A–C, the *x* axis represents absolute codon position (i.e., the start codon is codon 1). Dashed vertical black line marks the first 10 codons. Locally estimated scatterplot smoothing (LOESS) regression lines are included. Note that codon position here is by reference to the codon position in the alignment. **D–I** Comparison of *K*_4_ and *K*_s_ values for alignments with and without indels in the focal lineage alignment. We calculate *K* by codon in one instance assigning codon position by position within the alignment and in the second instance by first removing aligned codons where the focal lineage has an indel. D is *K*_s_ for the full alignment, E *K*_s_ for the indel removed case, and F a scatter plot comparing the two with orthogonal regression lines and Pearson correlation. The following set (G, H, I) are the same but for *K*_4_. Note removal of alignment indels in the focal lineage prior to codon position categorization makes no meaningful difference. **J.** Proportion of genes/alignments with an indel at each codon position. The data underlying this Figure can be found in https://doi.org/10.5281/zenodo.17378284.
(PDF)

S1 Table Spearman correlation (top panel) and partial correlation (bottom panel) between transgene protein per RNA levels and GC content for each codon position. Transgene data from Cambray and colleagues [41]. Partial Spearman correlations are controlling for the influence of all other codon positions in the available construct sequence (codons 2–30) such that, for instance, the relation between transgene expression (as protein/RNA) and GC content at codon 2 is found independently of the relation with GC content in codons 3–30. Stars represent *P*-value (*p* < 0.0001 = **** *p* < 0.001 = *** *p* < 0.01 = ** *p* < 0.05 = * *p* > 0.05 = no stars); “protein_by_RNA” refers to PNI/RNAss measures reported by Cambray and colleagues. Codon positions refer to absolute codon numbers (e.g., the start codon is codon 1). The data underlying this Figure can be found in https://doi.org/10.5281/zenodo.17378284.
(CSV)

S2 Table Spearman and partial Spearman correlations between ViennaRNA stability predictions at different gene regions and transgene protein/RNA levels. Transgene data from Cambray and colleagues [41]. “stability_first10” refers to predicted stability in the first 30 bp in each transgene construct after the start codon (codons 2–11), “stability_mid” to predicted stability of the following 30 bp section within each transgene construct (codons 12–21), “stability_last10” to the 30 bp following that (codons 22–31). Partial Spearman correlations are controlling for the influence of stability in the other construct regions such that, for instance, the relation between transgene expression (as protein/RNA) and predicted RNA stability in region stability_first10 is found independently of the relation with predicted RNA stability in regions stability_mid and stability_last10. Number of start indicates significance such that *p* <0 .0001 = “****”, *p* < 0.001 = “***”, *p* < 0.01 = “**”, *p* < 0.05 = “*”, *p* > 0.05 = no stars. The data underlying this Figure can be found in https://doi.org/10.5281/zenodo.17378284.
(CSV)

S3 Table Estimated AT content at mutational equilibrium determined by full method. Method involves consideration of all possible mutational classes and solving the relevant simultaneous equations, see Methods. “seq_depth” differentiates the samples into those sequenced with higher or lower depth by Zhang and colleagues [57]. “mut_size” refers to the approach by which the mutational matrix was generated, i.e., counting mononucleotide or dinucleotide mutations. “region” refers to 5′ ends (i.e., the first 20 codons following the start codon), gene cores (i.e., the rest of the CDS), intergenic (i.e., non-CDS mutations), or genomic (i.e., the whole genome including non-protein coding sequences). “pred_AT” is the predicted AT content at mutational equilibrium. “mean_boot_ATestimate” and “std” are the mean and standard deviation of repeating the nucleotide content estimate calculation (i.e., “pred_AT”) for 1,000 bootstraps. The data underlying this Figure can be found in https://doi.org/10.5281/zenodo.17378284.
(CSV)

S4 Table List of bacteria species used for the 5′ versus core GC3 cross-species analysis. “assembly_accession” refers to the accession number for sequence retrieval from RefSeq NCBI [109].
(CSV)

S1 Text Amino acid usage at the 5′ ends of genes forces out GC rich runs. (PDF)

S2 Text Why are mutational equilibrium estimates so diverse? (PDF)

## Abbreviations

CAI: Codon Adaptation Index
CDS: coding sequence
HEGs: highly expressed genes
LOESS: locally estimated scatterplot smoothing
MA: mutation accumulation
MMR: mismatch repair
SEM: standard error of the mean
WT: wild-type
