# Supplementary figures and images for: Bacterial gene 5′ ends have unusual mutation rates that can mislead tests of selection
Source: PLoS Biol. 2025 Dec 15;23(12):e3003569. doi: 10.1371/journal.pbio.3003569 (PMC12725619; doi:10.1371/journal.pbio.3003569)

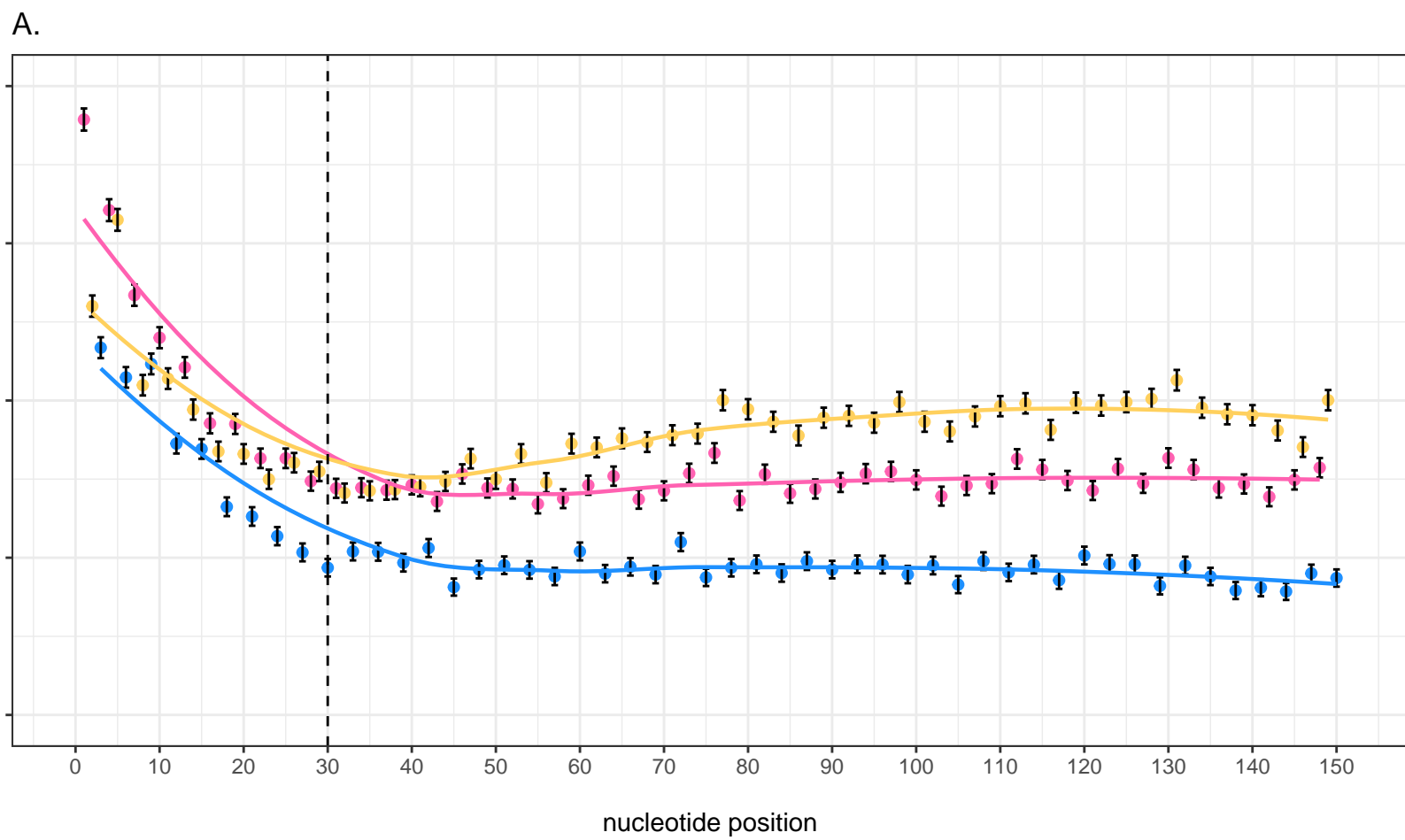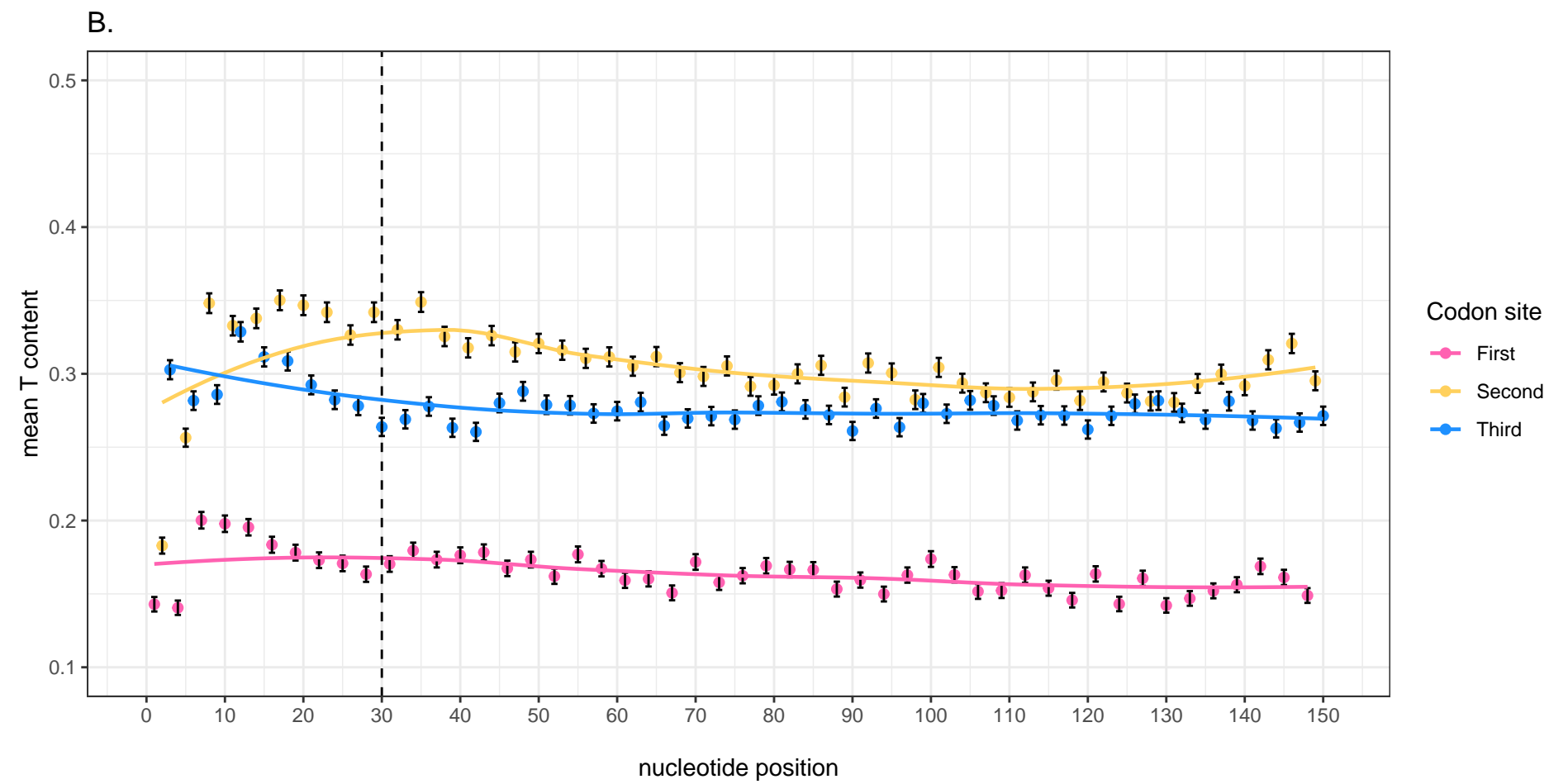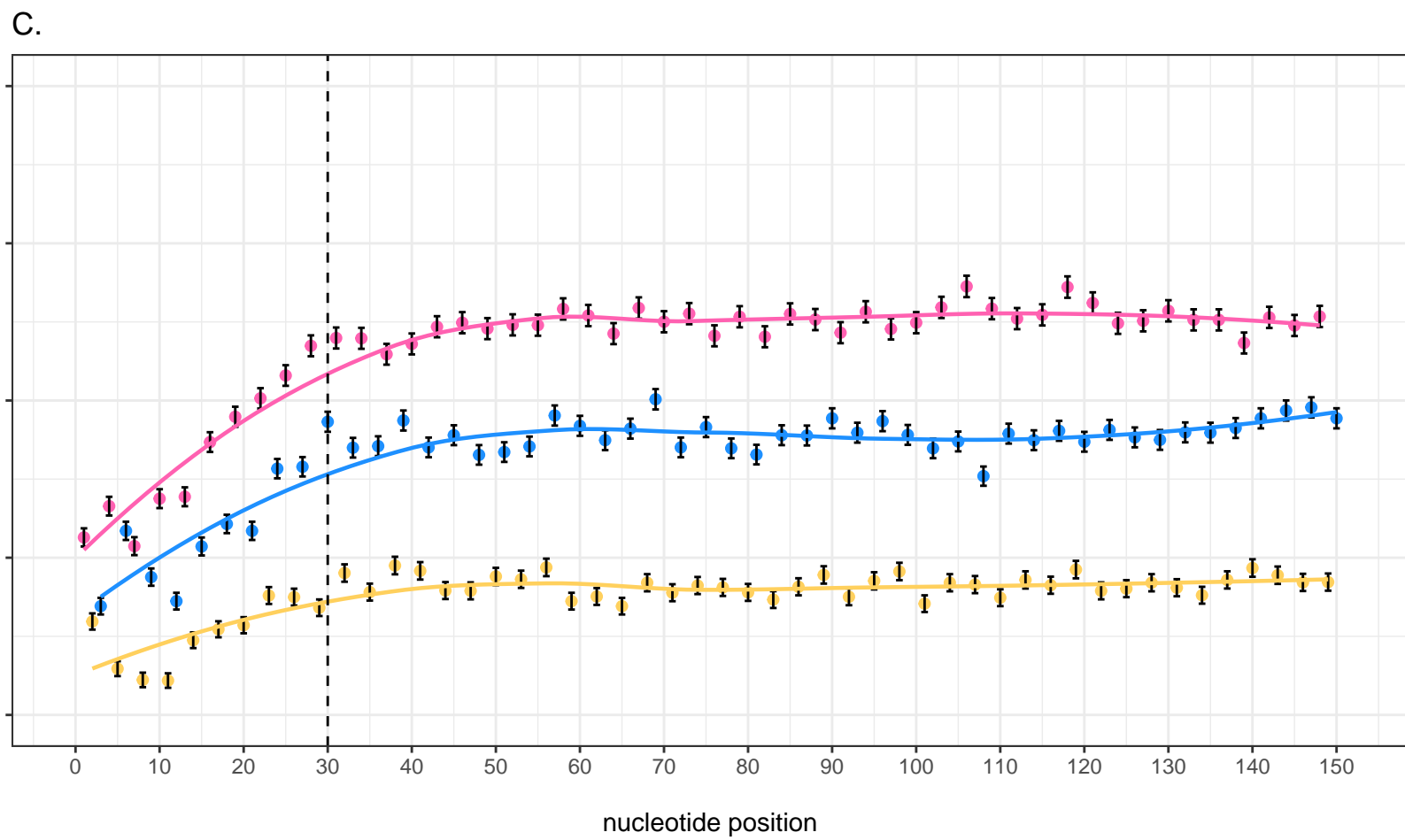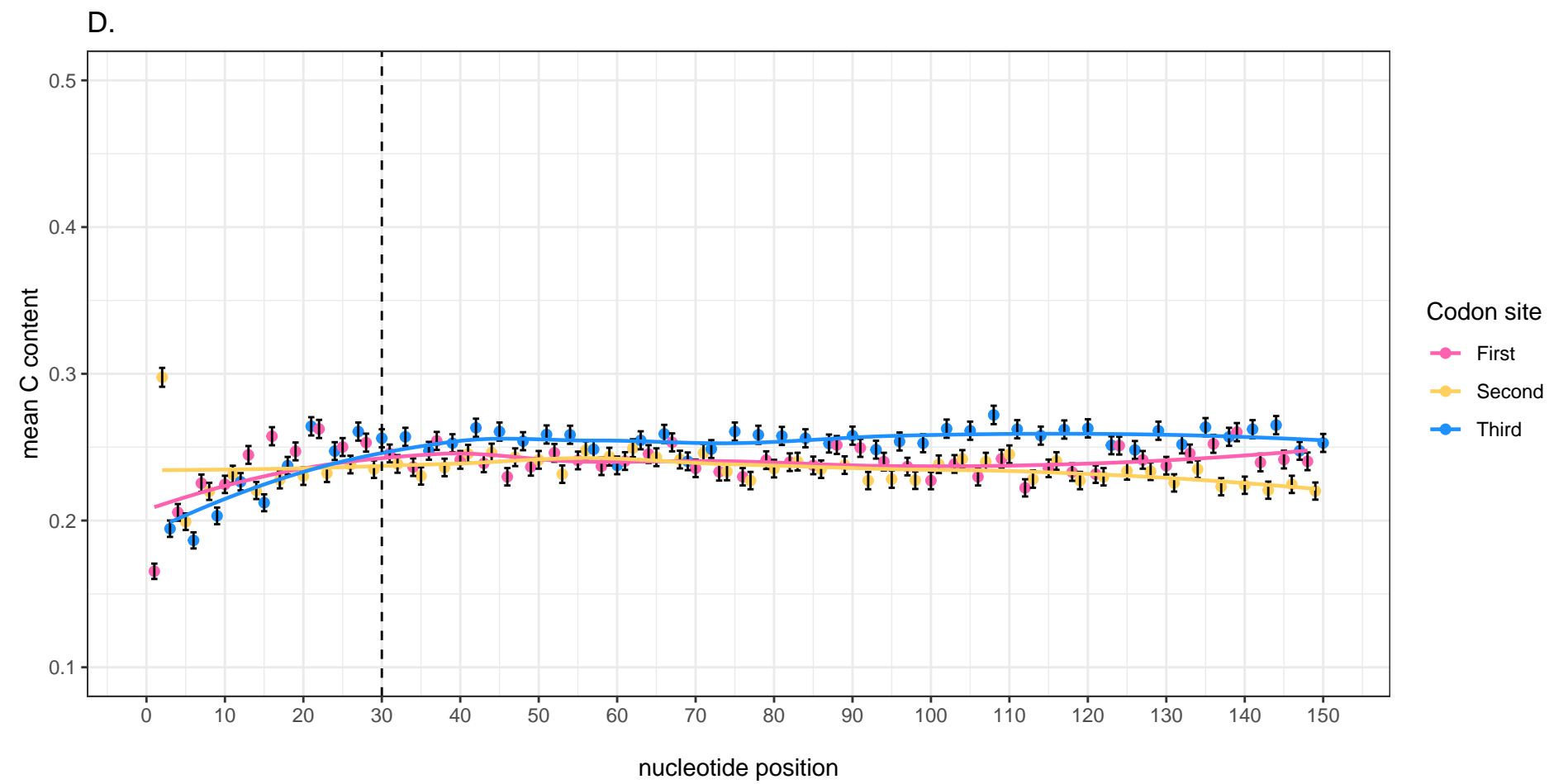

Supplement: S1 Fig — For all four nucleotides, content is averaged at each nucleotide position across 5,098 native genes. The x axis represents nucleotide positions relative to the start codon (i.e., the third nucleotide of the start codon is labeled as position 0). Error bars indicate the standard error of the mean (SEM). Dashed vertical black line marks the first 10 codons. Locally estimated scatterplot smoothing (LOESS) regression lines are included. The data underlying this Figure can be found in https://doi.org/10.5281/zenodo.17378284. (PDF) [file pbio.3003569.s001.pdf]

A.

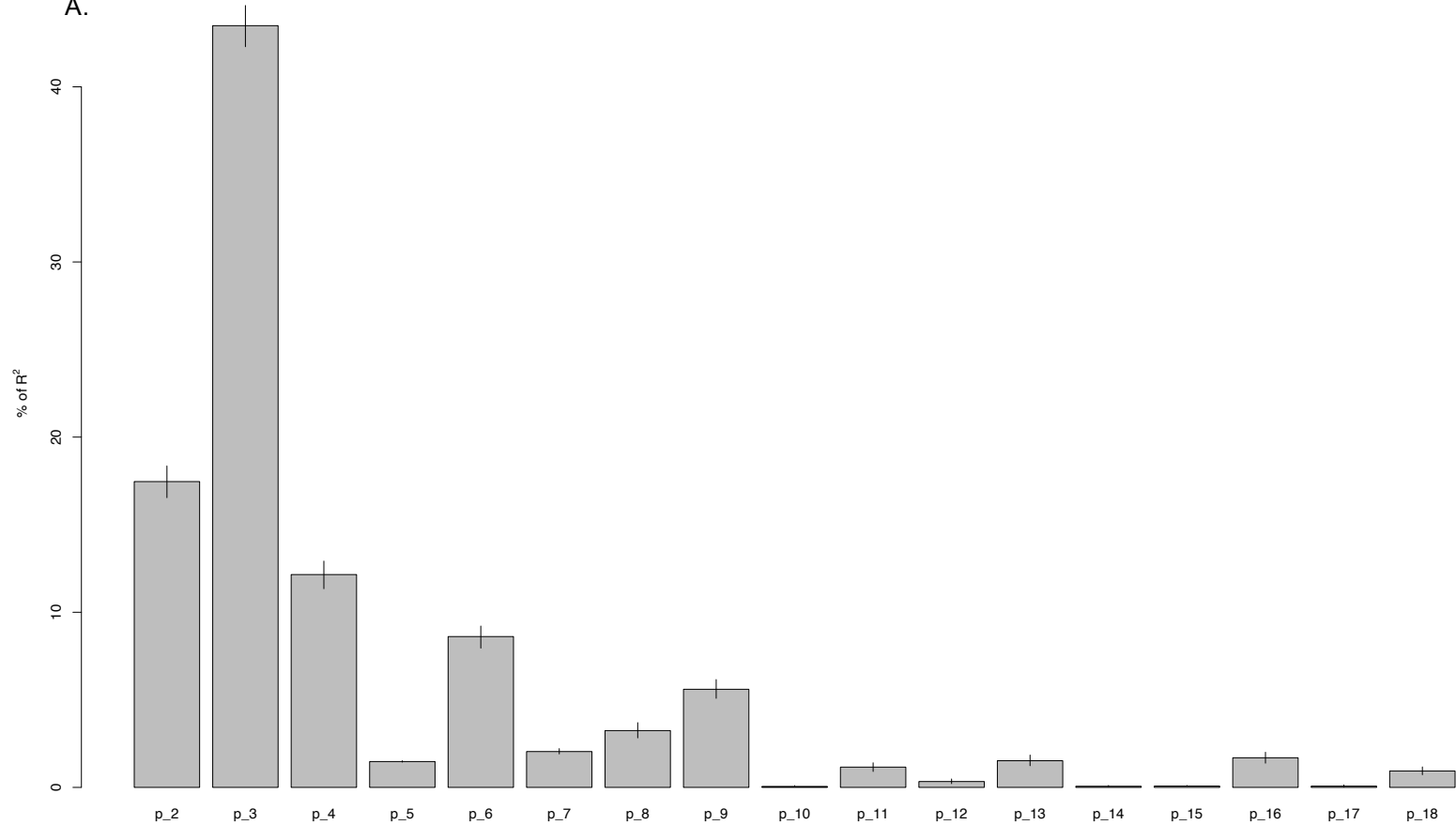

B.

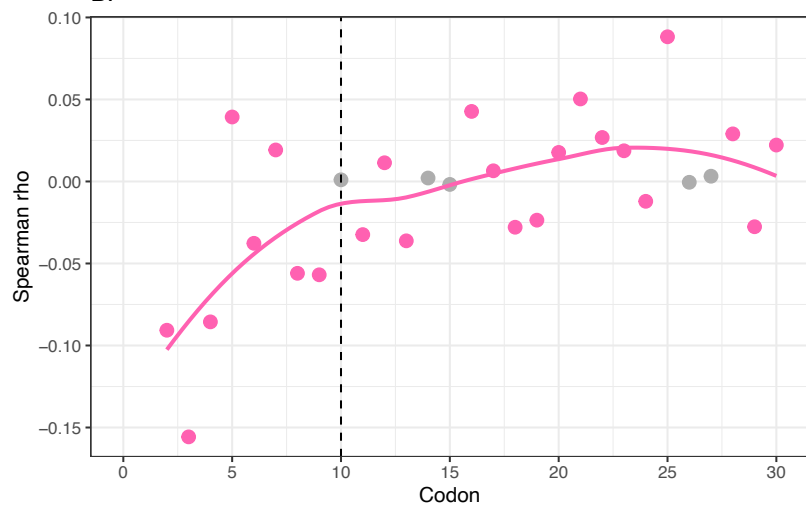

C.

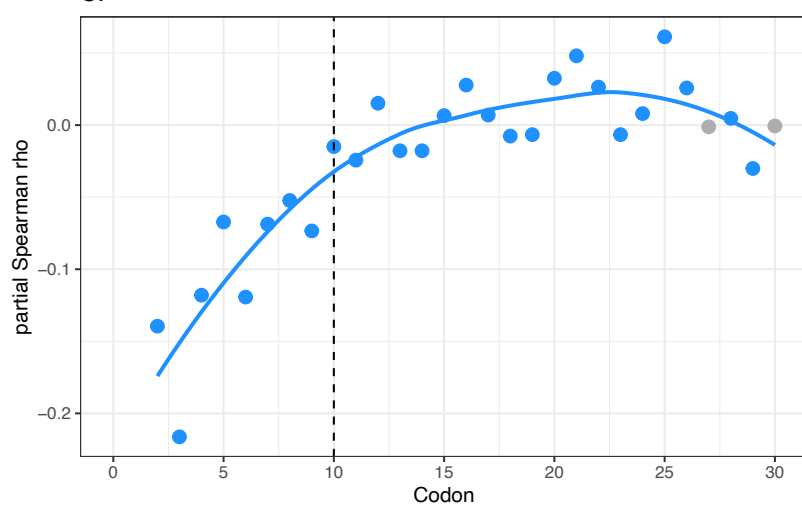

Supplement: S2 Fig — As with in-text Fig 1D–1F, except that the metric is protein level not protein per RNA. A. Relaimpo analysis. The model explains 7.4% of the variation in protein level. B. Spearman correlation analysis. C. partial Spearman correlation. The data underlying this Figure can be found in https://doi.org/10.5281/zenodo.17378284. (PDF) [file pbio.3003569.s002.pdf]

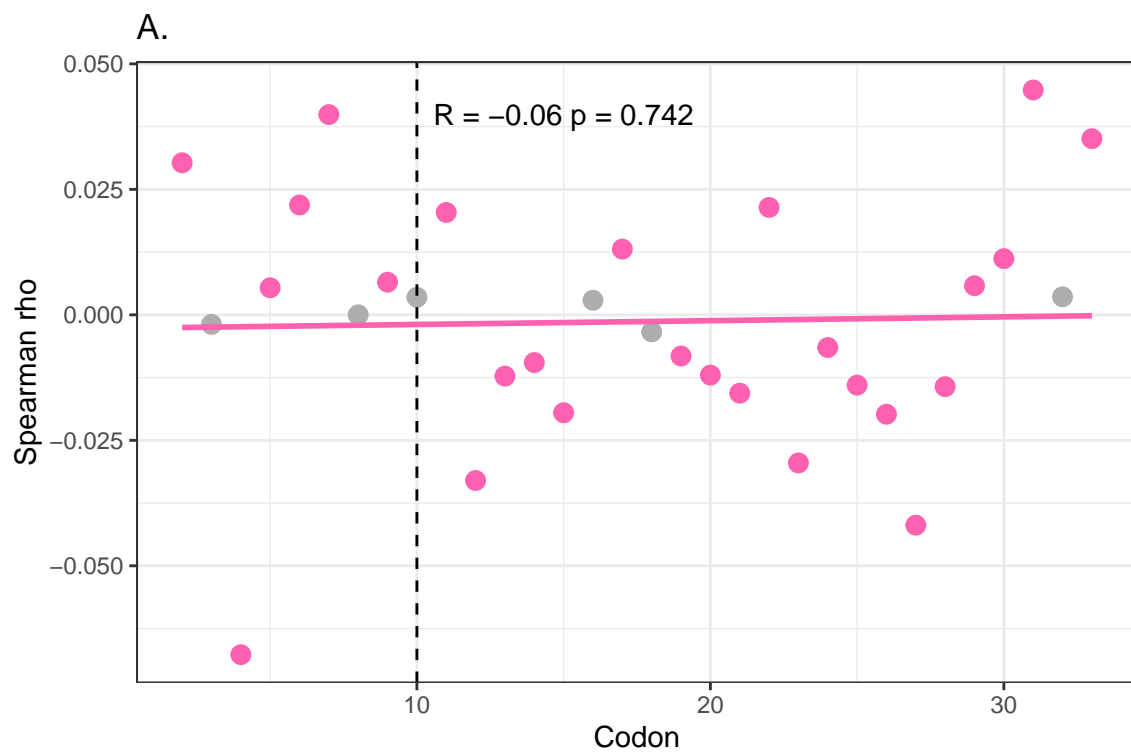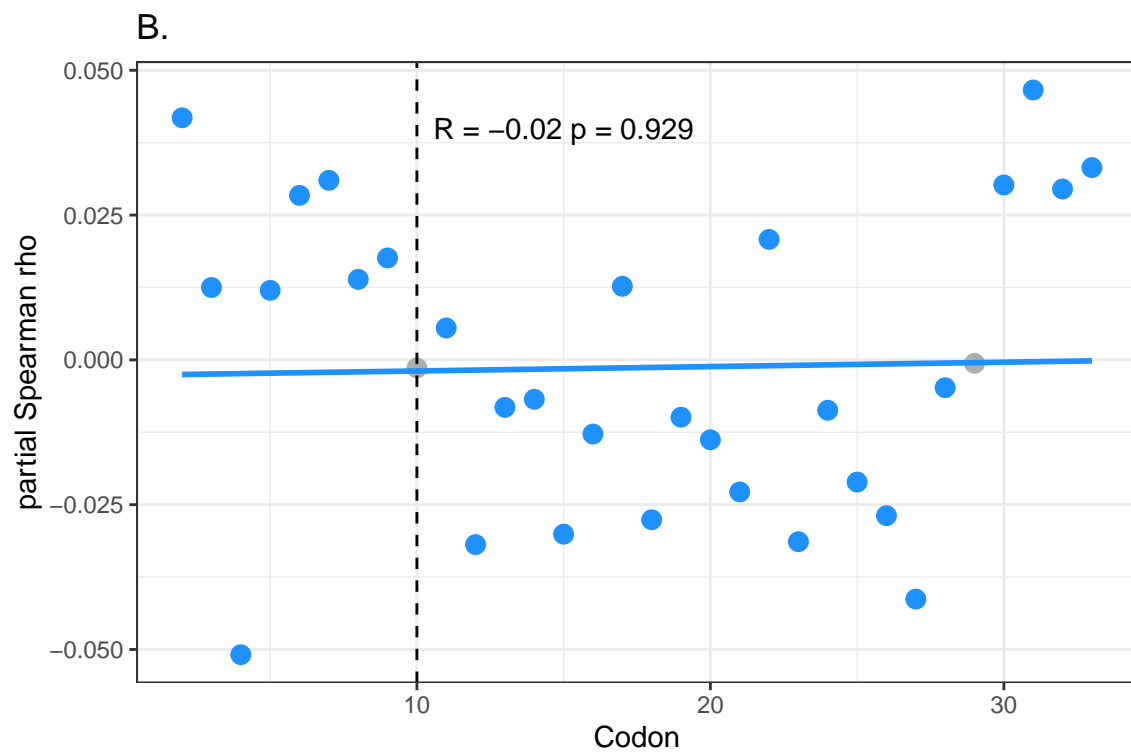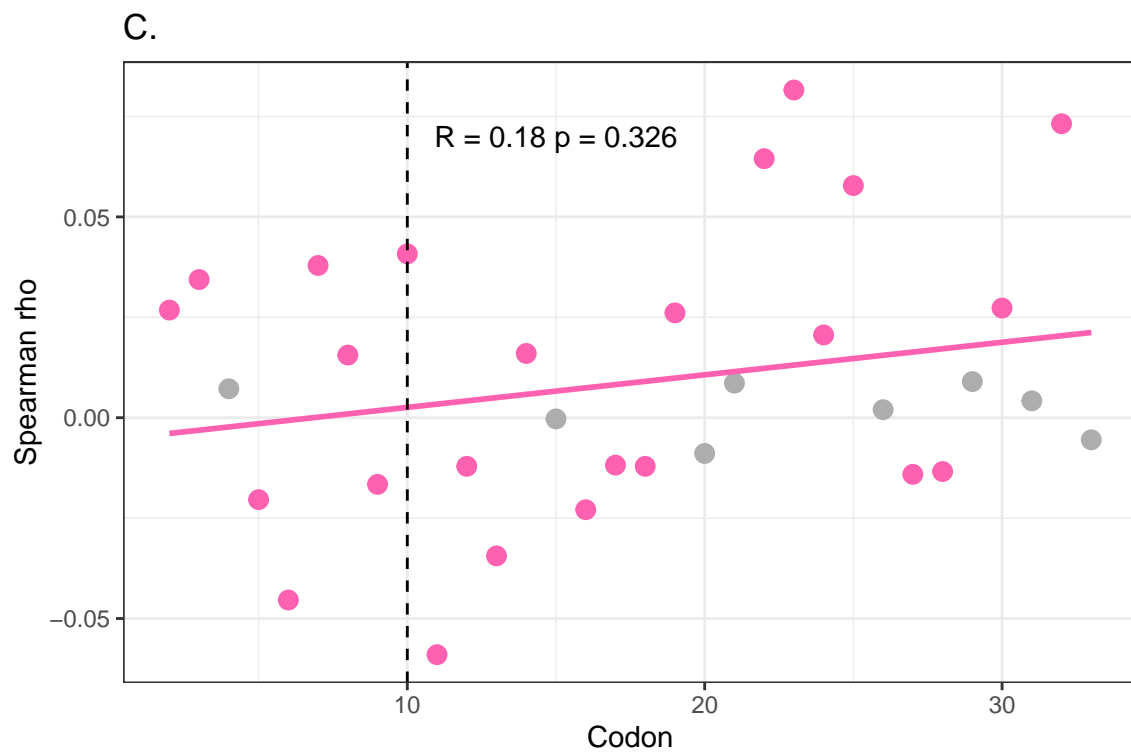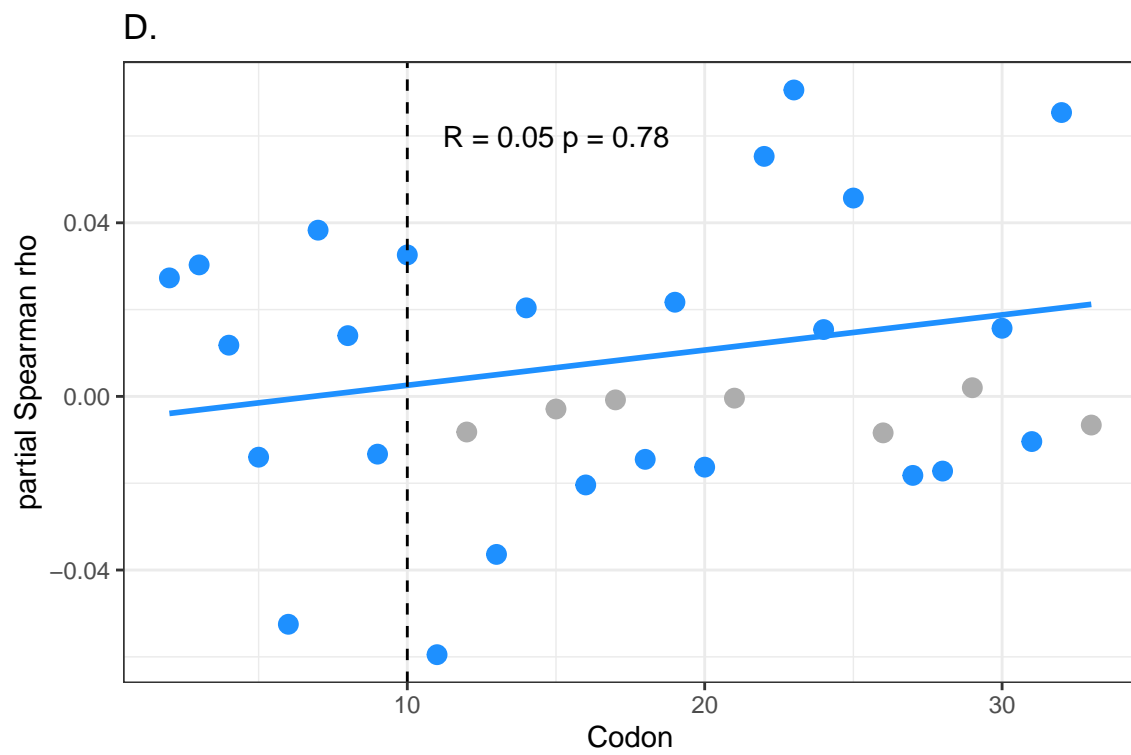

Supplement: S3 Fig — A. Full Spearman correlations for GC content. B. partial Spearman correlations for GC content controlling for the influence of all other codon positions in the available construct sequence (codons 2–30) such that, for instance, the relation between transgene expression and GC content at codon 2 is found independently of the relation with GC content in codons 3–30. C, D same as A, B, but for Codon Adaptation Index (CAI) at each position. Here, CAI measures the enrichment of a codon relative to its synonyms in the 10 codons at genic cores in highly compared to lowly expressed native genes (nHEGs v nLEGs) according to amassed protein abundance data. CAI is represented through log odds ratios such that a higher value means higher usage in nHEGs. In all plots, colored points represent rho values with a P-value ≤ 0.05, while gray points are non-significant. Locally estimated scatterplot smoothing (LOESS) regression lines are included. Codon positions on the x axis refer to absolute codon numbers (e.g., the start codon is codon 1). Dashed vertical black line marks the first 10 codons. Transgene data from Cambray and colleagues [41]. The data underlying this Figure can be found in https://doi.org/10.5281/zenodo.17378284. (PDF) [file pbio.3003569.s003.pdf]

A.

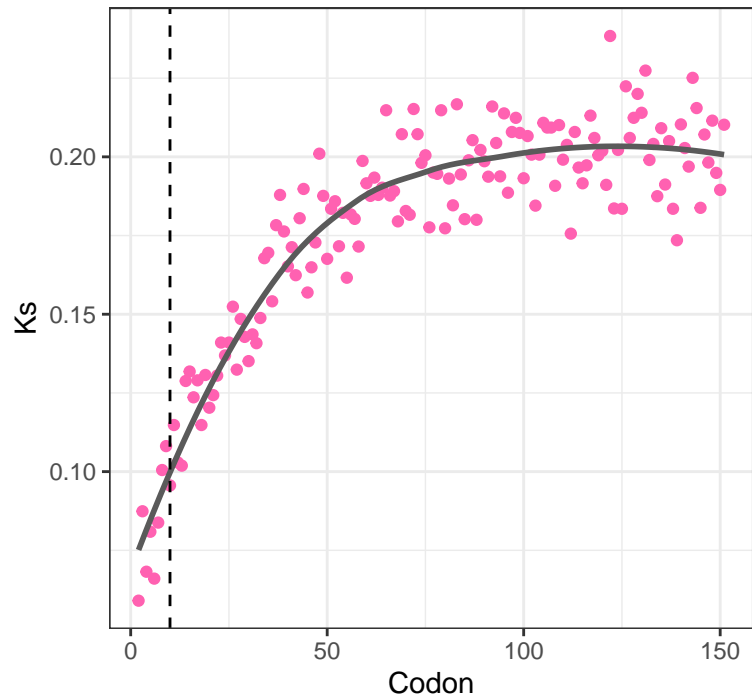

B.

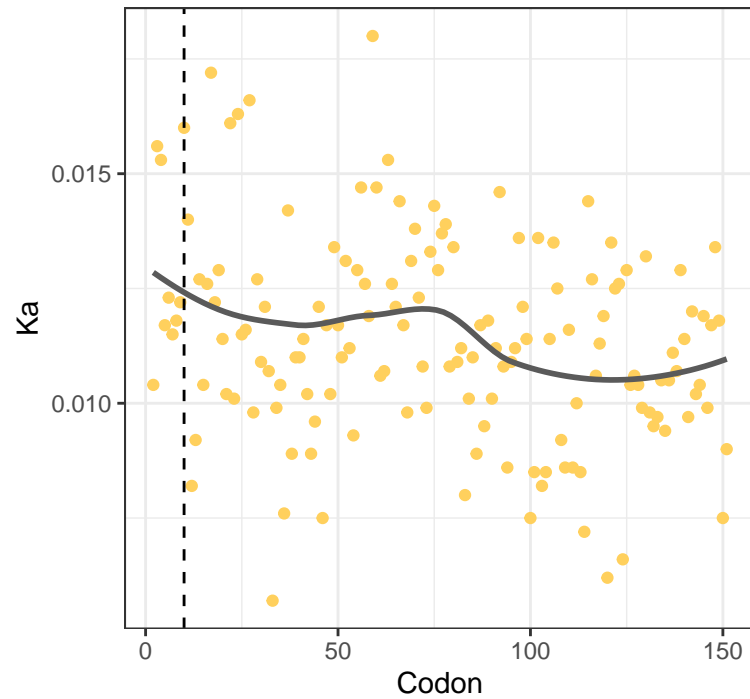

C.

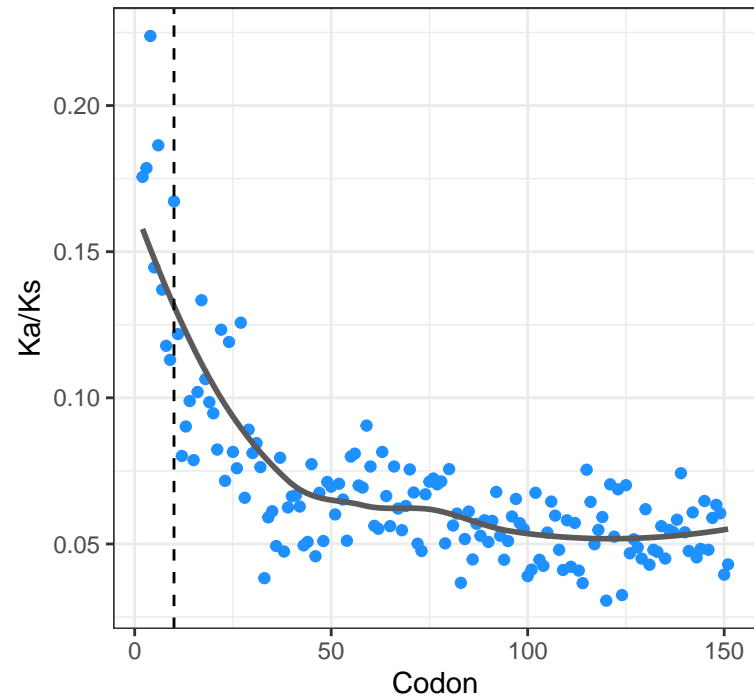

Supplement: S4 Fig — Synonymous substitution rates (Ks); B. non-synonymous substitution rates (Ka), and C. the ratio between the two (Ka/Ks). The x axis represents absolute codon position (i.e., the start codon is codon 1). A–C plots include orthologs that are at least 180 codons long (n ~ 1,400). Dashed vertical black line marks the first 10 codons. Locally estimated scatterplot smoothing (LOESS) regression lines are included. The data underlying this Figure can be found in https://doi.org/10.5281/zenodo.17378284. (PDF) [file pbio.3003569.s004.pdf]

A.

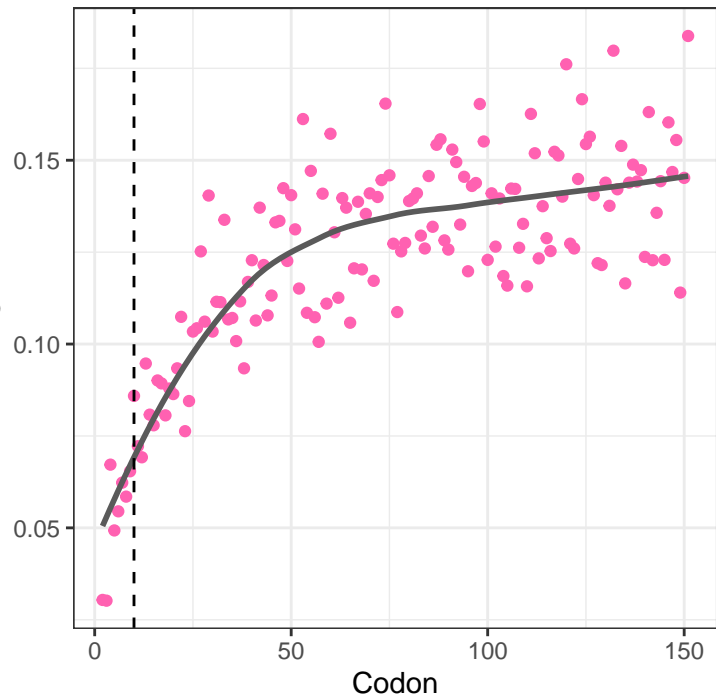

B.

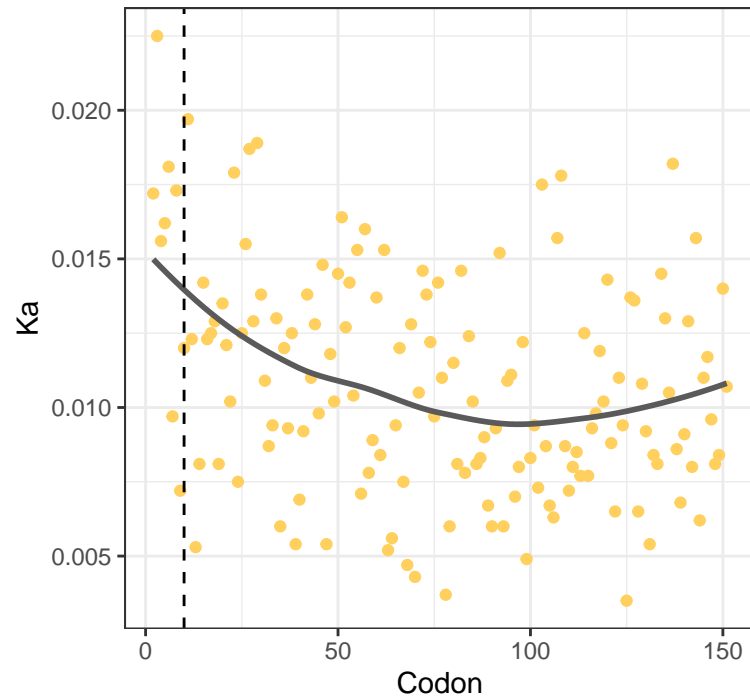

C.

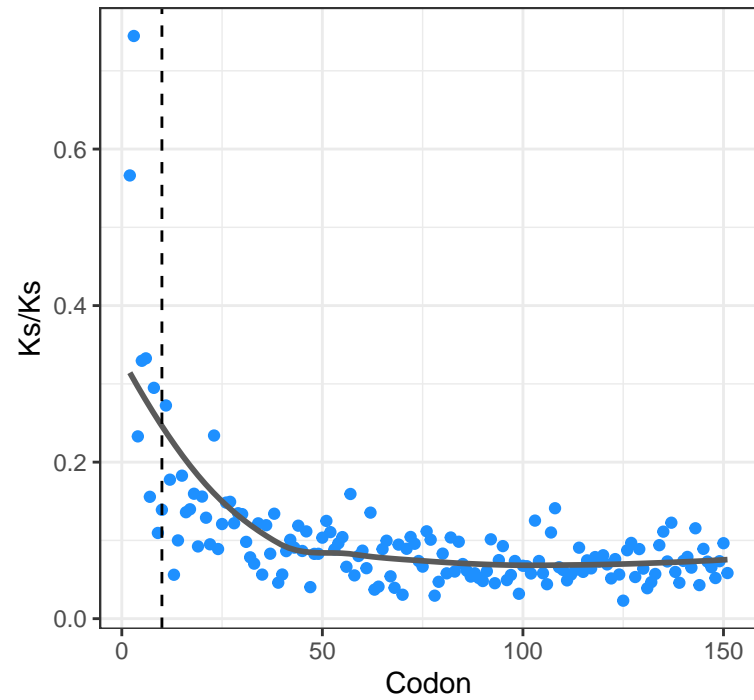

Supplement: S5 Fig — Synonymous substitution rates (Ks); B. non-synonymous substitution rates (Ka), and C. the ratio between the two (Ka/Ks). The x axis represents absolute codon position (i.e., the start codon is codon 1). Dashed vertical black line marks the first 10 codons. Locally estimated scatterplot smoothing (LOESS) regression lines are included. The data underlying this be found in https://doi.org/10.5281/zenodo.17378284. (PDF) [file pbio.3003569.s005.pdf]

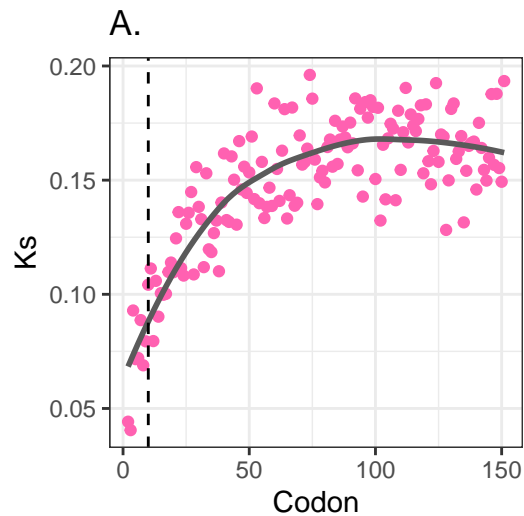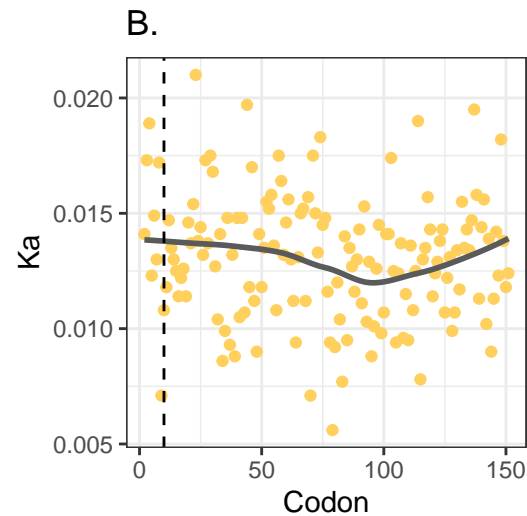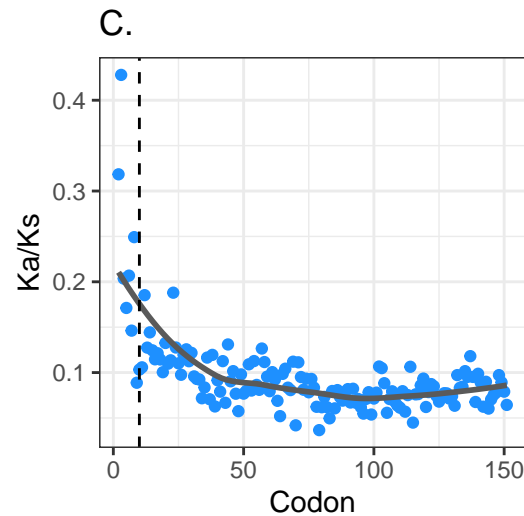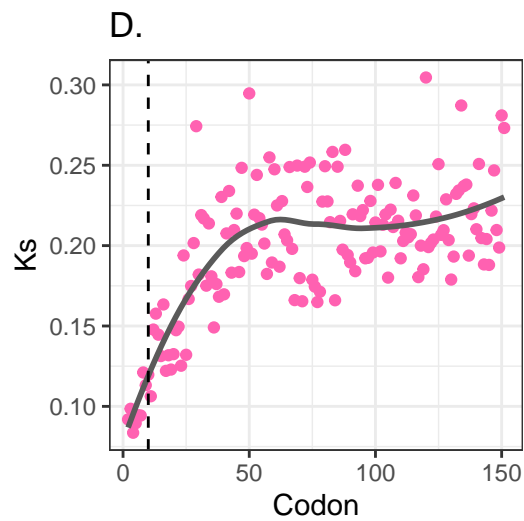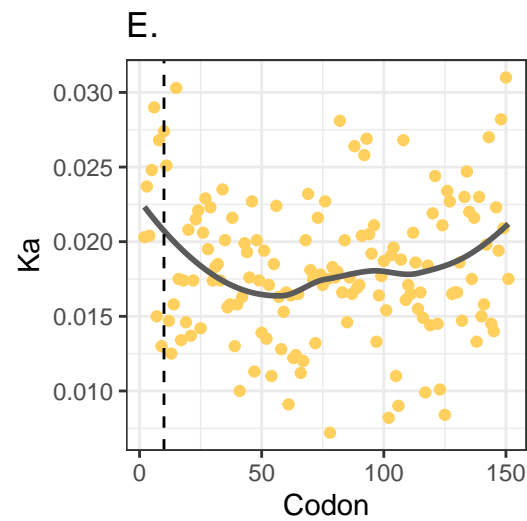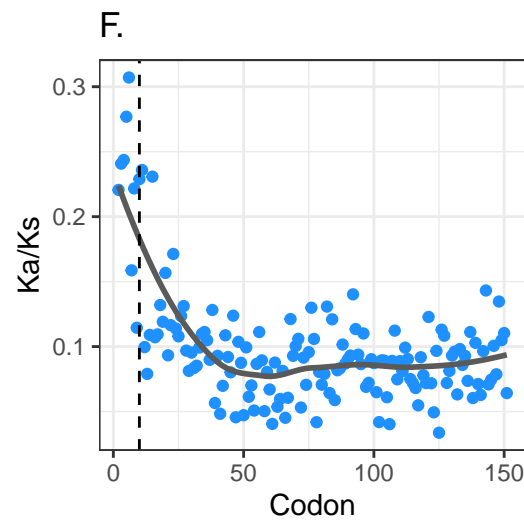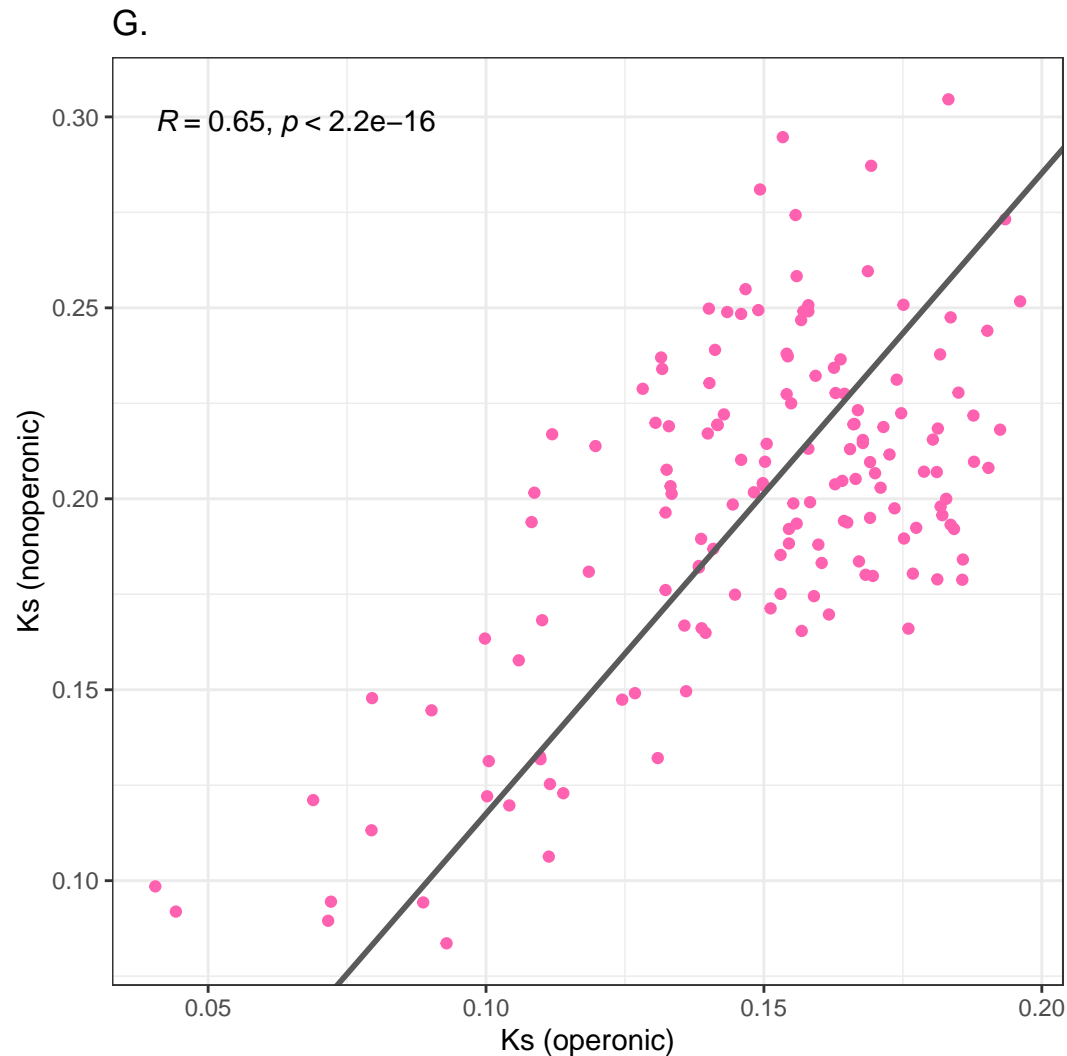

Supplement: S6 Fig — Synonymous substitution rates (Ks); B. non-synonymous substitution rates (Ka), and C. the ratio between the two (Ka/Ks). A–C plots include operonic orthologs that are at least 180 codons in length (n = 1,080). D–F. Same as A–C but only including non-operonic orthologous genes that are at least 180 codons in length (n = 473). For A–F panels, the x axis represents absolute codon position (i.e., the start codon is codon 1). Dashed vertical black line marks the first 10 codons. Locally estimated scatterplot smoothing (LOESS) regression lines are included. Note that codon position here is by reference to the codon position in the alignment. G. The Ks trends seen in operonic (A) and non-operonic (D) genes plotted against each other. Orthogonal regression line and Pearson correlation shown. The data underlying this Figure can be found in https://doi.org/10.5281/zenodo.17378284. (PDF) [file pbio.3003569.s006.pdf]

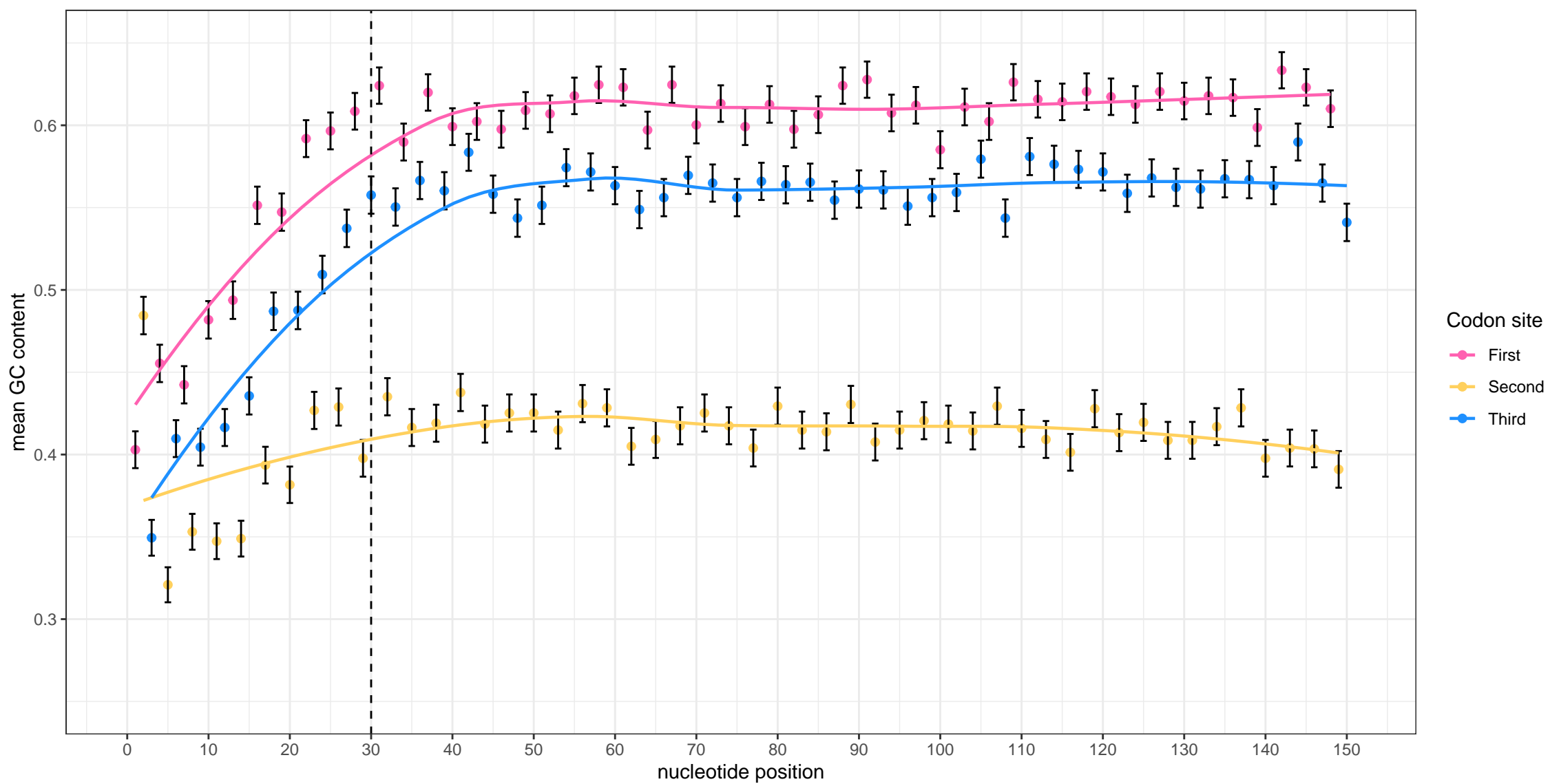

Supplement: S7 Fig — GC content is averaged at each nucleotide position across orthologs that are at least 180 codons long (n ~ 1,400). The x axis represents nucleotide positions relative to the start codon (i.e., the third nucleotide of the start codon is labeled as position 0). Error bars indicate the standard error of the mean (SEM). Dashed vertical black line marks the first 10 codons. Locally estimated scatterplot smoothing (LOESS) regression lines are included. The data underlying this Figure can be found in https://doi.org/10.5281/zenodo.17378284. (PDF) [file pbio.3003569.s007.pdf]

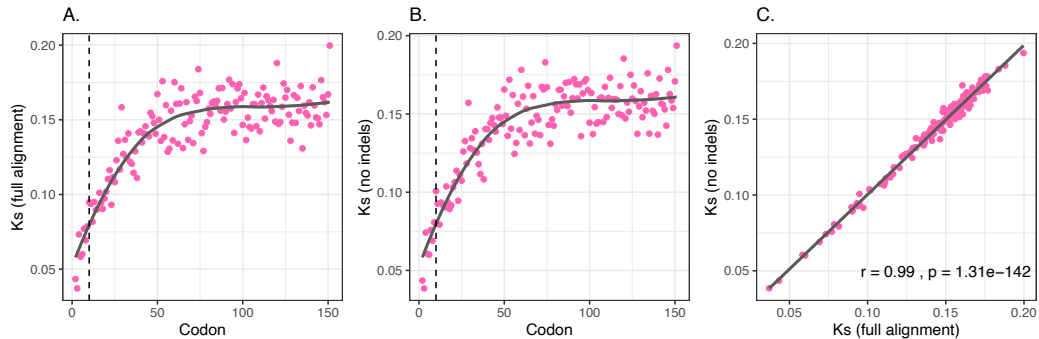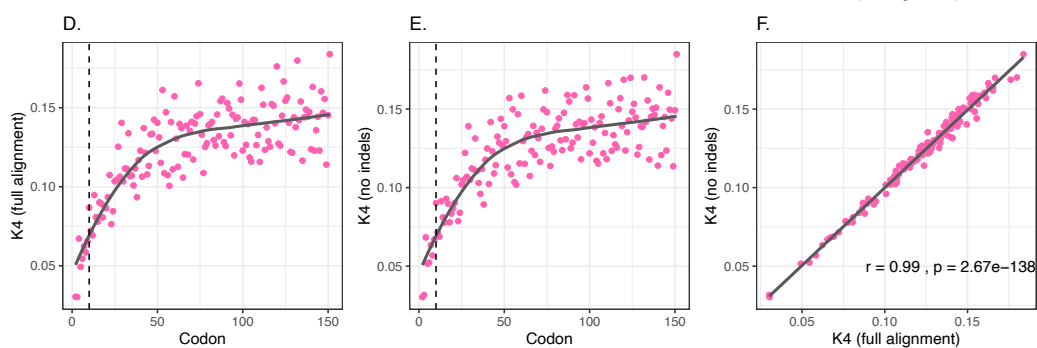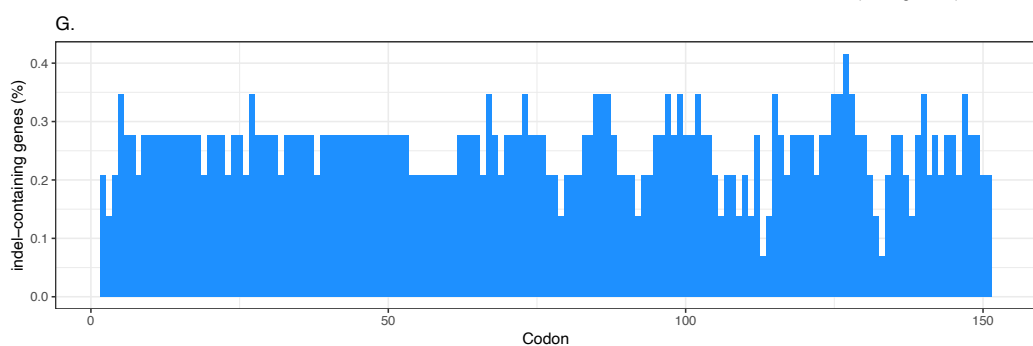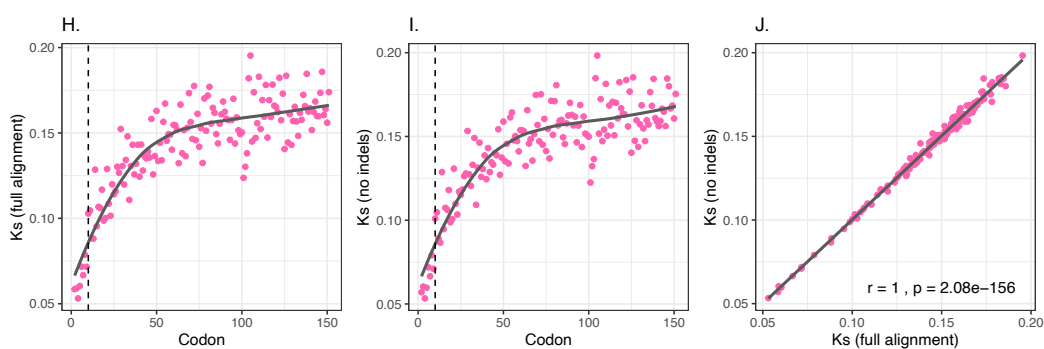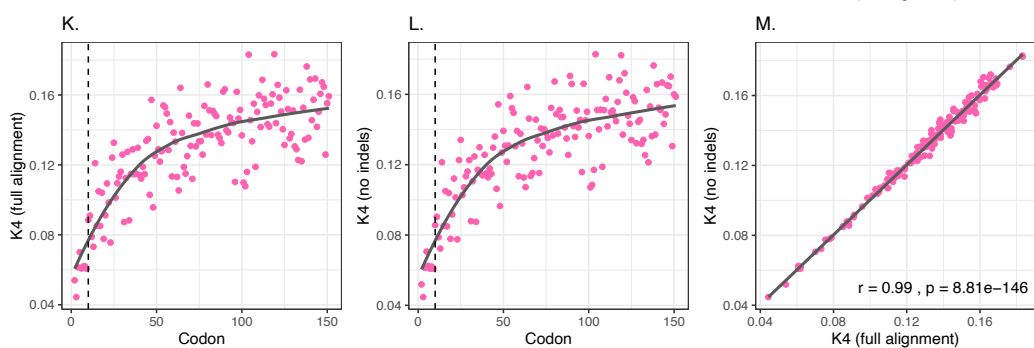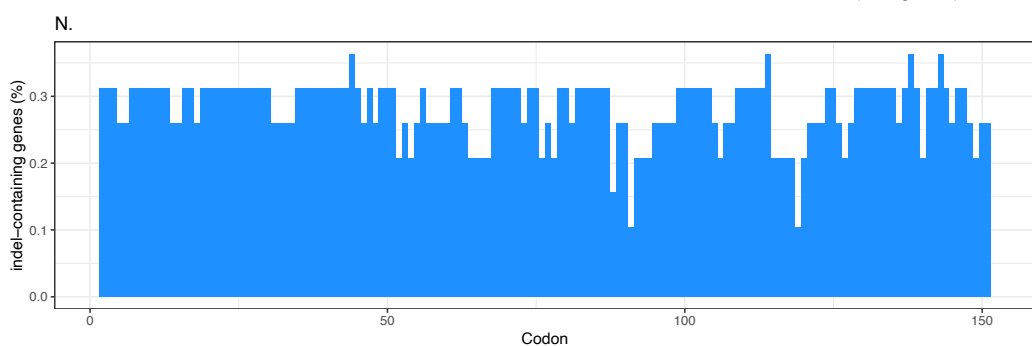

Supplement: S8 Fig — For E. coli (A–G) and Bacillus (H–N), we calculate K by codon in one instance assigning codon position by position within the alignment and in the second instance by first removing aligned codons where the focal lineage has an indel. In each group, the first Figure (A, H) is Ks for the full alignment, the second (B, I) Ks for the indel removed case with the following figure (C, J) a scatter plot comparing the two with orthogonal regression lines and Pearson correlation. The following sets (D, E, F), (K, L, M) are the same, but for K4. Plots G and N show the proportion of genes/alignments with an indel at each codon position. The data underlying this Figure can be found in https://doi.org/10.5281/zenodo.17378284. (PDF) [file pbio.3003569.s008.pdf]

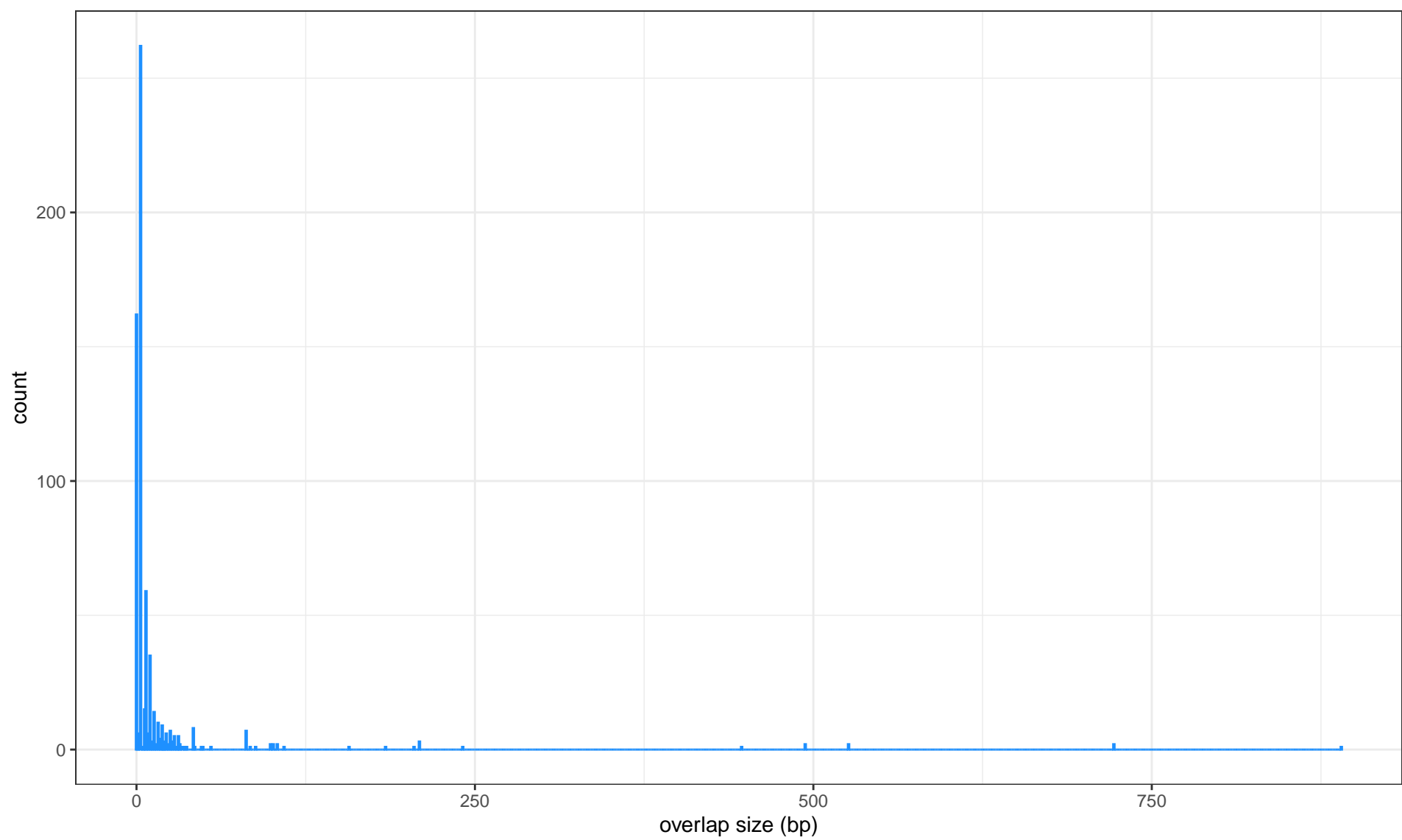

Supplement: S9 Fig — Plot includes all 5′ overlapping genes in the reference genome (669 of 4494 genes, around 15%). The x axis represents base pairs (bp) for which each gene is overlapping another one, and the y axis the number of genes that overlap by that bp length. Median overlap size is 3 bp. The data underlying this Figure can be found in https://doi.org/10.5281/zenodo.17378284. (PDF) [file pbio.3003569.s009.pdf]

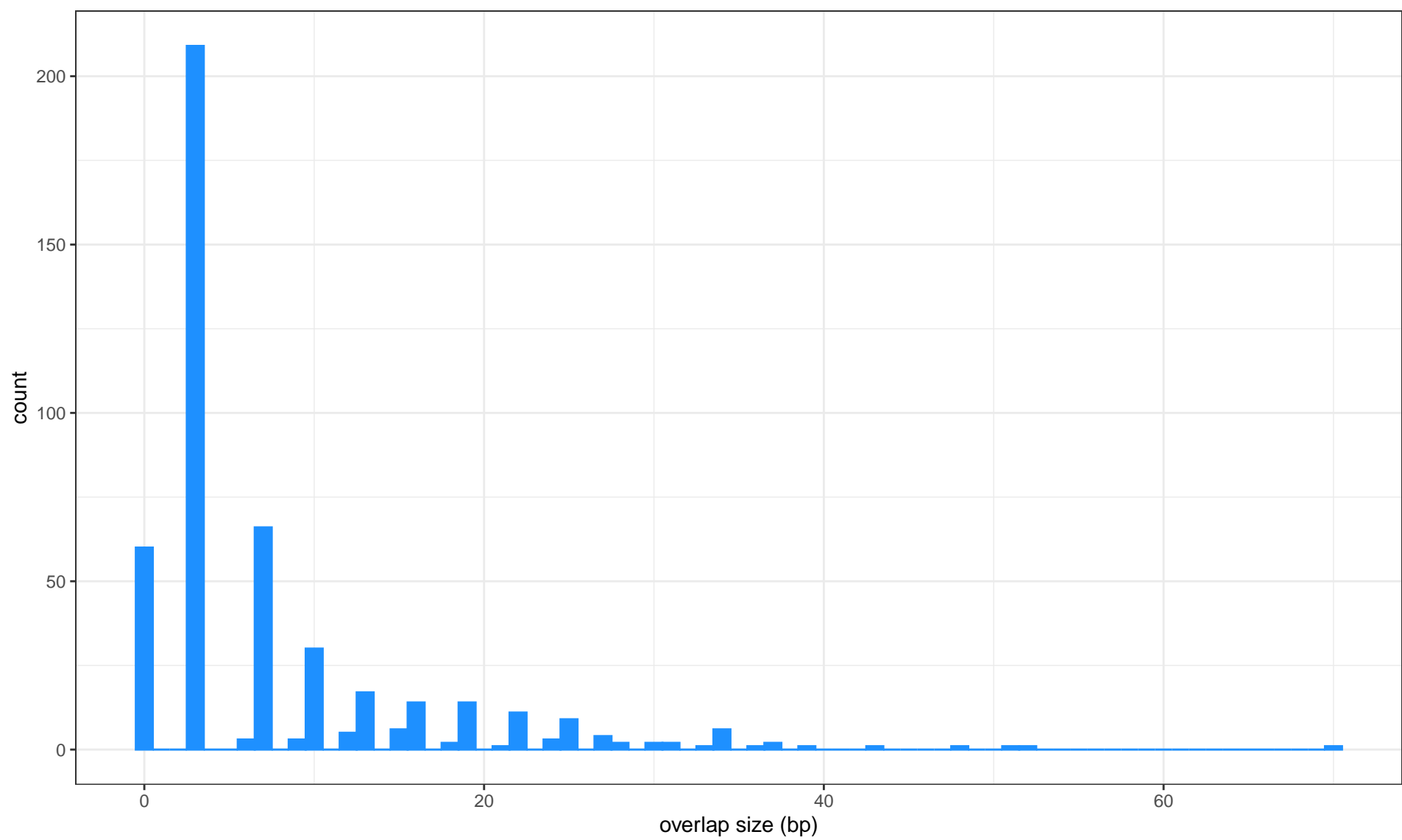

Supplement: S10 Fig — Plot includes all 5′ overlapping genes in the reference genome (479 of 5229 genes, around 9%). The x axis represents base pairs (bp) for which each gene is overlapping another one, and the y axis the number of genes that overlap by that bp length. Median overlap size is 3 bp. The data underlying this Figure can be found in https://doi.org/10.5281/zenodo.17378284. (PDF) [file pbio.3003569.s010.pdf]

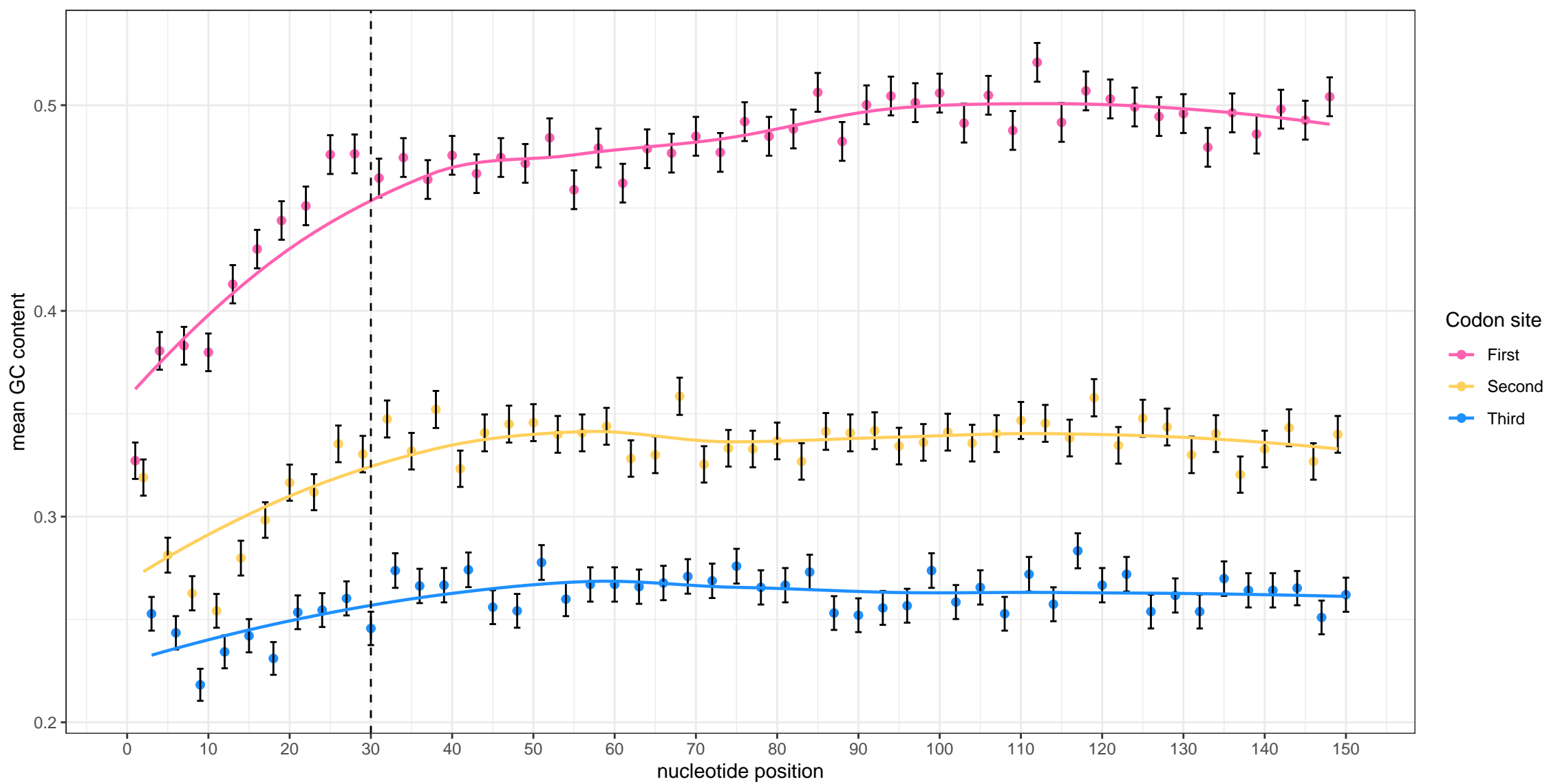

Supplement: S11 Fig — GC content is averaged at each nucleotide position across orthologs that are at least 50 codons long (n = 2,809). The x axis represents nucleotide positions relative to the start codon (i.e., the third nucleotide of the start codon is labeled as position 0). Error bars indicate the standard error of the mean (SEM). Dashed vertical black line marks the first 10 codons. Locally estimated scatterplot smoothing (LOESS) regression lines are included. The data underlying this Figure can be found in https://doi.org/10.5281/zenodo.17378284. (PDF) [file pbio.3003569.s011.pdf]

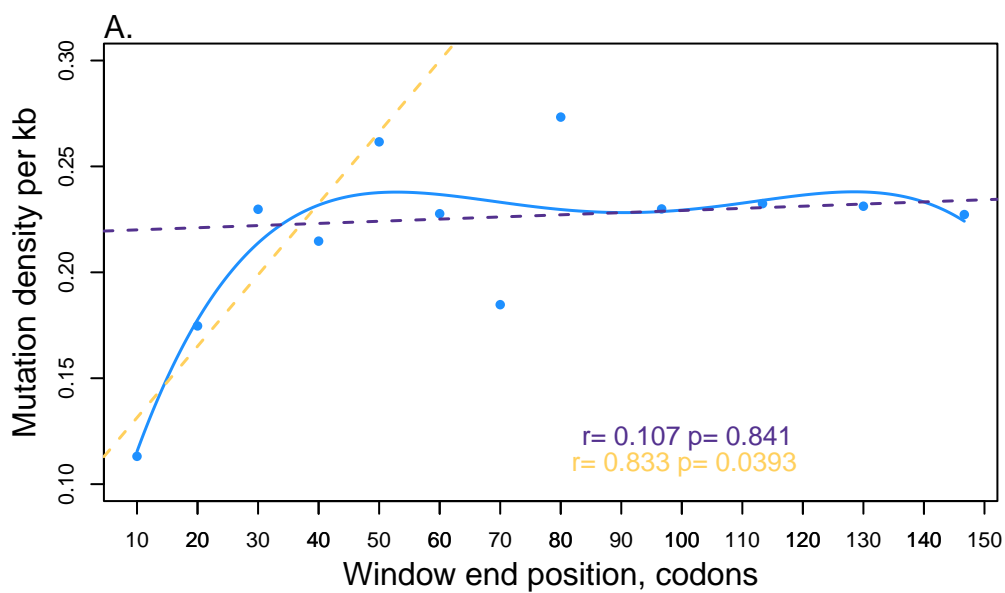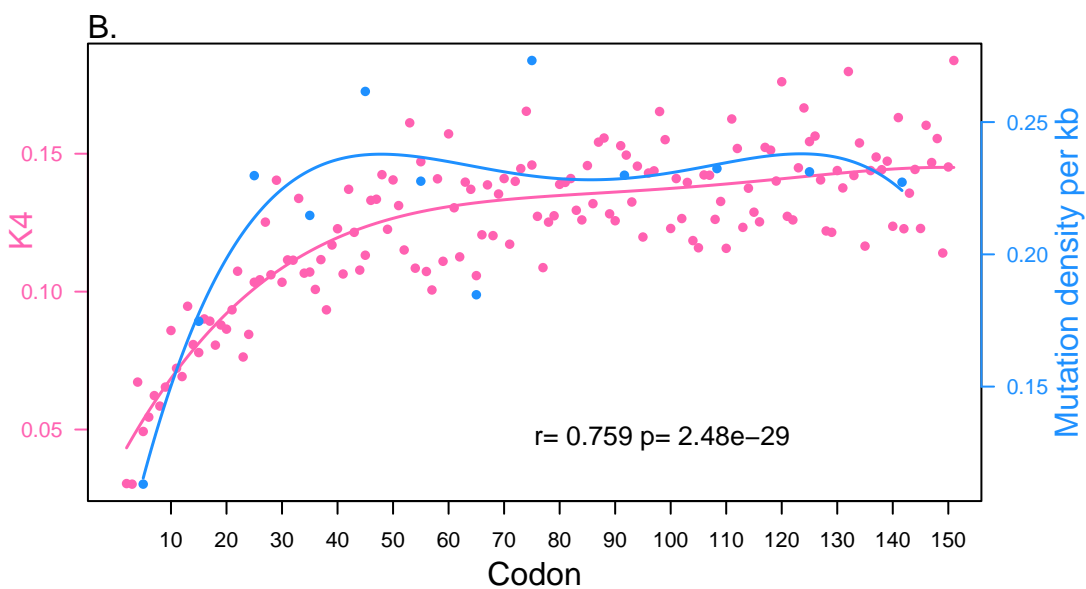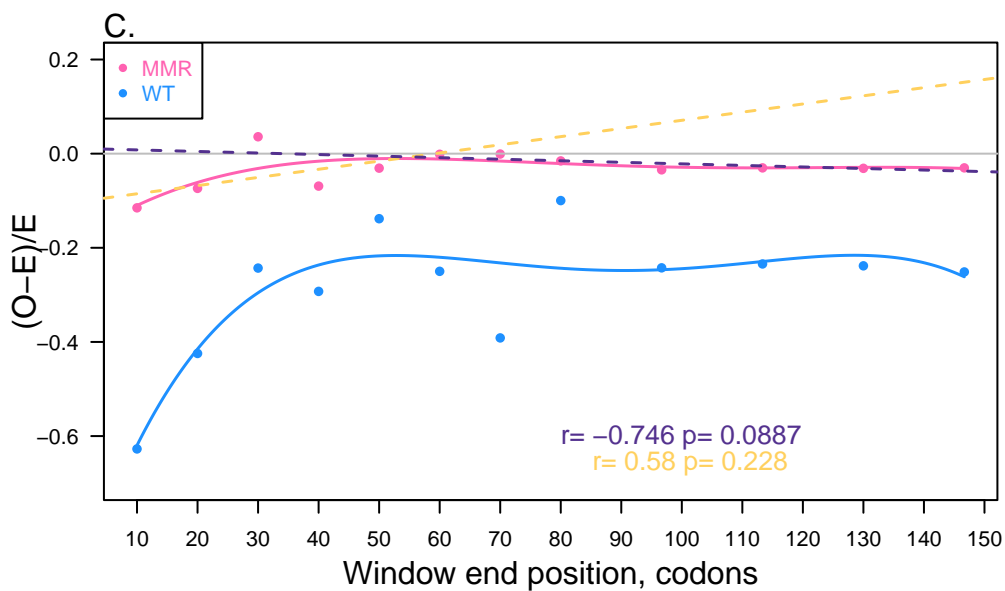

Supplement: S16 Fig — Same as Fig 5, but for E. coli non-overlapping genes only. A. Mutation density (mutations per kilobase, kb) as a function of within-gene position. The amount of sequence with each genic window, across all CDS, was determined, the density then being the number of mutations per bp, here scaled to kb. The blue line is a polynomial regression of degree 4. Yellow dashed line and yellow statistic are for the first 60 codons, dark purple dashed line and dark purple statistic are for the rest of the gene. Pearson correlation provided. B. Comparison of K4 values by codon and mutation density from WT lines. Mutation density is in blue with positions specified by mid-position of the window. K4 data per codon is in pink. Lines reflect polynomial regression of degree 4. To determine pseudo-significance, we interpolate values for each codon by fitting to the blue polynomial line. These values are then correlated against the observed K4 values (Pearson correlation shown). C. Deviation from null (O − E)/E for WT (alternative metric for data in panel A) and from MA lines that have MMR deleted. The first 60 codons are positively correlated for the WT data (statistics as panel A), but the MMR deletion data is not (Pearson correlation r = 0.78, P-value = 0.06). Dark purple dashed line is regression for data post-60 codons for MMR-deficient data, yellow dashed line for data within 60 codons. The pink line is the polynomial regression for MMR-deficient the blue for WT. The horizontal gray line marks (O − E)/E = 0. In all panels mutational data from Wei and colleagues [56]. The data underlying this Figure can be found in https://doi.org/10.5281/zenodo.17378284. (PDF) [file pbio.3003569.s016.pdf]

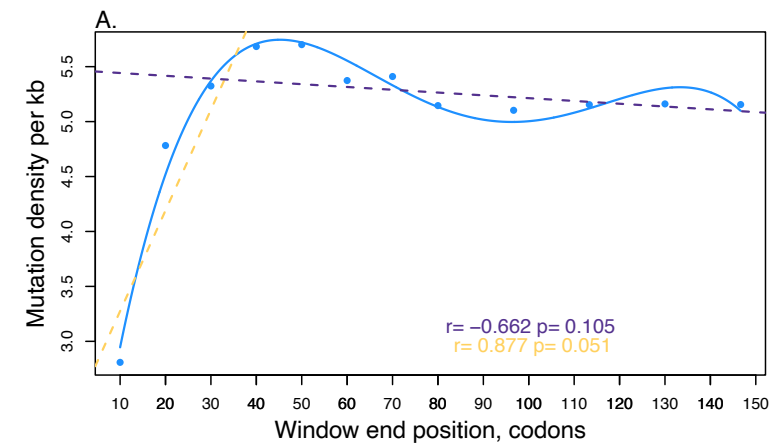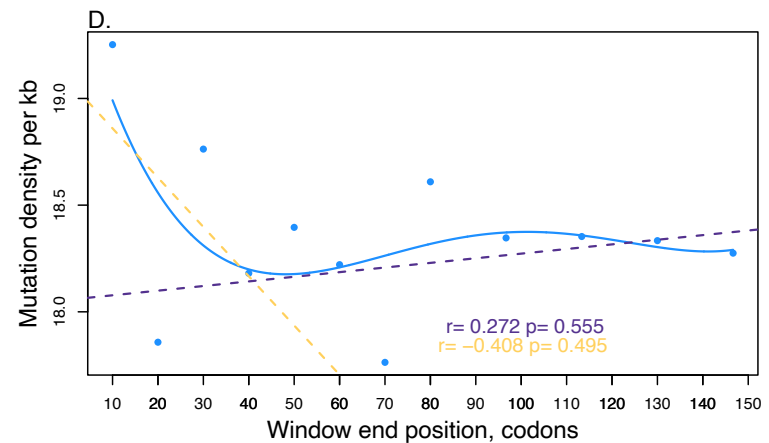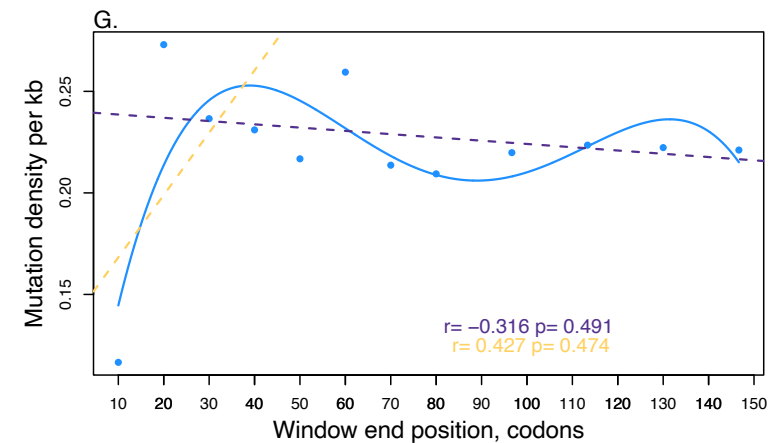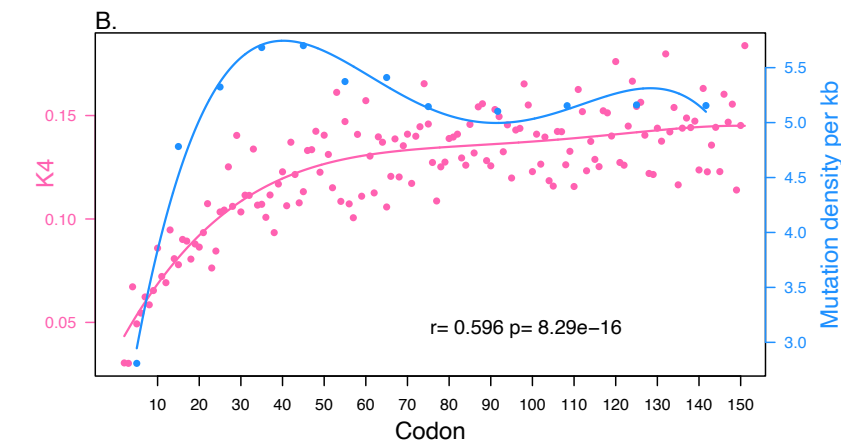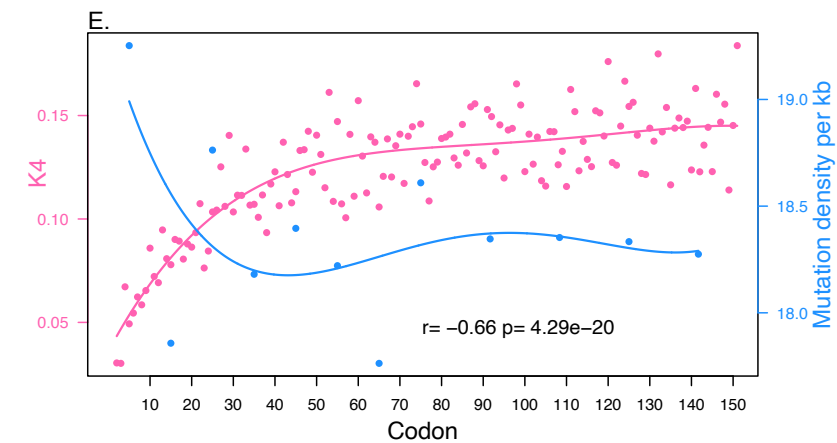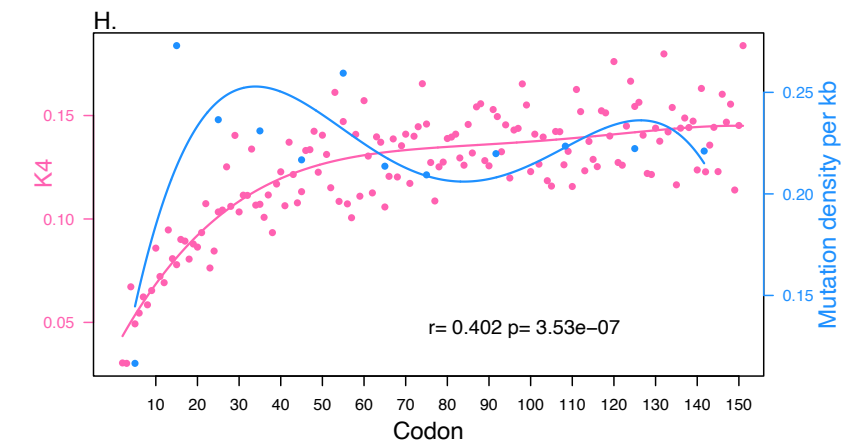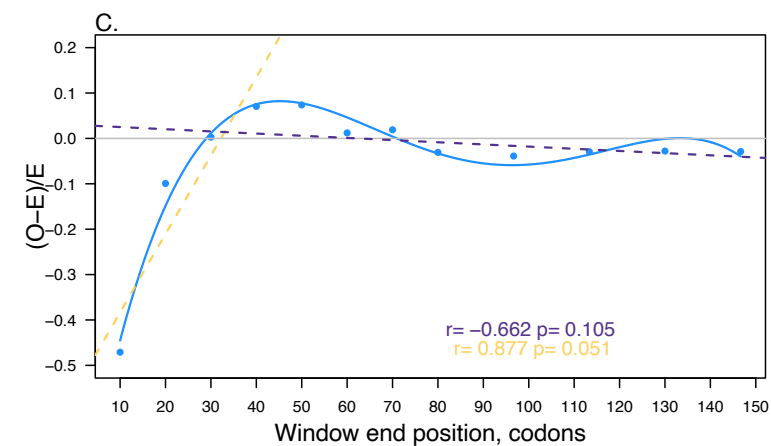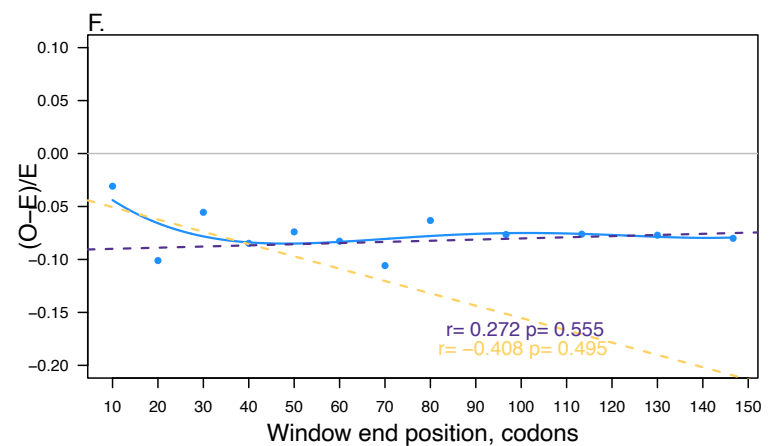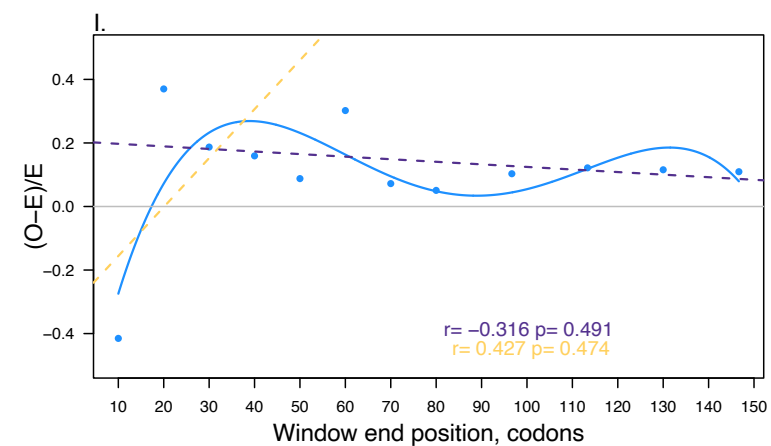

Supplement: S17 Fig — Same as Fig 5, but for WT E. coli mutational data from Zhang and colleagues [57] for high-depth samples (A–C), and for low-depth samples (D–F), and for E. coli mutational data from Foster and colleagues [58] (G–I). In all, panels yellow dashed lines and yellow statistic consider the first 50 codons, while dark purple considers the rest of the gene (unlike the codon 60 threshold set in Fig 5). The data underlying this Figure can be found in https://doi.org/10.5281/zenodo.17378284. (PDF) [file pbio.3003569.s017.pdf]

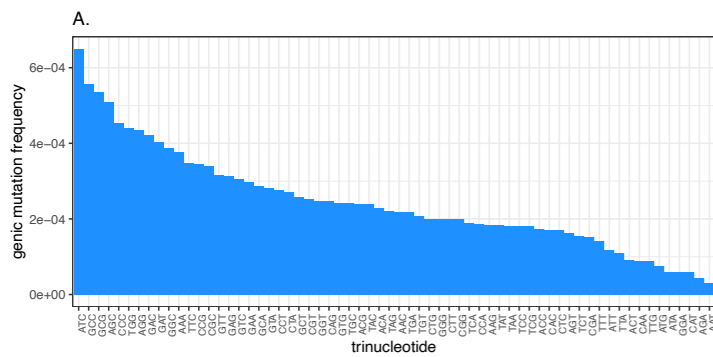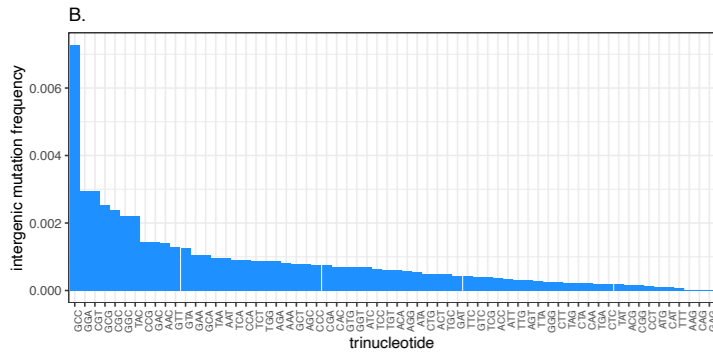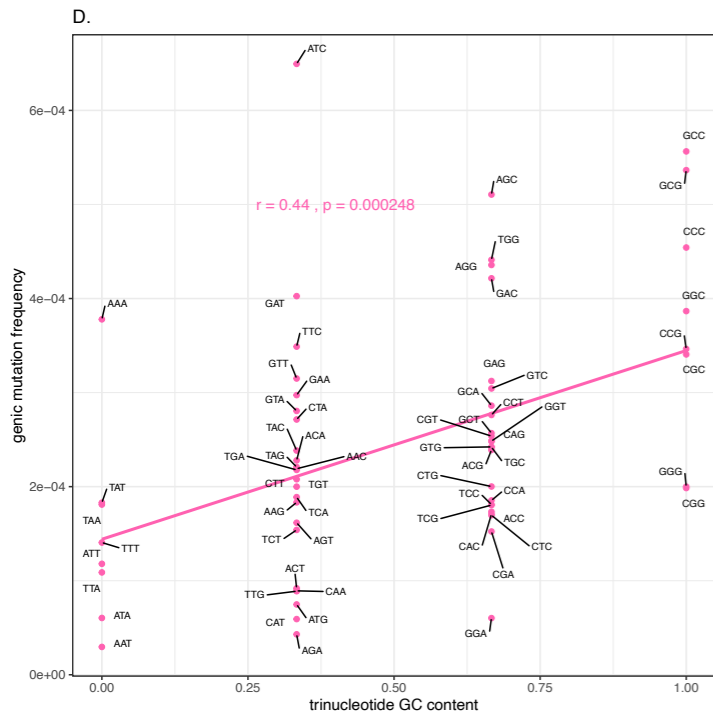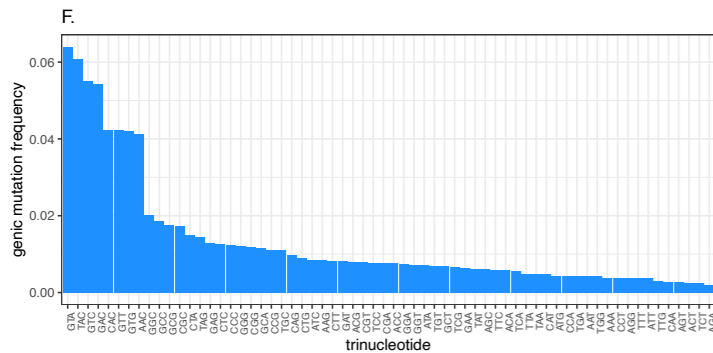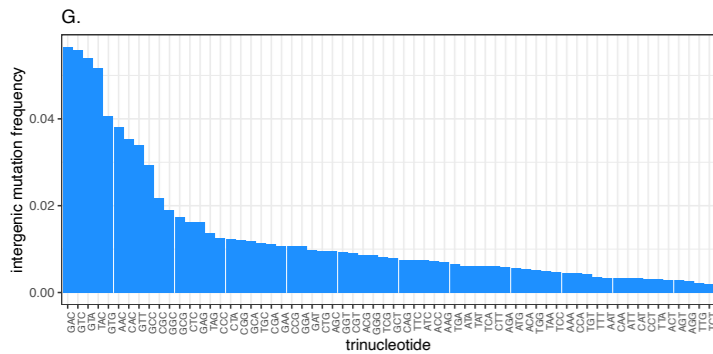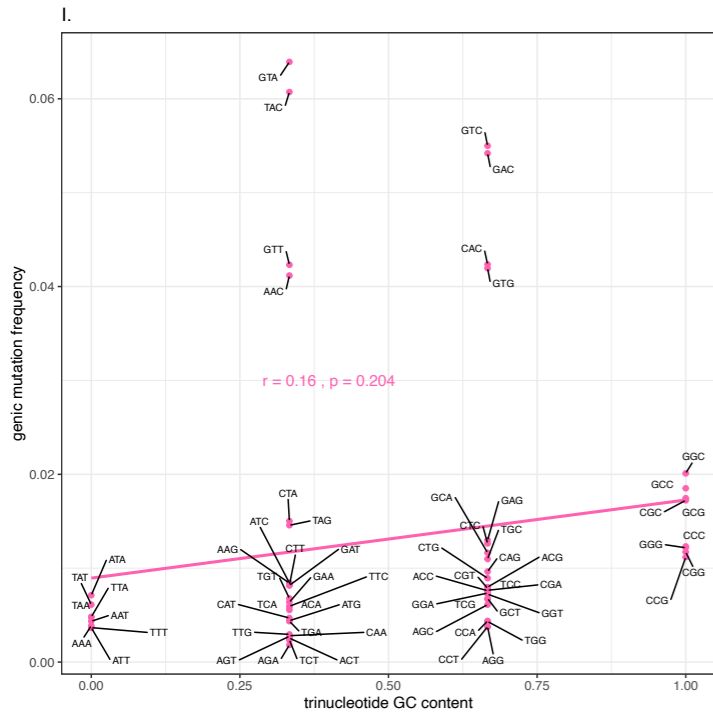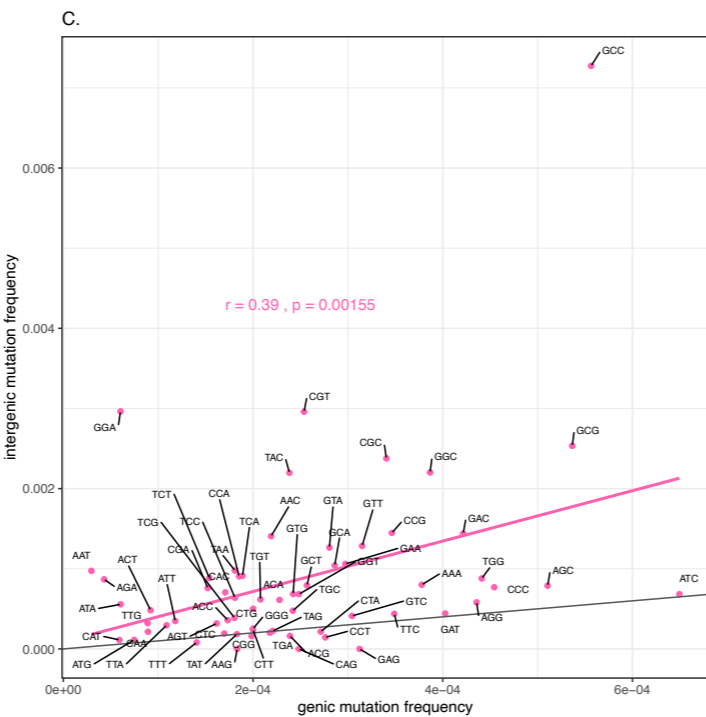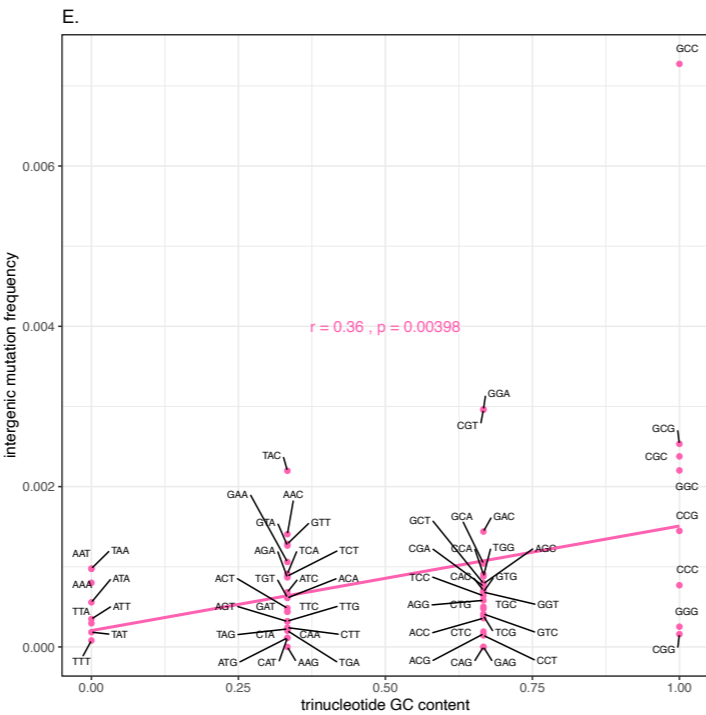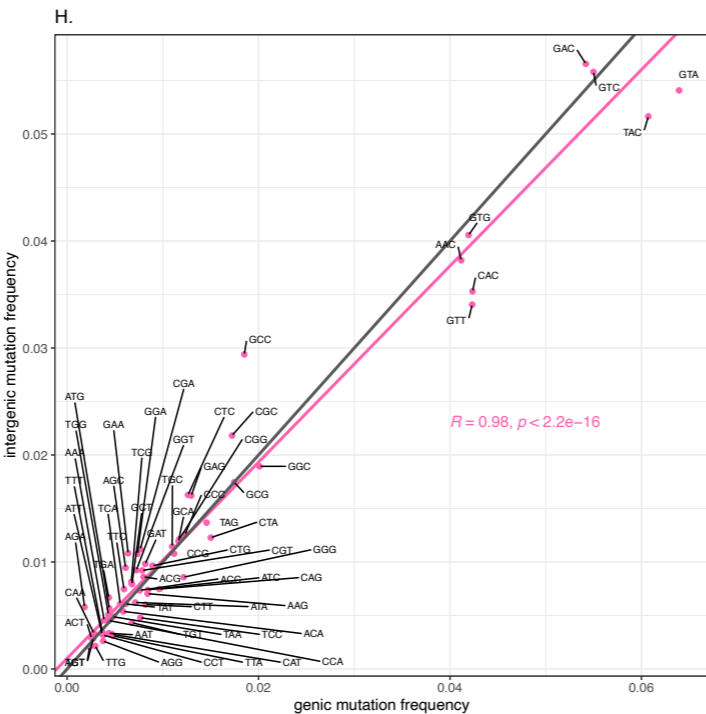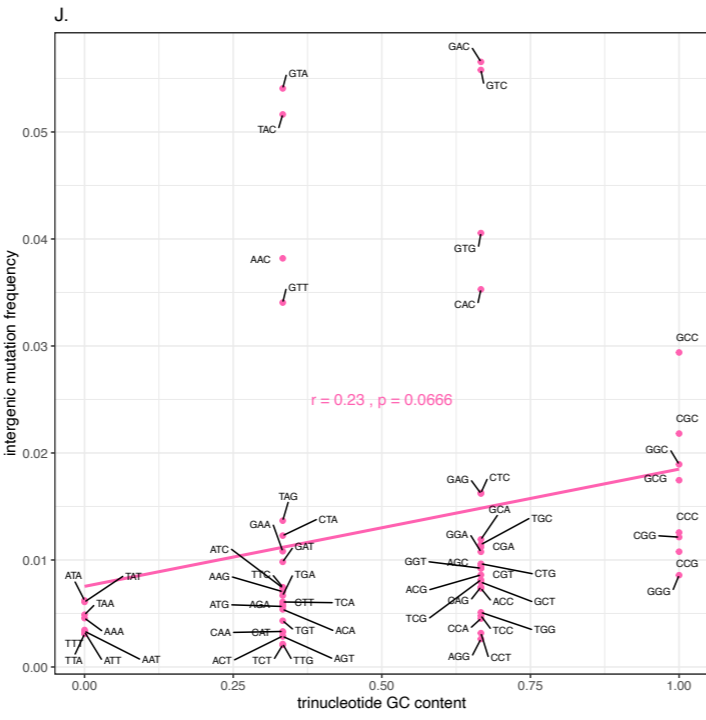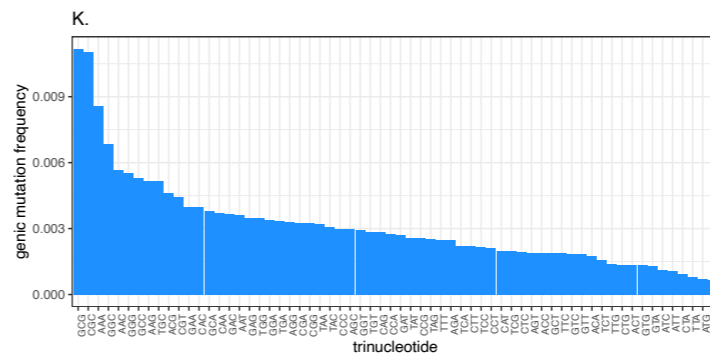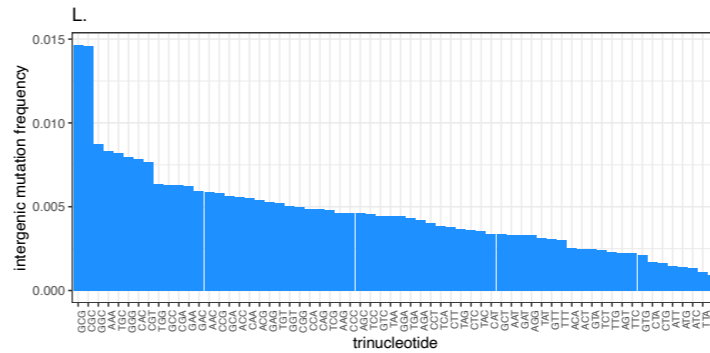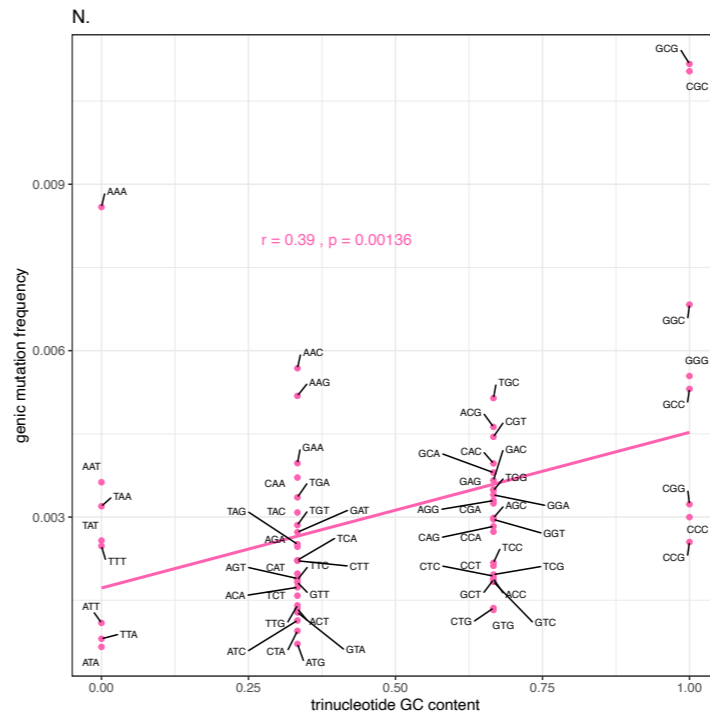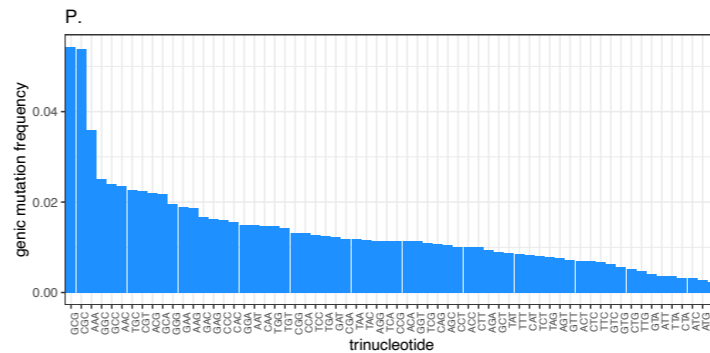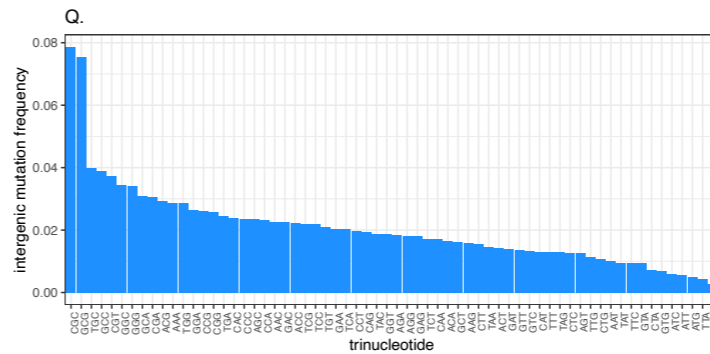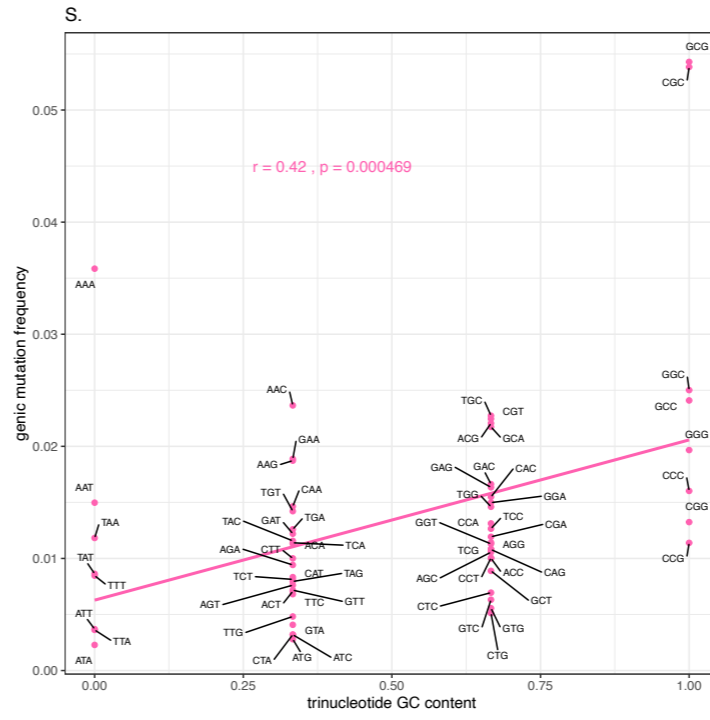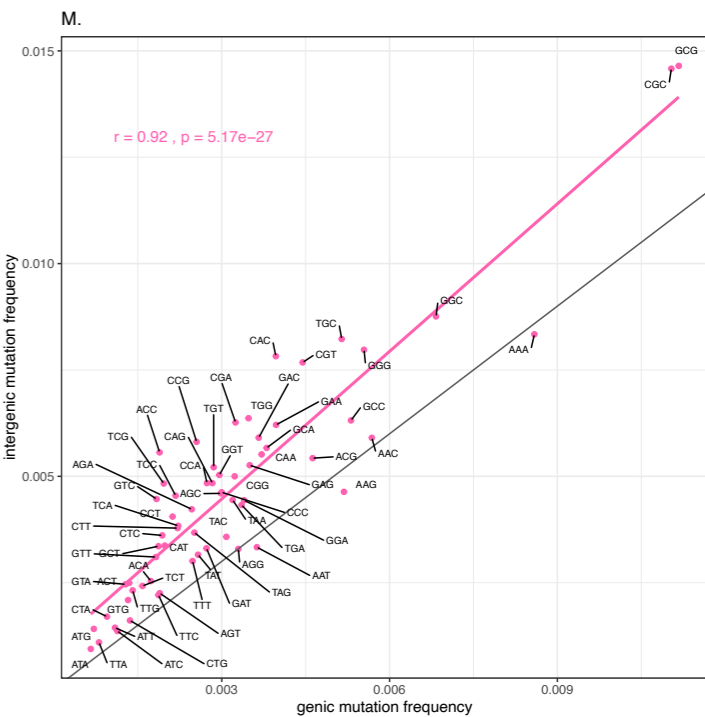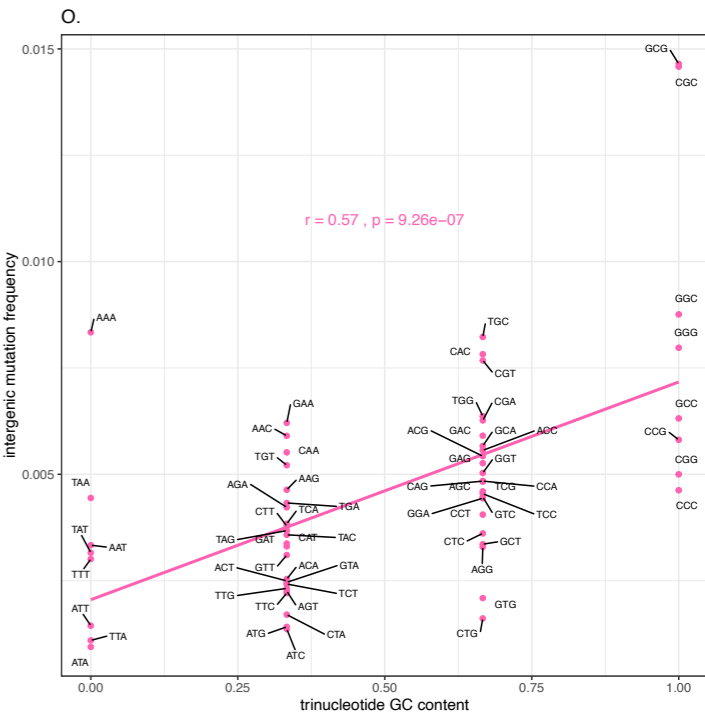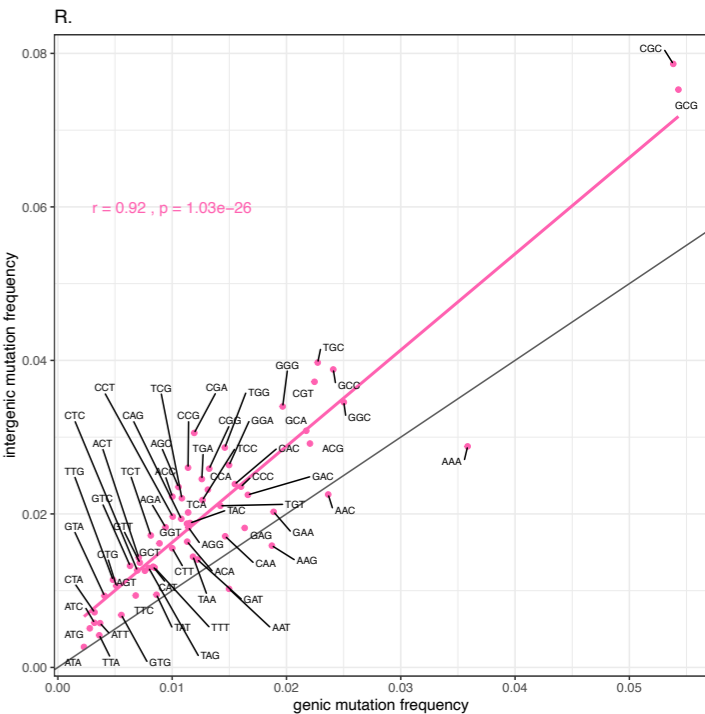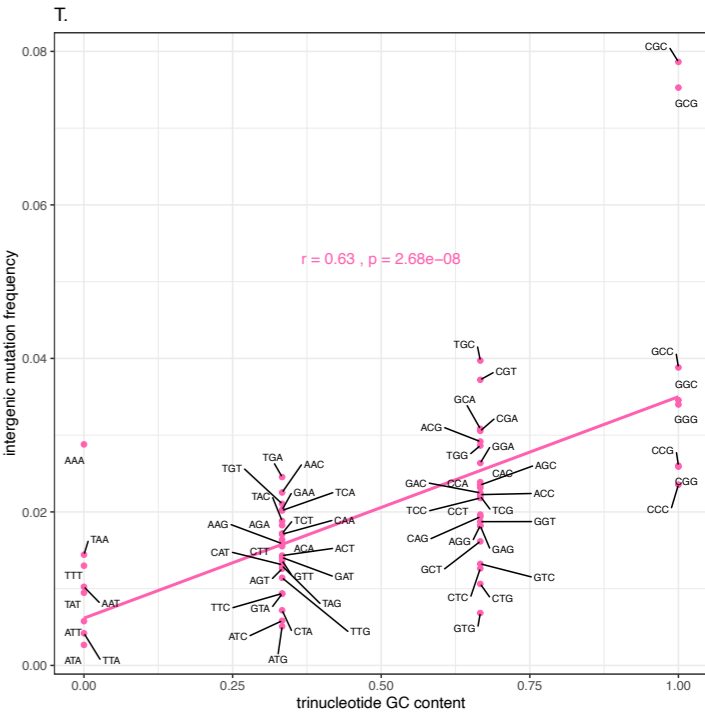

Supplement: S18 Fig — Observed trinucleotide mutation frequencies in A. genic regions and B. intergenic regions. Mutation frequency refers to mutation count per occurrence of ancestor base. Trinucleotide mutations are such that the middle base is the mutated base. Trinucleotides on the x axis are rank-ordered from most to least frequent. Mutation data for E. coli from Wei and colleagues [56] WT samples. C. The same trinucleotide frequencies in A and B, plotted against each other. Pink is a linear regression line, Pearson correlation, and respective P-value displayed. Dark gray line is a regression line with slope 1 and intercept 0 (i.e., perfect correlation between the two regions). Note if the pink line sits above the perfect correlation line, it represents higher trinucleotide mutation frequencies in intergenic regions. D–E. The same trinucleotide frequencies in A–B, plotted against trinucleotide GC content for genic and intergenic trends, respectively. Pearson correlation and respective P-value displayed. F–J same as A–E, but for MMR-deficient Wei and colleagues [56] samples. K–O same as A–E, but for mutation data for Escherichia coli from Zhang and colleagues [57] for samples sequences at higher depth. P–T same as K–O, but for samples sequenced at lower depth. The data underlying this Figure can be found in https://doi.org/10.5281/zenodo.17378284. (PDF) [file pbio.3003569.s018.pdf]

A.

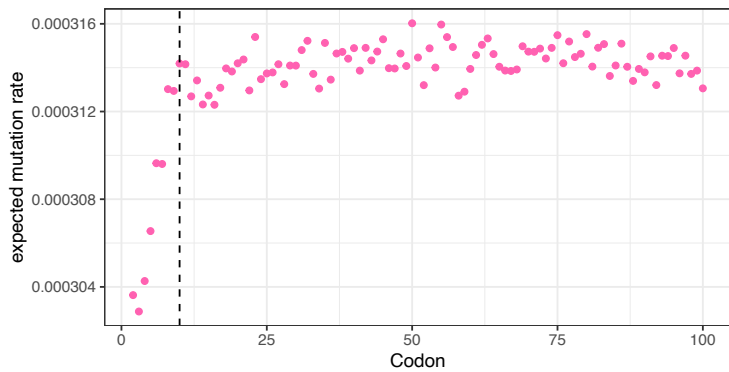

D.

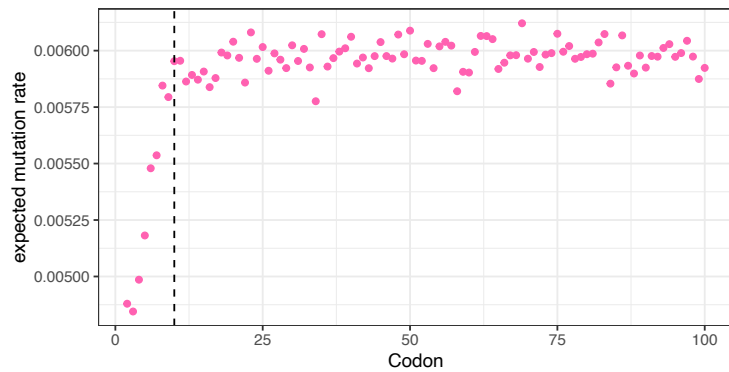

B.

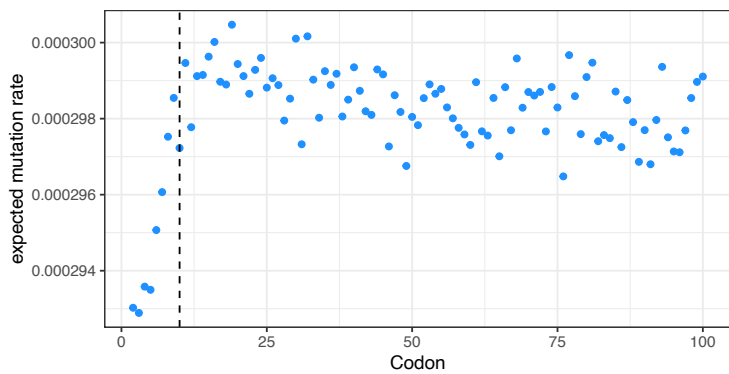

E.

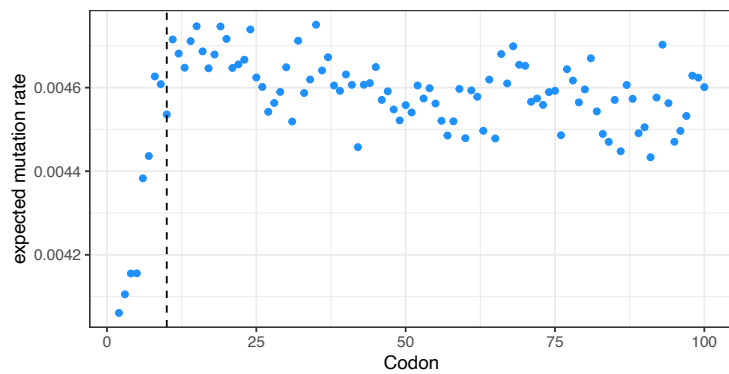

C.

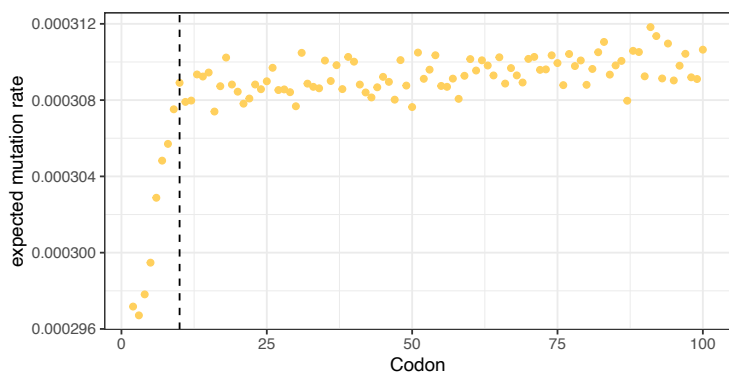

F.

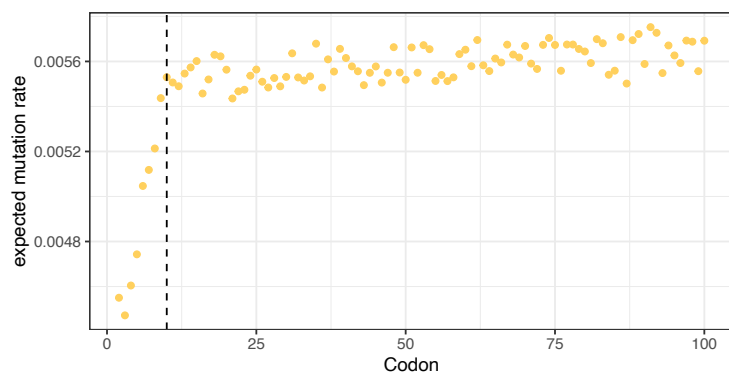

Supplement: S19 Fig — Mutation refers to mononucleotide changes. Position on the x axis refers to codons. Dashed vertical black line marks the first 10 codons. For A–C, mutation data for E. coli from Wei and colleagues [56], and for D–F mutational data for E. coli from Zhang and colleagues [57] samples sequenced at higher depth. The data underlying this Figure can be found in https://doi.org/10.5281/zenodo.17378284. (PDF) [file pbio.3003569.s019.pdf]

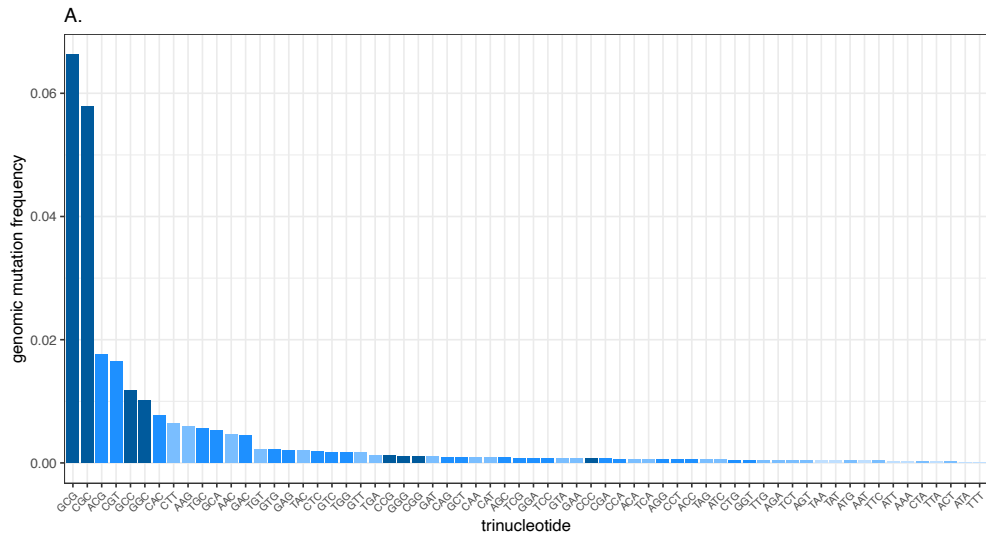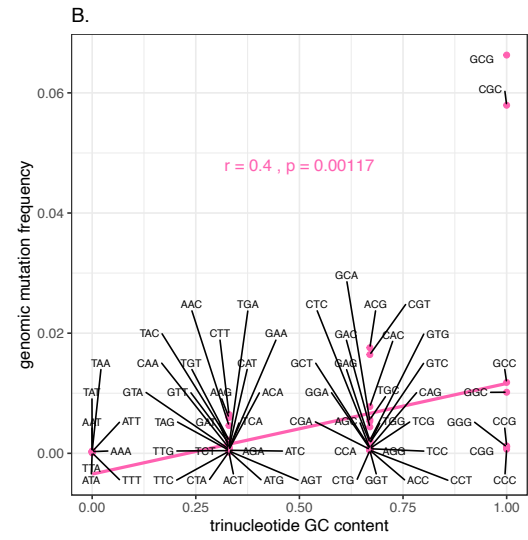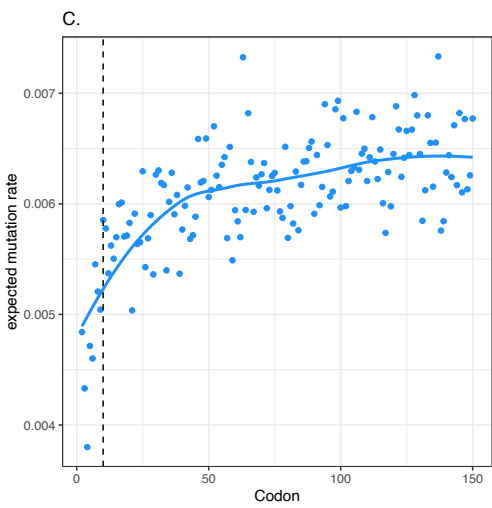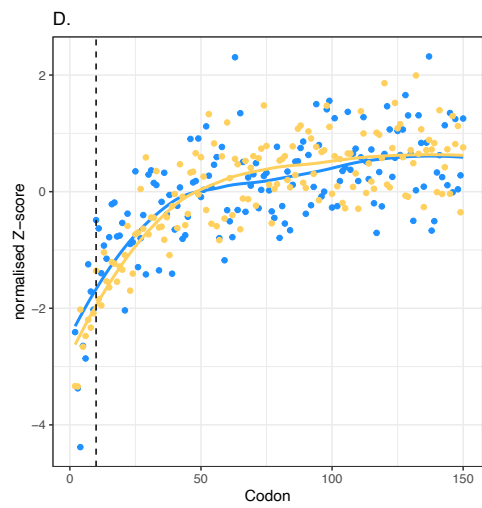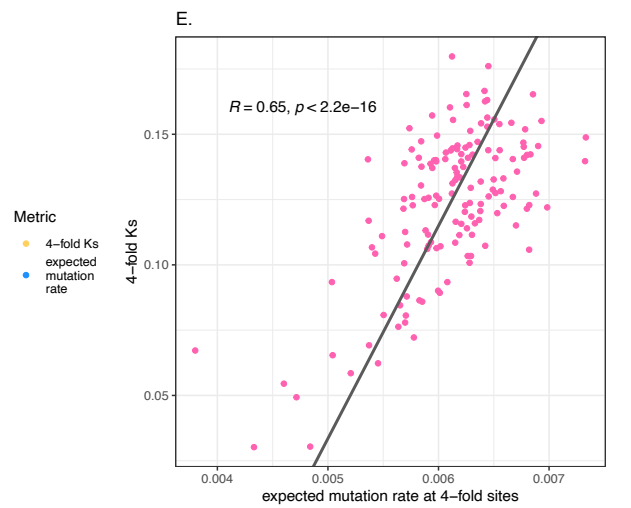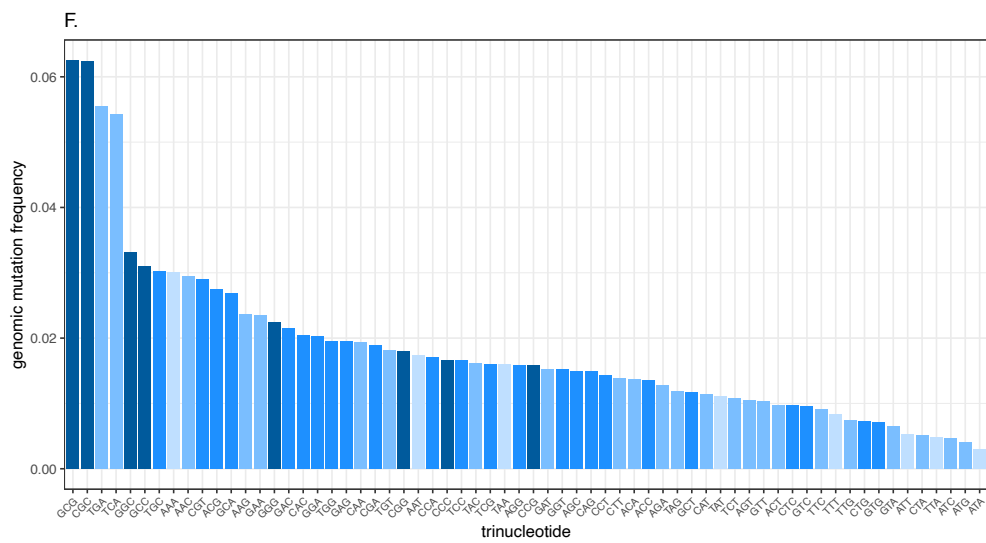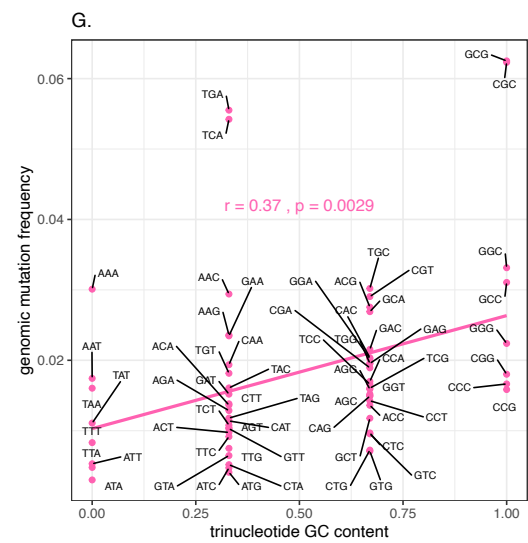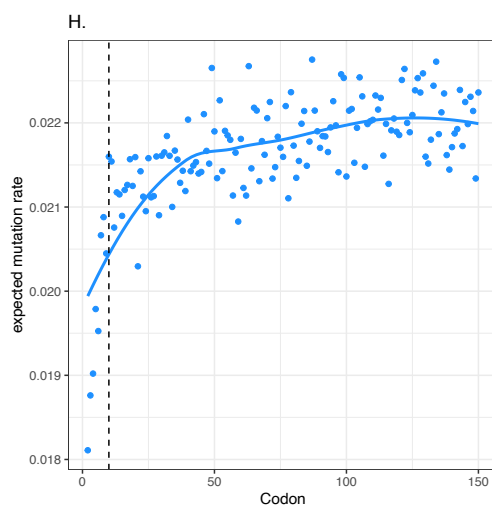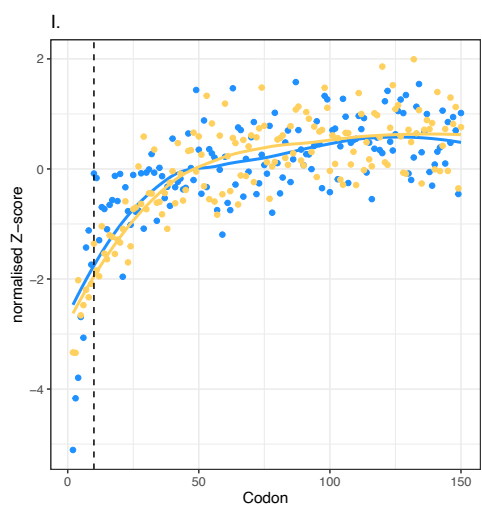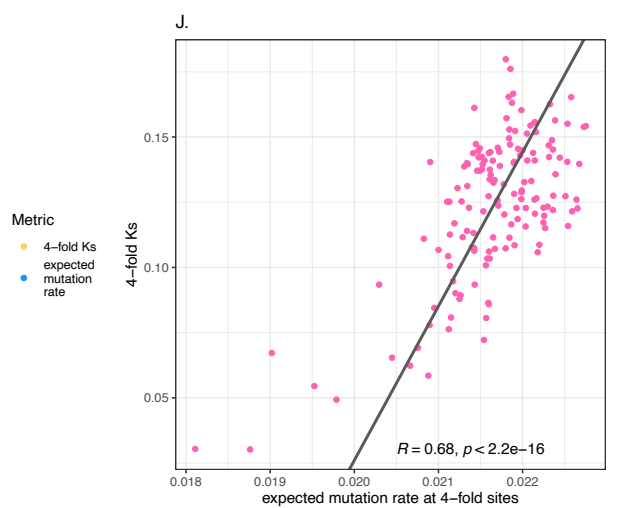

Supplement: S20 Fig — A. Observed genomic trinucleotide mutational frequencies rank-ordered from most to least frequent in Escherichia coli. Mutational data for E. coli from Zhang and colleagues [57] samples sequenced at higher depth. Mutation frequency refers to mutation count per occurrence of ancestor base. Trinucleotide mutations are such that the middle base is the mutated base. Trinucleotides on the x axis are rank-ordered by frequency and bars are color-coded by trinucleotide GC content. B. The same genomic mutation frequencies as in A, plotted against trinucleotide GC content. Line represents linear regression and Pearson correlation with respective P-value is also shown. C. Expected trinucleotide mutation rates by codon position, predicted by trinucleotide genomic mutational rates and genomic trinucleotide content. Trinucleotide mutations are such that the middle base is the mutated base, and it occurs at third sites in 4-fold degenerate codons. D. Comparison of expected mutational rates in C with E. coli conservation trends by codon position at 4-fold degenerate sites (4-fold Ks, as seen in S5 Fig). Both metrics are normalized by Z score. E. Comparison of expected trinucleotide mutation rates and K4 by position without Z transformation. Pearson correlation data is shown. Line is the orthogonal (major axes) regression line. F–J as A–E, but for E. coli from Zhang and colleagues [57] samples sequenced at lower depth. For panels C, D, H, and I, position on the x axis refers to absolute number of codons (where the start codon is position 1), and the dashed vertical black line marks the first 10 codons. Locally estimated scatterplot smoothing (LOESS) regression lines are also provided. The data underlying this Figure can be found in https://doi.org/10.5281/zenodo.17378284. (PDF) [file pbio.3003569.s020.pdf]

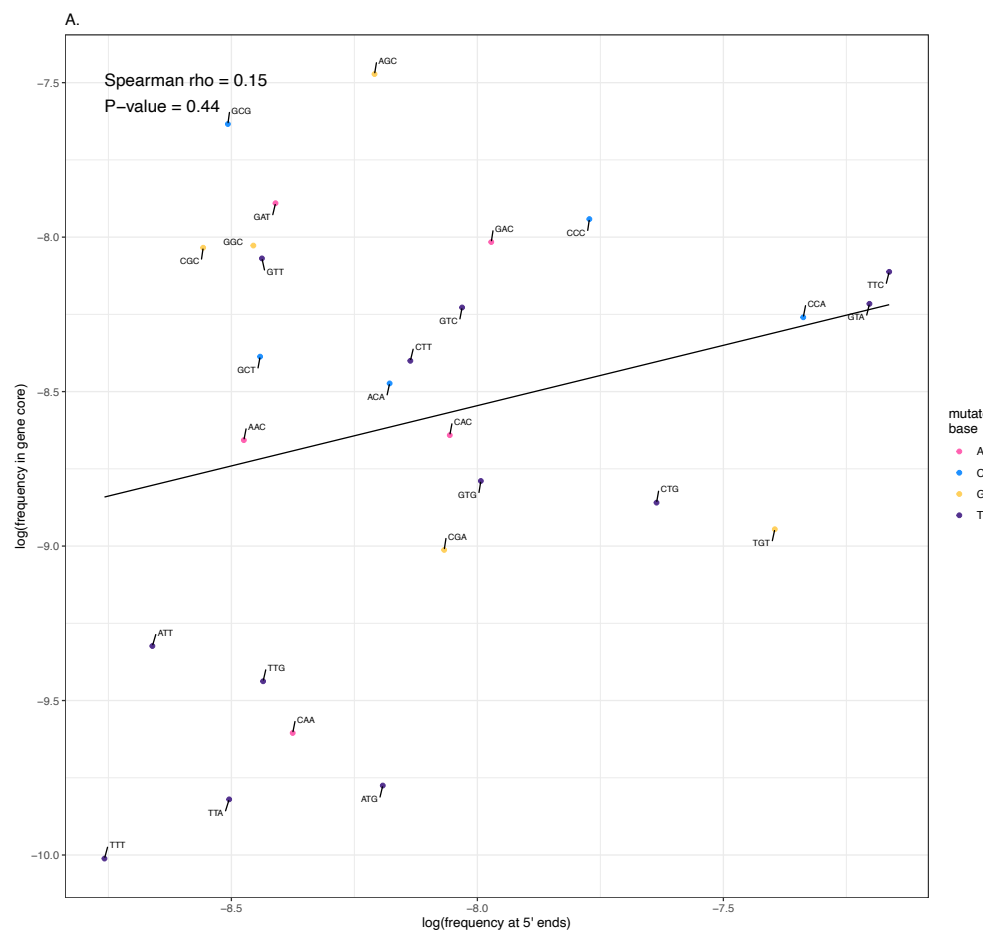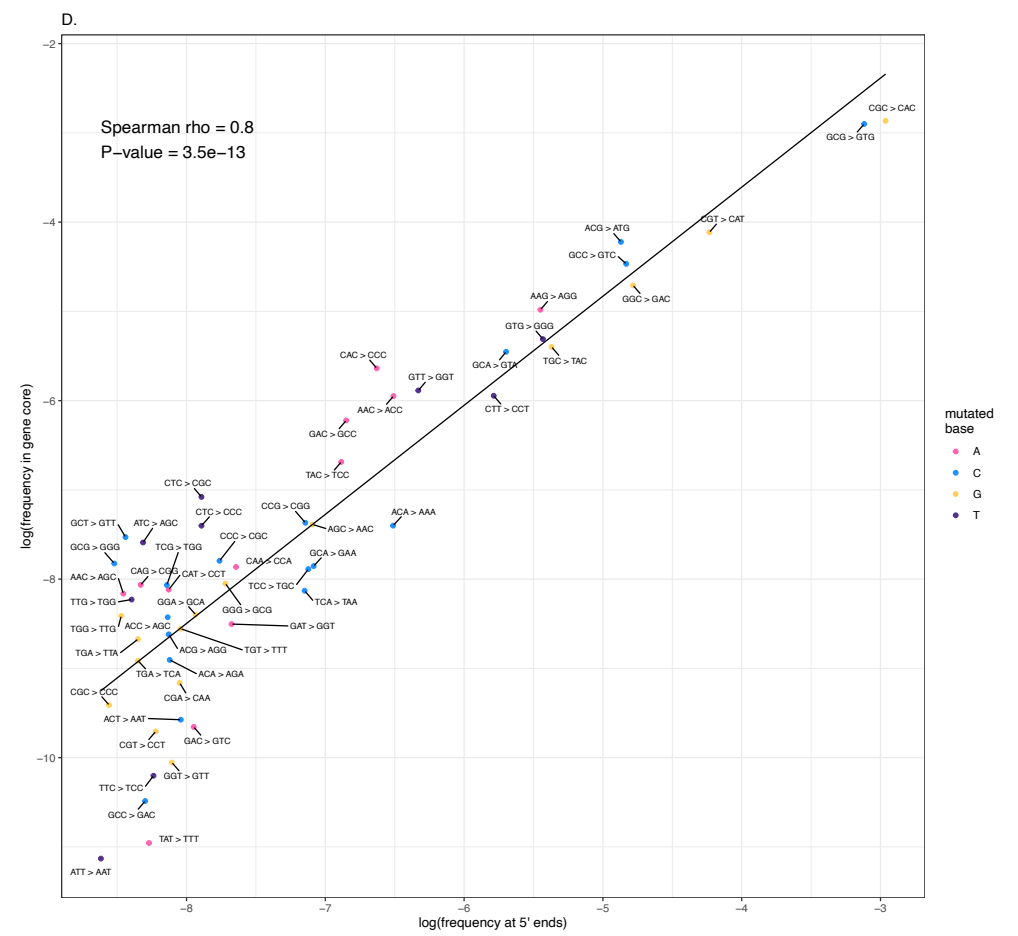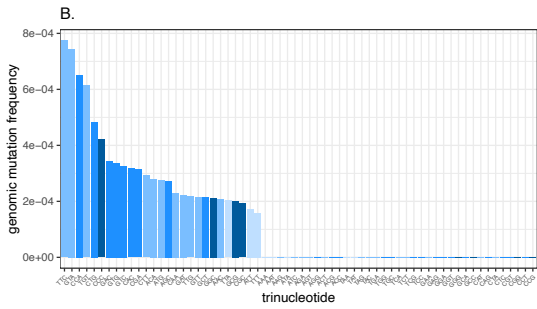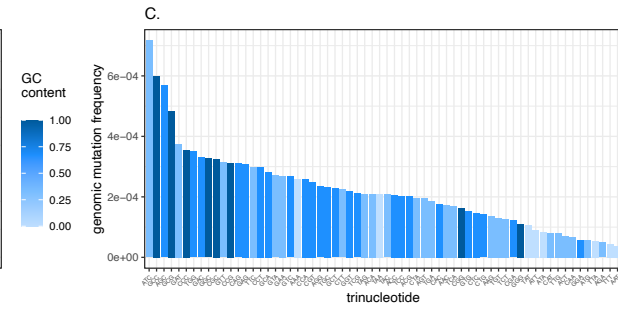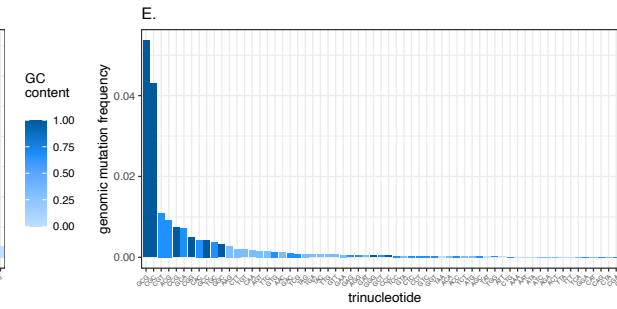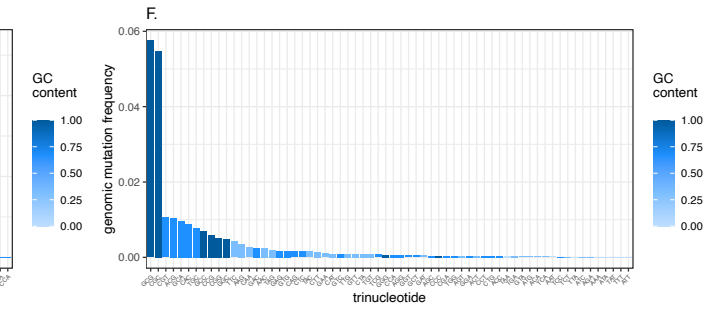

Supplement: S21 Fig — A. Comparison between each possible trinucleotide mutation in the two gene regions. Points are color-coded by ancestral base. B. Trinucleotide mutational frequencies at 5′ ends rank ordered from largest to smallest. C. Trinucleotide mutational frequencies in gene cores rank-ordered from largest to smallest. Bars in B and C are color-coded by GC content within the trinucleotide. For all plots, trinucleotide mutations are such that the middle base is the mutated base. For A–C mutational data from Wei and colleagues [56], and D–F are the same but with mutation data from Zhang and colleagues [57]. For all panels 5′ ends include the first 20 codons, while gene cores refer to the rest of the CDS. The data underlying this Figure can be found in https://doi.org/10.5281/zenodo.17378284. (PDF) [file pbio.3003569.s021.pdf]

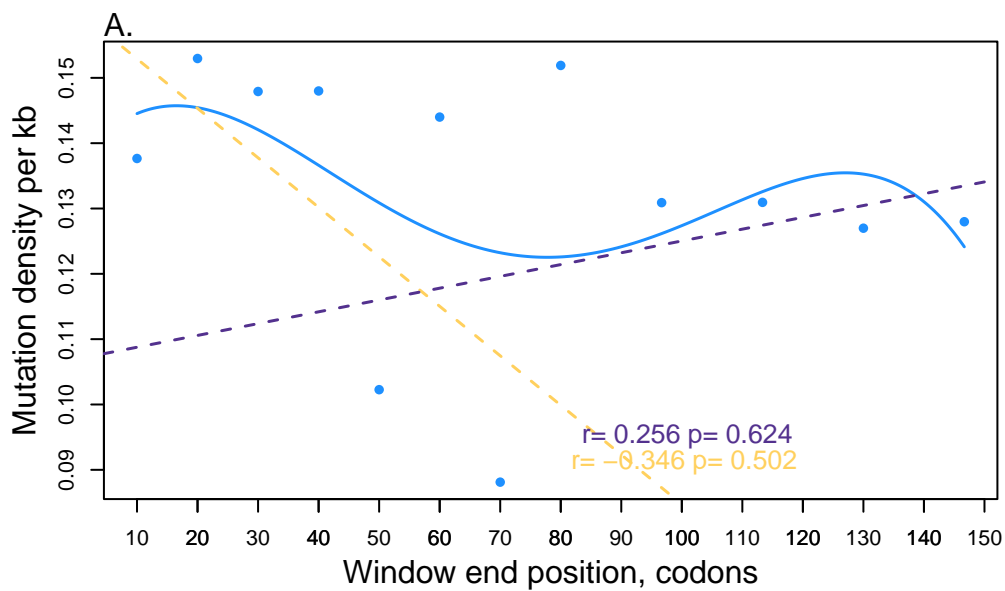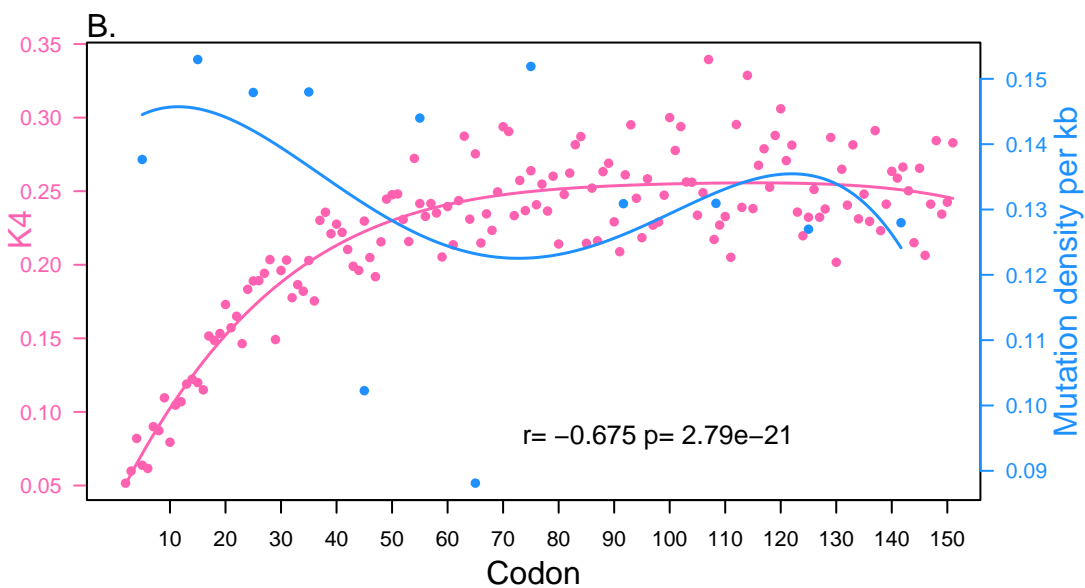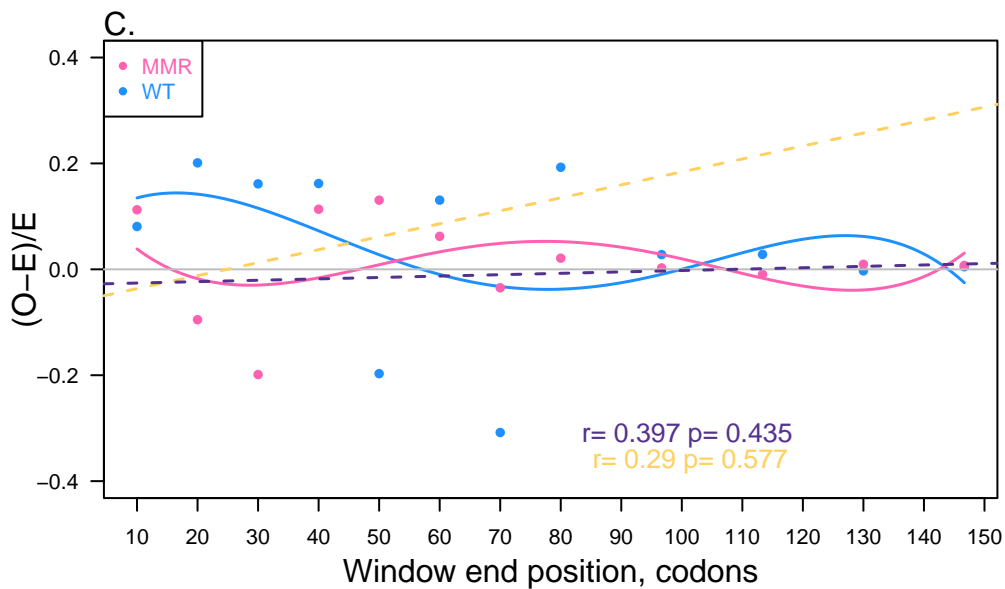

Supplement: S22 Fig — A. Mutation density (mutations per kilobase, kb) as a function of within gene position. The amount of sequence with each genic window, across all CDS, was determined, the density then being the number of mutations per bp, here scaled to kb. The blue line is a polynomial regression of degree 4. Yellow dashed line and yellow statistic are for the first 60 codons, dark purple dashed line and dark purple statistic are for the rest of the gene. Pearson correlation provided. B. Comparison of K4 values by codon and mutation density from WT lines. Mutation density is in blue with positions specified by mid-position of the window. K4 data per codon is in pink. Lines reflect polynomial regression of degree 4. To determine pseudo-significance, we interpolate values for each codon by fitting to the blue polynomial line. These values are then correlated against the observed K4 values (Pearson correlation shown). C. Deviation from null (O − E)/E for WT (alternative metric for data in panel A) and from MA lines that have MMR deleted. The first 60 codons are positively correlated for the WT data (statistics as panel A), but the MMR deletion data is not (Pearson correlation r = 0.78, P-value = 0.06). Dark purple dashed line is regression for data post-60 codons for MMR-deficient data, yellow dashed line for data within 60 codons. The pink line is the polynomial regression for MMR-deficient, the blue for WT. The horizontal gray line marks (O − E)/E = 0. In all panels mutational data for WT is combined from Castañeda-Garcia and colleagues [60] and Kucukyildirim and colleagues [61], while data for MMR-deficient samples is from Castañeda-Garcia and colleagues [60] only. The data underlying this Figure can be found in https://doi.org/10.5281/zenodo.17378284. (PDF) [file pbio.3003569.s022.pdf]

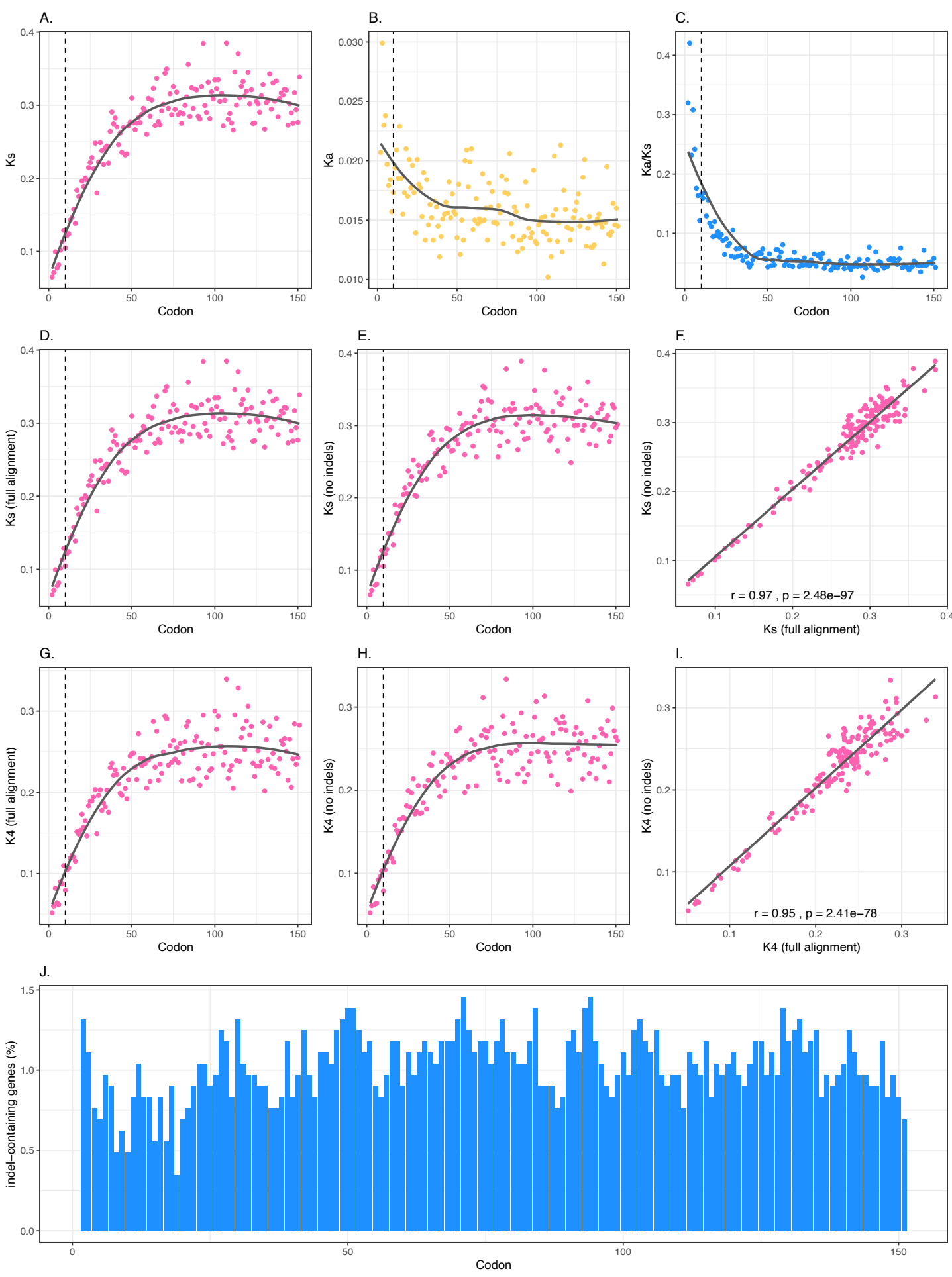

Supplement: S23 Fig — A. Synonymous substitution rates (Ks); B. non-synonymous substitution rates (Ka), and C. the ratio between the two (Ka/Ks). A–C plots include orthologs that are at least 180 codons in length (n = 1,698). For A–C, the x axis represents absolute codon position (i.e., the start codon is codon 1). Dashed vertical black line marks the first 10 codons. Locally estimated scatterplot smoothing (LOESS) regression lines are included. Note that codon position here is by reference to the codon position in the alignment. D–I Comparison of K4 and Ks values for alignments with and without indels in the focal lineage alignment. We calculate K by codon in one instance assigning codon position by position within the alignment and in the second instance by first removing aligned codons where the focal lineage has an indel. D is Ks for the full alignment, E Ks for the indel removed case, and F a scatter plot comparing the two with orthogonal regression lines and Pearson correlation. The following set (G, H, I) are the same but for K4. Note removal of alignment indels in the focal lineage prior to codon position categorization makes no meaningful difference. J. Proportion of genes/alignments with an indel at each codon position. The data underlying this Figure can be found in https://doi.org/10.5281/zenodo.17378284. (PDF) [file pbio.3003569.s023.pdf]
