# Supplementary material for: Bacterial gene 5′ ends have unusual mutation rates that can mislead tests of selection
Source: PLoS Biol. 2025 Dec 15;23(12):e3003569. doi: 10.1371/journal.pbio.3003569 (PMC12725619; doi:10.1371/journal.pbio.3003569)
Supplement: S12 Fig — For E. coli, A. is non-operonic genes’ GC3 by position, B. the operonic GC3, and C. the comparison of the two employing orthogonal regression and Pearson correlation. D–F is the same, but for codon usage bias. G–L are the same as A–F, but for B. subtilis. The data underlying this Figure can be found in https://doi.org/10.5281/zenodo.17378284. (PDF) [file pbio.3003569.s012.pdf]

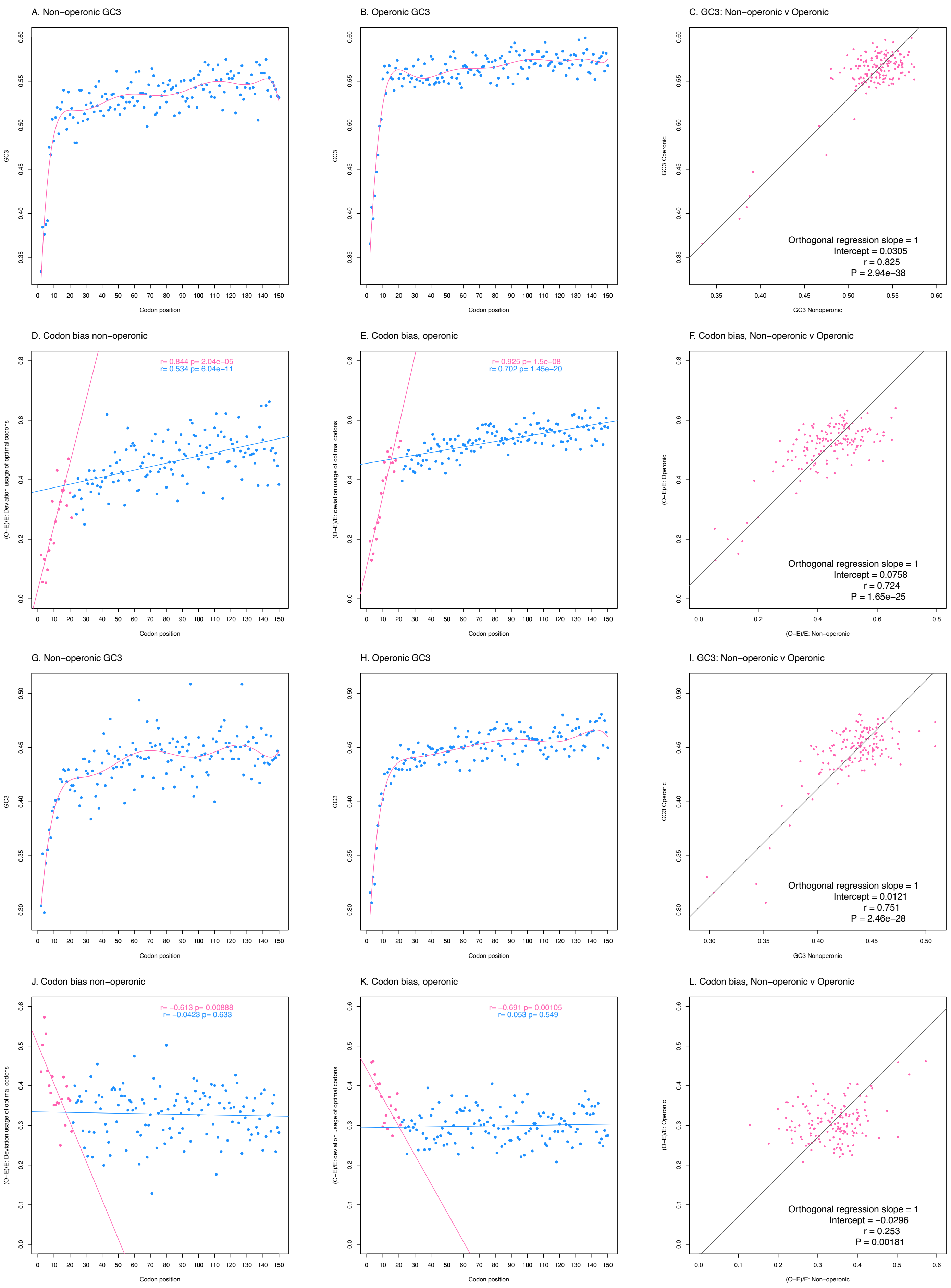
A. Non−operonic GC3
GC3
Codon position
B. Operonic GC3
GC3
Codon position
C. GC3: Non−operonic v Operonic
GC3 Operonic
GC3 Nonoperonic
Orthogonal regression slope = 1
Intercept = 0.0305
r = 0.825
P = 2.94e−38
D. Codon bias non−operonic
r= 0.844 p= 2.04e−05
r= 0.534 p= 6.04e−11
(O−E)/E: Deviation usage of optimal codons
Codon position
E. Codon bias, operonic
r= 0.925 p= 1.5e−08
r= 0.702 p= 1.45e−20
(O−E)/E: deviation usage of optimal codons
Codon position
F. Codon bias, Non−operonic v Operonic
(O−E)/E: Operonic
(O−E)/E: Non−operonic
Orthogonal regression slope = 1
Intercept = 0.0758
r = 0.724
P = 1.65e−25
G. Non−operonic GC3
GC3
Codon position
H. Operonic GC3
GC3
Codon position
I. GC3: Non−operonic v Operonic
GC3 Operonic
GC3 Nonoperonic
Orthogonal regression slope = 1
Intercept = 0.0121
r = 0.751
P = 2.46e−28
J. Codon bias non−operonic
r= −0.613 p= 0.00888
r= −0.0423 p= 0.633
(O−E)/E: Deviation usage of optimal codons
Codon position
K. Codon bias, operonic
r= −0.691 p= 0.00105
r= 0.053 p= 0.549
(O−E)/E: deviation usage of optimal codons
Codon position
L. Codon bias, Non−operonic v Operonic
(O−E)/E: Operonic
(O−E)/E: Non−operonic
Orthogonal regression slope = 1
Intercept = −0.0296
r = 0.253
P = 0.00181
