# Supplementary material for: Bacterial gene 5′ ends have unusual mutation rates that can mislead tests of selection
Source: PLoS Biol. 2025 Dec 15;23(12):e3003569. doi: 10.1371/journal.pbio.3003569 (PMC12725619; doi:10.1371/journal.pbio.3003569)
Supplement: S13 Fig — Codon optimality scores obtained from Wei and colleagues [74]. The data underlying this Figure can be found in https://doi.org/10.5281/zenodo.17378284. (PDF) [file pbio.3003569.s013.pdf]

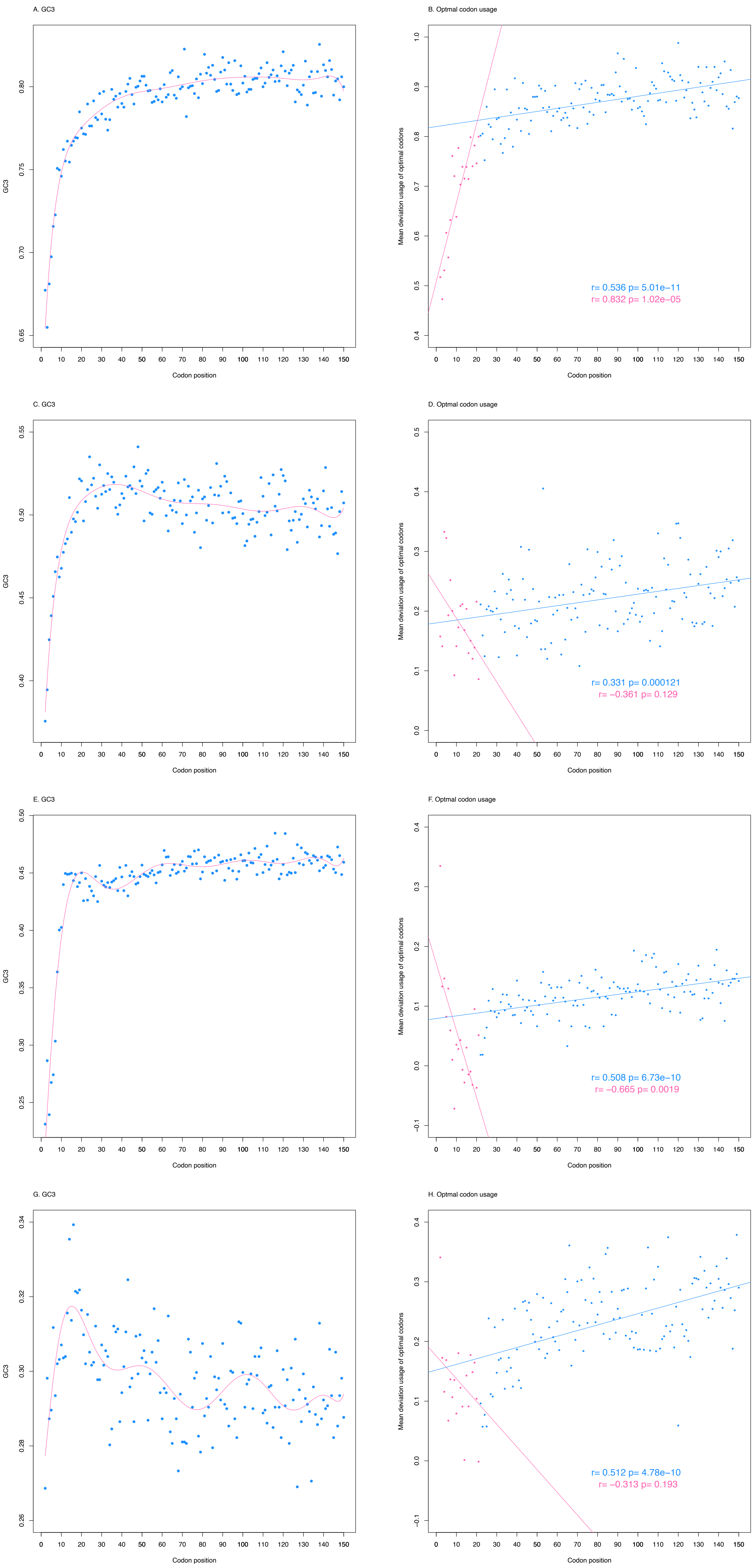
A. GC3
GC3
Codon position
B. Optmal codon usage
Mean deviation usage of optimal codons
Codon position
r= 0.536 p= 5.01e−11
r= 0.832 p= 1.02e−05
C. GC3
GC3
Codon position
D. Optmal codon usage
Mean deviation usage of optimal codons
Codon position
r= 0.331 p= 0.000121
r= −0.361 p= 0.129
E. GC3
GC3
Codon position
F. Optimal codon usage
Mean deviation usage of optimal codons
Codon position
r= 0.508 p= 6.73e−10
r= −0.665 p= 0.0019
G. GC3
GC3
Codon position
H. Optmal codon usage
Mean deviation usage of optimal codons
Codon position
r= 0.512 p= 4.78e−10
r= −0.313 p= 0.193
