# Supplementary material for: Bacterial gene 5′ ends have unusual mutation rates that can mislead tests of selection
Source: PLoS Biol. 2025 Dec 15;23(12):e3003569. doi: 10.1371/journal.pbio.3003569 (PMC12725619; doi:10.1371/journal.pbio.3003569)
Supplement: S14 Fig — Linear regression lines (and respective displayed Pearson correlation and P-value) in color consider the first 10 codons (inclusive), those in black are for all other codon positions. Plots with lines, statistics, and titles in pink show those amino acid blocks where the optimal codon is A/T-ending, those in blue have a G/C-ending optimal codon. Optimal codons and degeneracy for each block are indicated in the plot title. Note the 6-fold degenerate amino acids are divided into a 4-fold and a 2-fold block. The data underlying this Figure can be found in https://doi.org/10.5281/zenodo.17378284. (PDF) [file pbio.3003569.s014.pdf]

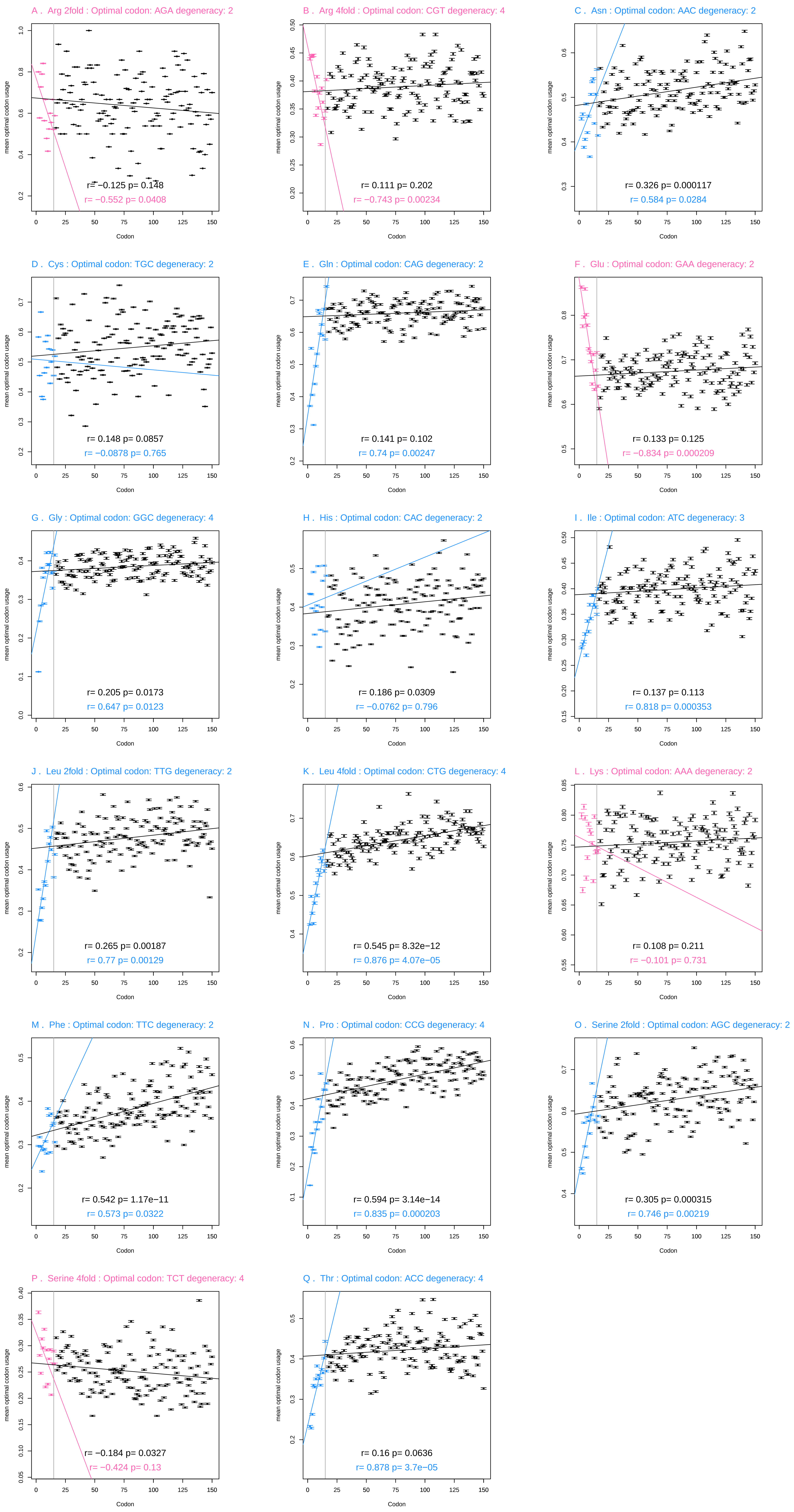

A . Arg 2fold : Optimal codon: AGA degeneracy: 2
r= −0.125 p= 0.148
r= −0.552 p= 0.0408
B . Arg 4fold : Optimal codon: CGT degeneracy: 4
r= 0.111 p= 0.202
r= −0.743 p= 0.00234
C . Asn : Optimal codon: AAC degeneracy: 2
r= 0.326 p= 0.000117
r= 0.584 p= 0.0284
D . Cys : Optimal codon: TGC degeneracy: 2
r= 0.148 p= 0.0857
r= −0.0878 p= 0.765
E . Gln : Optimal codon: CAG degeneracy: 2
r= 0.141 p= 0.102
r= 0.74 p= 0.00247
F . Glu : Optimal codon: GAA degeneracy: 2
r= 0.133 p= 0.125
r= −0.834 p= 0.000209
G . Gly : Optimal codon: GGC degeneracy: 4
r= 0.205 p= 0.0173
r= 0.647 p= 0.0123
H . His : Optimal codon: CAC degeneracy: 2
r= 0.186 p= 0.0309
r= −0.0762 p= 0.796
I . Ile : Optimal codon: ATC degeneracy: 3
r= 0.137 p= 0.113
r= 0.818 p= 0.000353
J . Leu 2fold : Optimal codon: TTG degeneracy: 2
r= 0.265 p= 0.00187
r= 0.77 p= 0.00129
K . Leu 4fold : Optimal codon: CTG degeneracy: 4
r= 0.545 p= 8.32e−12
r= 0.876 p= 4.07e−05
L . Lys : Optimal codon: AAA degeneracy: 2
r= 0.108 p= 0.211
r= −0.101 p= 0.731
M . Phe : Optimal codon: TTC degeneracy: 2
r= 0.542 p= 1.17e−11
r= 0.573 p= 0.0322
N . Pro : Optimal codon: CCG degeneracy: 4
r= 0.594 p= 3.14e−14
r= 0.835 p= 0.000203
O . Serine 2fold : Optimal codon: AGC degeneracy: 2
r= 0.305 p= 0.000315
r= 0.746 p= 0.00219
P . Serine 4fold : Optimal codon: TCT degeneracy: 4
r= −0.184 p= 0.0327
r= −0.424 p= 0.13
Q . Thr : Optimal codon: ACC degeneracy: 4
r= 0.16 p= 0.0636
r= 0.878 p= 3.7e−05
mean optimal codon usage
Codon
