# Supplementary material for: Bacterial gene 5′ ends have unusual mutation rates that can mislead tests of selection
Source: PLoS Biol. 2025 Dec 15;23(12):e3003569. doi: 10.1371/journal.pbio.3003569 (PMC12725619; doi:10.1371/journal.pbio.3003569)
Supplement: S15 Fig — Linear regression lines (and respective displayed Pearson correlation and P-value) in color consider the first 10 codons (inclusive), those in black are for all other codon positions. Plots with lines, statistics, and titles in pink show those amino acid blocks where the optimal codon is A/T-ending, those in blue have a G/C-ending optimal codon. Optimal codons and degeneracy for each block are indicated in the plot title. Note the 6-fold degenerate amino acids are divided into a 4-fold and a 2-fold block. The data underlying this Figure can be found in https://doi.org/10.5281/zenodo.17378284. (PDF) [file pbio.3003569.s015.pdf]

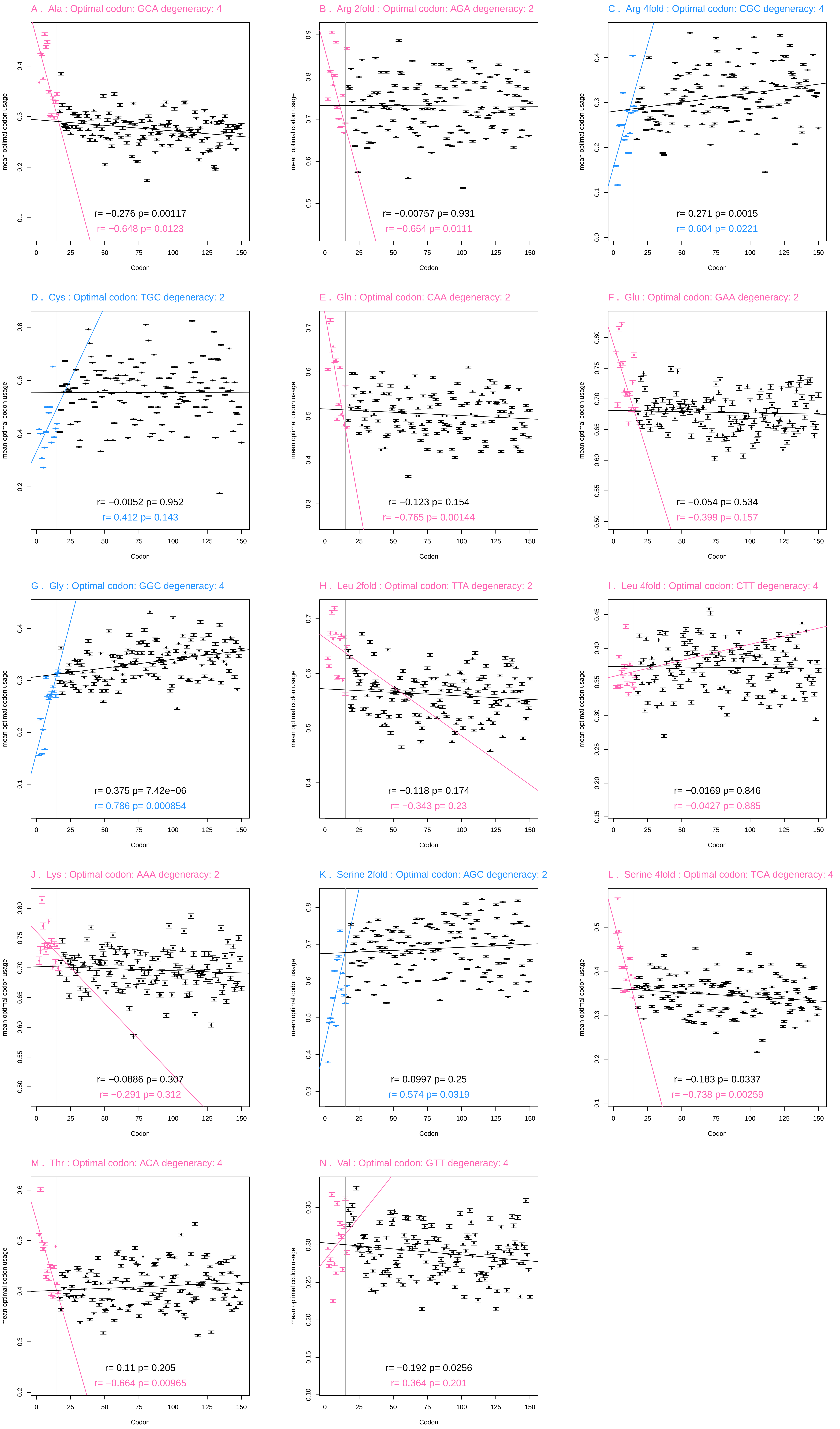

A . Ala : Optimal codon: GCA degeneracy: 4
r= −0.276 p= 0.00117
r= −0.648 p= 0.0123
B . Arg 2fold : Optimal codon: AGA degeneracy: 2
r= −0.00757 p= 0.931
r= −0.654 p= 0.0111
C . Arg 4fold : Optimal codon: CGC degeneracy: 4
r= 0.271 p= 0.0015
r= 0.604 p= 0.0221
D . Cys : Optimal codon: TGC degeneracy: 2
r= −0.0052 p= 0.952
r= 0.412 p= 0.143
E . Gln : Optimal codon: CAA degeneracy: 2
r= −0.123 p= 0.154
r= −0.765 p= 0.00144
F . Glu : Optimal codon: GAA degeneracy: 2
r= −0.054 p= 0.534
r= −0.399 p= 0.157
G . Gly : Optimal codon: GGC degeneracy: 4
r= 0.375 p= 7.42e−06
r= 0.786 p= 0.000854
H . Leu 2fold : Optimal codon: TTA degeneracy: 2
r= −0.118 p= 0.174
r= −0.343 p= 0.23
I . Leu 4fold : Optimal codon: CTT degeneracy: 4
r= −0.0169 p= 0.846
r= −0.0427 p= 0.885
J . Lys : Optimal codon: AAA degeneracy: 2
r= −0.0886 p= 0.307
r= −0.291 p= 0.312
K . Serine 2fold : Optimal codon: AGC degeneracy: 2
r= 0.0997 p= 0.25
r= 0.574 p= 0.0319
L . Serine 4fold : Optimal codon: TCA degeneracy: 4
r= −0.183 p= 0.0337
r= −0.738 p= 0.00259
M . Thr : Optimal codon: ACA degeneracy: 4
r= 0.11 p= 0.205
r= −0.664 p= 0.00965
N . Val : Optimal codon: GTT degeneracy: 4
r= −0.192 p= 0.0256
r= 0.364 p= 0.201
mean optimal codon usage
Codon
0 25 50 75 100 125 150
