# Supplementary material for: Bacterial gene 5′ ends have unusual mutation rates that can mislead tests of selection
Source: PLoS Biol. 2025 Dec 15;23(12):e3003569. doi: 10.1371/journal.pbio.3003569 (PMC12725619; doi:10.1371/journal.pbio.3003569)
Supplement: S1 Text — (PDF) [file pbio.3003569.s028.pdf]

Amino acid usage at the 5' ends of genes forces out GC rich runs

Having established that the 5' to 3' trend in synonymous substitution rates can be explained by four-fold site mutation rates (and biases) expected from genomic trinucleotide mutation rates (see Main Text Fig 5B, Fig 6D), a question that remains is what explains the observed distribution of mutability throughout genes. As we find that higher mutational rates are observed in GC-rich trinucleotides (Main Text Fig 5A, B), naturally, we may be seeing selection for high 5' A content to enable low RNA stability - the classical model. We however notice a peculiarity not so obviously consistent with this model: there is higher 5' A content in codon second sites than at four-fold degenerate sites at the 5' end (Main Text Fig 1). This is reflected when we consider native trends by codon position in trinucleotides that prevent GC richness ([A/T][N][A/T], where N is any of the four nucleotides occurring at codon third sites), for which we find high usage at 5' ends that decreases moving along the genes (S1 Text Fig 1). While these trends could be capturing what we see in the K_s trends in the most 5' codons, frequencies for trinucleotides stabilise around codon 10 (S1 Text Fig 1), suggesting that the K_s effect (stabilising around codon 60) is owing to a more complex relationship between trinucleotide frequencies and intra-gene position.

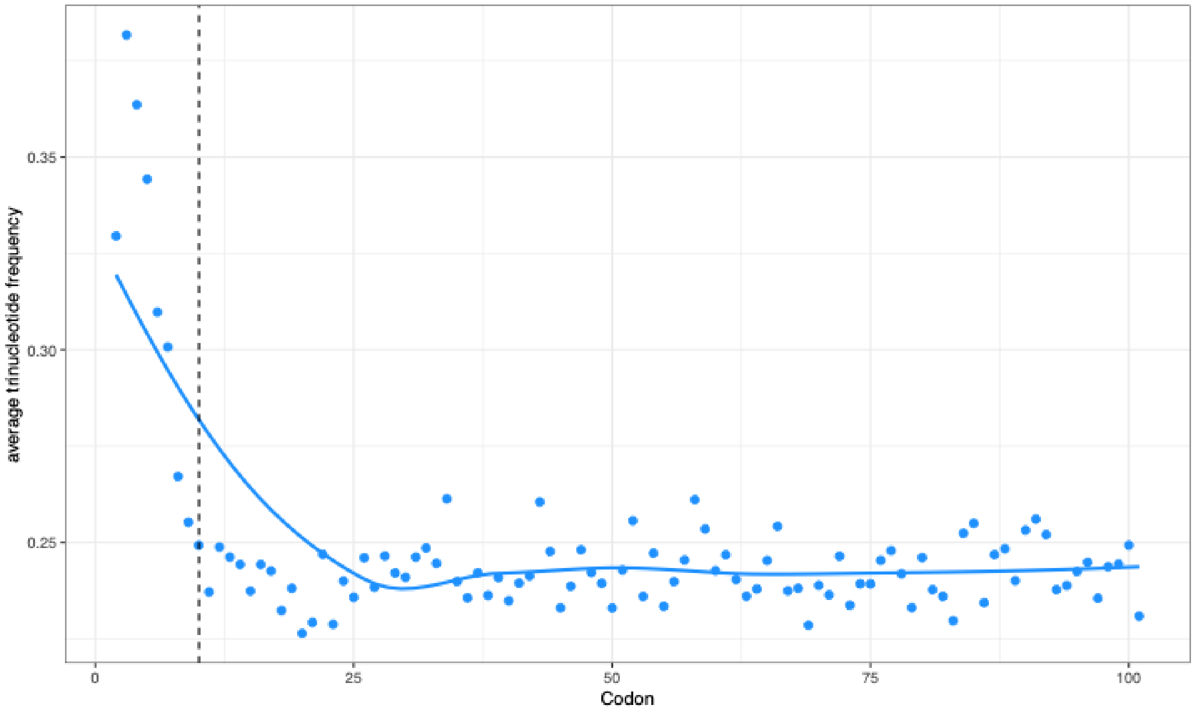

S1 Text Fig 1. Average AT-rich trinucleotide frequencies by codon position in native genes. Summed frequencies for ANT + ANA + TNT + TNA trinucleotides where N is any of the four nucleotides. Only trinucleotides where the middle base occurred at a codon third site were considered. Position on the x axis refers to codons, dashed vertical black line marks the first 10 codons, and locally estimated scatterplot smoothing (LOESS) regression line included.

From these results, it seems that the trend seen in K_s is congruent with trends in spontaneous mutation rates, this in turn primarily explained by GC-associated trinucleotide densities. But what makes GC runs so uncommon at 5' ends? The very high A richness at codon second site, higher than third sites, is not only not obviously expected from an RNA selection model, but also notable in two regards. First, codon second sites are most influential in dictating amino acid chemical properties [1], suggesting not simply selection for low RNA stability but also for proteins to encode particular classes of amino acids towards their ends. Second, with A enrichment at second sites, GC rich trinucleotides become more improbable. Preferences for certain amino acids, dictating in part nucleotide usage at codon sites 1 and 2, indeed have the potential to force out GC runs.

As high A content at second sites implies a peptide level effect, we can ask what properties of peptides may alter towards the N terminus. Jin et al find that codon site 2 is associated with 7 out of 13 chemical properties that they test [2]. These are polar requirement, aliphaticity, hydrogenation, chemical composition of the side chain, molecular volume, polarity and hydropathy index [2]. They also test, but don't associate to second sites, refractivity, aromaticity, hydroxythiolation, molecular weight, isoelectric point, and melting point [2]. We consider eight amongst these properties, four that they find associated to second sites (hydropathy, chemical composition of the side chain, molecular volume, polarity), and four that they don't (molecular weight, melting point, isoelectric point, refractivity). We find presence of A at second site to significantly negatively correlate with hydropathy and isoelectric point, and positively with molecular weight, polarity and sidechain composition (S1 Text Table 1).

S1 Text Table 1. Full and partial Spearman correlations between amino acid chemical properties and codon usage-position relation in *E. coli*. spearman_cor refers to the Spearman correlation retrieved by comparing codon usage and codon position for each of 61 codons (excluding stop codons); A_P2 refers to A content at codon second site only; hydropathy, scores determined according to the Kyte-Doolittle scale where low values represent hydrophilic amino acids (see Table2 in [3]); isoelectric: isoelectric point, defined as the pH at which the amino acid loses the electric charge. Scale retrieved from [4]; melt_point: melting point, scale retrieved from [4]; mol_volume: molecular volume, scale retrieved from [5]; mol_weight: molecular weight, scale retrieved from [4]; polarity, scale retrieved from [5]; refract: refractivity (i.e. the amount of refraction per gram of amino acid), scale retrieved from McMeekin et al [6] in Jones [7]; sidechain_comp: chemical composition of the sidechain (i.e. the atomic weight ratio of hetero (noncarbon) elements in end groups or rings to carbons in the side chain), scale retrieved from [5]. Number of start indicates significance such that p < .0001 = "****", p < .001 = "*** ", p < .01 = "** ", p < .05 = "* ", p > .05 = no stars

	**A_P2**	**hydropathy**	**isoelectric**	**melt_point**	**mol_volume**	**mol_weight**	**polarity**	**refract**	**sidechain_comp**	**spearman_cor**
**A_P2**		-0.5265****	-0.2863*	-0.2374	0.1666	0.4643***	0.5663****	0.2064	0.2641*	-0.1417
**hydropathy**	-0.097		-0.1283	0.6289****	-0.1118	-0.4365***	-0.8491****	-0.1229	-0.5398****	0.1208
**isoelectric**	-0.3862**	-0.6358****		0.1032	0.232	0.0596	-0.0622	0.1143	-0.4498***	-0.1214
**melt_point**	0.0912	0.3632**	0.0936		0.1264	-0.0974	-0.5623****	0.0382	-0.6525****	0.2029
**mol_volume**	-0.3421*	0.1295	0.1134	0.0389		0.8862****	-0.1865	0.9001****	-0.4296***	-0.4238***
**mol_weight**	0.169	-0.5816****	-0.5670****	0.1082	0.5892****		0.165	0.8828****	-0.1182	-0.3290**
**polarity**	0.3824**	-0.2651	0.3088*	0.0864	-0.0208	0.2776*		-0.1712	0.6687****	-0.0019
**refract**	0.1752	0.4465***	0.5486****	-0.1306	0.1743	0.6491****	-0.4526***		-0.2382	-0.2878*
**sidechain_comp**	-0.3751**	-0.4285**	-0.7218****	-0.1735	-0.2514	-0.3680**	0.4727***	0.6281****		0.0707

Considering usage in amino acid chemical properties as we move towards the N terminus in native *E. coli* peptides, we observe trends for the eight properties. For instance, we find 5' ends tend to contain hydrophilic amino acids compared to downstream (S1 Text Fig 2). The enrichment of hydrophilic amino acids was previously observed in *E. coli* and *B. subtilis*, where it was reported to be a commonality of both N and C termini and for it to extend along 10 codons [8]. Jin et al however report a tendency for hydrophobic amino acids at 5' ends instead [2].

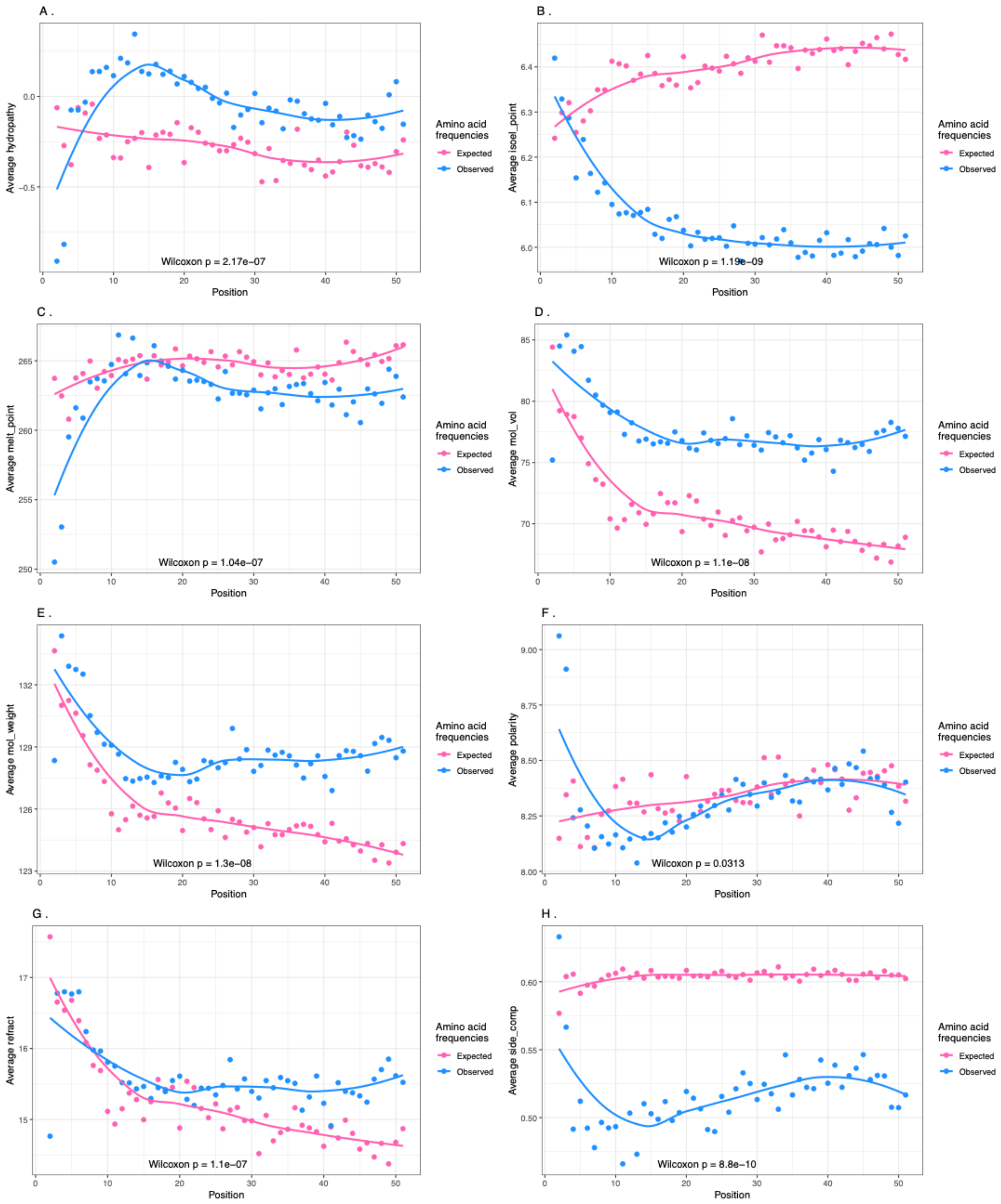

S1 Text Fig 2: Average observed and expected chemical property values by codon position in native *E. coli* genes. Values are found and colour-coded for observed codon frequencies (blue) or those expected from frequency of mononucleotides at third codon site of four-fold degenerate amino acids (pink). A range of chemical properties are considered: **A.** hydropathy, scores determined according to the Kyte-Doolittle scale where low values represent hydrophilic amino acids (see Table2 in [3]). **B.** Isoelectric point, defined as the pH at which the amino acid loses the electric charge. Scale retrieved from [4]. **C.** Melting point, scale retrieved from [4]. **D.** Molecular volume, scale retrieved from [5]. **E.** Molecular weight, scale retrieved from [4]. **F.** polarity, scale retrieved from [5]. **G.** refractivity (i.e. the amount of refraction per gram of amino acid), scale retrieved from McMeekin et al [6] in Jones [7]. **H.** Chemical composition of the sidechain (i.e. the atomic weight ratio of hetero (noncarbon) elements in end groups or rings to carbons in the side chain), scale retrieved from [5]. For all panels the x axis represents absolute codon position (i.e., the start codon is codon 1).

As a simple test of deviation from null, we additionally calculate expected frequencies by calculating probabilities for each codon based on the frequencies of mononucleotides found at the codon third site of four-fold degenerate amino acids at each codon position. Although four-fold mononucleotide trends don't match the A richness of codon second sites, they still show high AT content (especially A) within the most 5' codons, which then decreases until plateauing around codon 10 (Main Text Fig 1B). We therefore ask what the by-position trends of physiochemical properties would look like if codon frequencies followed the AT richness observed at four-fold synonymous sites. We see that the observed trends for hydropathy, chemical composition of the side chain, polarity, and melting point are different to what would be expected by the four-fold synonymous mononucleotide frequencies (S1 Text Fig 2). The difference is not as clear for molecular volume, molecular weight, and refractivity (S1 Text Fig 2). Results are qualitatively the same in *Bacillus* (S1 Text Fig 3), with the exception that higher, not lower, isoelectric point amino acids are preferred near the 5' end.

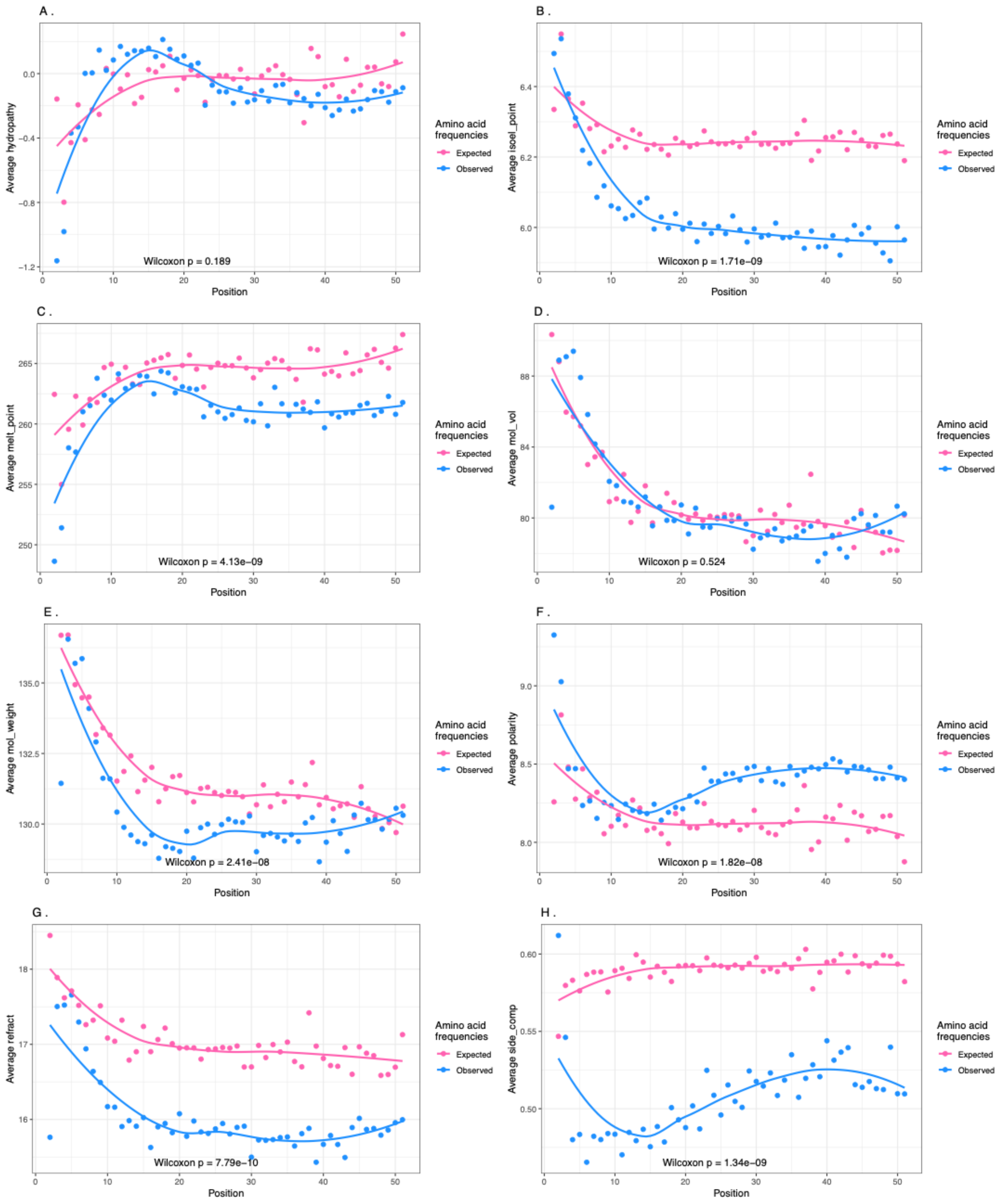

S1 Text Fig 3: Average observed and expected chemical property values by codon position in native *B. subtilis* genes. Values are found and colour-coded for observed codon frequencies (blue) or those expected from frequency of mononucleotides at third codon site of four-fold degenerate amino acids (pink). A range of chemical properties are considered: **A.** hydropathy, scores determined according to the Kyte-Doolittle scale where low values represent hydrophilic amino acids (see Table2 in [3]). **B.** Isoelectric point, defined as the pH at which the amino acid loses the electric charge. Scale retrieved from [4]. **C.** Melting point, scale retrieved from [4]. **D.** Molecular volume, scale retrieved from [5]. **E.** Molecular weight, scale retrieved from [4]. **F.** polarity, scale retrieved from [5]. **G.** refractivity (i.e. the amount of refraction per gram of amino acid), scale retrieved from McMeekin et al [6] in Jones [7]. **H.** Chemical composition of the sidechain (i.e. the atomic weight ratio of hetero (noncarbon) elements in end groups or rings to carbons in the side chain), scale retrieved from [5]. For all panels the x axis represents absolute codon position (i.e., the start codon is codon 1).

We then ask whether the underlying trends in chemical properties can actually explain the relationship between codon usage and codon position. First, we calculate the Spearman correlation between the usage frequency of each codon (relative to other codons) and its position, and we take this to represent the usage-position trend (a negative value indicating enrichment towards 5' end). Next, we assess both full and partial Spearman correlations between the usage-position trends and chemical property scores assigned to each codon, as well as A content at codon second sites. Our analysis revealed that molecular volume, molecular weight, and refractivity are significantly negatively correlated with usage-position slopes, whilst the remaining chemical properties and A content at codon second sites are not (S3 Table). This suggests in the first instance that the bigger predictor of the relation between codon usage and codon position is the codon's chemical property rather than its nucleotide content. Interestingly, A content at second sites has a significant negative correlation to the codon-usage slope when performing a partial correlation analysis controlling for all other covariates (S3 Table). Among the chemical properties, molecular volume retained a significant negative correlation in the partial correlation analysis (S3 Table). This suggests that higher molecular volume and higher A usage at codon second site are associated with a negative usage-position trend and hence are enriched at 5' ends. This effect is not replicated in *Bacillus* (S1 Text Table 2). Although in *E.coli* we find no other chemical property to remain significantly correlated in the partial correlation analysis, we note that substantial collinearity among all chemical properties could be masking effects.

S1 Text Table 2. Full and partial Spearman correlations between amino acid chemical properties and codon usage-position relation in *B. subtilis*. spearman_cor refers to the Spearman correlation retrieved by comparing codon usage and codon position for each of 61 codons (excluding stop codons); A_P2 refers to A content at codon second site only; hydropathy, scores determined according to the Kyte-Doolittle scale where low values represent hydrophilic amino acids (see Table2 in [3]); isoelectric: isoelectric point, defined as the pH at which the amino acid loses the electric charge. Scale retrieved from [4]; melt_point: melting point, scale retrieved from [4]; mol_volume: molecular volume, scale retrieved from [5]; mol_weight: molecular weight, scale retrieved from [4]; polarity, scale retrieved from [5]; refract: refractivity (i.e. the amount of refraction per gram of amino acid), scale retrieved from McMeekin et al [6] in Jones [7]; sidechain_comp: chemical composition of the sidechain (i.e. the atomic weight ratio of hetero (noncarbon) elements in end groups or rings to carbons in the side chain), scale retrieved from [5]. Number of start indicates significance such that p < .0001 = "****", p < .001 = "*** ", p < .01 = "** ", p < .05 = "* ", p > .05 = no stars

	A_P2	hydropathy	isoelectric	melt_point	mol_volume	mol_weight	polarity	refract	sidechain_comp	spearman_cor
**A_P2**		-0.5265****	-0.2863*	-0.2374	0.1666	0.4643***	0.5663****	0.2064	0.2641*	-0.2967*
**hydropathy**	-0.0979		-0.1283	0.6289****	-0.1118	-0.4365***	-0.8491****	-0.1229	-0.5398****	0.2761*
**isoelectric**	-0.3798**	-0.6425****		0.1032	0.232	0.0596	-0.0622	0.1143	-0.4498***	-0.1333
**melt_point**	0.045	0.3699**	0.0826		0.1264	-0.0974	-0.5623****	0.0382	-0.6525****	0.1879
**mol_volume**	-0.2850*	0.1496	0.1579	-0.0569		0.8862****	-0.1865	0.9001****	-0.4296***	-0.5161****
**mol_weight**	0.1424	-0.5963****	-0.5886****	0.145	0.5899****		0.165	0.8828****	-0.1182	-0.5302****
**polarity**	0.3447*	-0.2971*	0.2707*	0.1399	-0.1	0.2776*		-0.1712	0.6687****	-0.1803
**refract**	0.1328	0.4462***	0.5424****	-0.084	0.0962	0.6942****	-0.4221**		-0.2382	-0.4431***
**sidechain_comp**	-0.3508*	-0.4301**	-0.7197****	-0.2212	-0.2004	-0.3994**	0.4409***	0.6132****		0.0293

These tests suggests that the observed trends in GC-rich trinucleotides, which are likely partially linked to frequencies of A at codon second sites, are at least in part the result of chemical properties required at 5' ends - and therefore selection at the amino acid level. We suggest that, when considering 5' end biology, to understand synonymous rates of evolution more generally it is necessary to also consider amino acid usage as this will impose limits on rates of *k*-mers, *k*-mers that appear to be decisive in determining mutation rates. Note that second site A enrichment cannot alone account for what is happening at four-fold degenerate sites as in the genetic code there are no four-fold degenerate amino acids encoded by codons with an A at their second site.

References

1. Saier MH. Understanding the genetic code. Journal of Bacteriology. 2019;201(15). doi: 10.1128/jb.00091-19. PubMed PMID: WOS:000474755100002.
2. Jin Y-T, Jin T-Y, Zhang Z-L, Ye Y-N, Deng Z, Wang J, et al. Quantitative elucidation of associations between nucleotide identity and physicochemical properties of amino acids and the functional insight. Computational and Structural Biotechnology Journal. 2021;19:4042-8. doi: 10.1016/j.csbj.2021.07.0122001-0370/. PubMed PMID: WOS:000684852400007.
3. Kyte J, Doolittle RF. A simple method for displaying the hydropathic character of a protein. Journal of Molecular Biology. 1982;157(1):105-32. doi: 10.1016/0022-2836(82)90515-0. PubMed PMID: WOS:A1982NQ28000006.
4. Haynes WM. CRC handbook of chemistry and physics. 95 ed: CRC Press; 2014.
5. Grantham R. Amino acid difference formula to help explain protein evolution. Science. 1974;185(4154):862-4. doi: 10.1126/science.185.4154.862. PubMed PMID: WOS:A1974T914800023.
6. McMeekin TL, Groves ML, Hipp NJ. Refractive indices of amino acids, proteins, and related substances. Advances in chemistry. 441964. p. 54-66.
7. Jones DD. Amino acid properties and side-chain orientation in proteins: a cross-correlation approach. Journal of Theoretical Biology. 1975;50(1):167-83. doi: 10.1016/0022-5193(75)90031-4. PubMed PMID: WOS:A1975W005700013.
8. Rocha EPC, Danchin A, Viari A. Translation in Bacillus subtilis: roles and trends of initiation and termination, insights from a genome analysis. Nucleic Acids Research. 1999;27(17):3567-76. doi: 10.1093/nar/27.17.3567. PubMed PMID: WOS:000082507200024.
