# Supplementary material for: Bacterial gene 5′ ends have unusual mutation rates that can mislead tests of selection
Source: PLoS Biol. 2025 Dec 15;23(12):e3003569. doi: 10.1371/journal.pbio.3003569 (PMC12725619; doi:10.1371/journal.pbio.3003569)
Supplement: S2 Text — (PDF) [file pbio.3003569.s029.pdf]

Why are mutational equilibrium estimates so diverse?

In addition to analysis of substitution rate trends, we have also tested for selection by consideration of deviation for mutational neutral equilibria. This proved quite singularly unhelpful. Analysis using *E. coli* MA lines [1, 2] predicted the AT content at mutational equilibrium (AT*) to be in the range 0.55-0.65 (see [3] and Main Text Table 1), while the spontaneous mutational data with Duplex Sequencing of Zhang et al. [4] finds it to be considerably higher (approximately 0.85, Main Text Fig 7, Main Text Table 1, Main Text S3 Table). Why might the MA estimates and the high-resolution mutational analysis differ so much?

There is no robust evidence for strain-specific differences [1] so we probably need to search elsewhere for an answer. As we showed (Main Text S3 Table), there is also no evidence that using different methods to calculate the equilibrium has any major effect. What perhaps is striking is that the two highest estimates are both derived via Duplex Sequencing, while MA line data is closer to 0.6 (Main Text Table 1).

There are likely to be differences between experimental methods in the estimate of absolute rates with some alternative methodologies (e.g., fluctuation tests) underestimating, potentially owing to negative selection, phenotypic lag or imperfect plating [5]. Moreover, for any depth of sequencing, there will be a minimum frequency that a mutation must achieve to be confidently called - the lower the depth of sequencing, the fewer the mutations that will be seen because they are under negative selection or yet to drift to higher frequencies. An alternative possibility, however, is that higher depth sequencing captures more mutations, but these are then in the short term repaired and so not mutations in an evolutionary genetical sense (they could never accumulate in populations over time). There is some evidence for this [5]. Absence of different repair mechanisms will also skew mutational bias [6]. An absence of mutY MMR repair leads to a profound A/T->G/C bias [1, 7-9], while oxidative stress associated mutations unrepaired by mutT activity have an A/T->G/C bias [1]. Performing our test on data from a further Duplex Sequencing-based study [6] reports an AT*~0.89 in WT *E. coli* (Main Text Table 1), this being higher than seen in MMR-defective *E. coli* (mutL knockouts AT*=0.60) generated in the same study, and importantly comparable to that of Zhang et al. [4] (also Duplex Sequencing-based).

Several lines of evidence argue against a bias owing to yet-to-be-performed repair. First, when we consider the samples sequenced at low depth using a dinucleotide model, which should capture only more common mutations, we still recover genomic AT*~0.89 (Main Text S3 Table). The

only peculiarity is that when we consider a mononucleotide model applied to the low resolution data set, the genomic AT* now resolves to ~0.67, still higher than that observed in native genes but less so (Main Text Table 1, Main Text S3 Table, Main Text Fig 7). Second, to test for the possibility of over-calling mutations we can restrict analysis to those mutations most confidently called (higher mutation calling significance i.e., lower P-value). Restricting analysis to the quartile of mutations that have the lowest P-value (least likely to be mis-called), the equilibrium AT frequency remains high (using a simple mononucleotide method to compare directly with Long et al.'s method, for lower depth mutation data AT*=0.75, for higher depth mutation data AT*=0.77, S2 Text Table 1). In the higher depth mutation data, the genomic AT* for the three less high quality quartiles is ~0.89 (S2 Text Table 1). In the samples sequenced with lower depth, analysis of the quartile of the least confidently called mutations resolves to AT*=0.51, suggesting that, if anything, analysis of the full low coverage set is conservative.

S2 Text Table 1. Estimated AT content at mutational equilibrium determined by simple method and divided by mutation calling significance group. Method involves calculation of proportion of G/C<->A/T mutations that are G/C->A/T directed, see Methods. "seq_depth" differentiates the samples into those sequenced with higher or lower depth by Zhang et al. "mut_size" refers to the approach by which the mutational matrix was generated i.e., counting mononucleotide mutations. "pval_quartile" refers to mutation grouping based on mutation calling significance reported by Zhang et al. (Q1 refers to the group with the lowest P-value i.e., highest significance, all refers to all mutations from all four quartiles). "region" refers to 5' ends (i.e., the first 20 codons following the start codon), gene cores (i.e., the rest of the CDS), intergenic (i.e., non-CDS mutations), or genomic (i.e., the whole genome including non-protein coding sequences). "pred_GC" and "pred_AT" are respectively the predicted GC and AT content at mutational equilibrium.

seq_depth	mut_size	pval_quartile	region	pred_GC	pred_AT
high	mononucs	Q1	genomic	0.22842113	0.77157887
high	mononucs	Q1	5' end	0.1434073	0.8565927
high	mononucs	Q1	intergenic	0.28326393	0.71673607
high	mononucs	Q1	core	0.20084942	0.79915058
high	mononucs	Q2	genomic	0.10671351	0.89328649
high	mononucs	Q2	5' end	0.07236644	0.92763356
high	mononucs	Q2	intergenic	0.11419841	0.88580159
high	mononucs	Q2	core	0.10608443	0.89391557
high	mononucs	Q3	genomic	0.11469286	0.88530714
high	mononucs	Q3	5' end	0.0611265	0.9388735
high	mononucs	Q3	intergenic	0.10943283	0.89056717
high	mononucs	Q3	core	0.11809811	0.88190189
high	mononucs	Q4	genomic	0.11307114	0.88692886
high	mononucs	Q4	5' end	0.08823017	0.91176983
high	mononucs	Q4	intergenic	0.11245277	0.88754723
high	mononucs	Q4	core	0.11098104	0.88901896
high	mononucs	all	genomic	0.14054357	0.85945643
high	mononucs	all	5' end	0.08857237	0.91142763
high	mononucs	all	intergenic	0.34103277	0.65896723

high	mononucs	all	core	0.13197731	0.86802269
low	mononucs	Q1	genomic	0.2540355	0.7459645
low	mononucs	Q1	5' end	0.14438371	0.85561629
low	mononucs	Q1	intergenic	0.32241132	0.67758868
low	mononucs	Q1	core	0.20462337	0.79537663
low	mononucs	Q2	genomic	0.21631202	0.78368798
low	mononucs	Q2	5' end	0.15986797	0.84013203
low	mononucs	Q2	intergenic	0.19559656	0.80440344
low	mononucs	Q2	core	0.2199589	0.7800411
low	mononucs	Q3	genomic	0.37971233	0.62028767
low	mononucs	Q3	5' end	0.27027095	0.72972905
low	mononucs	Q3	intergenic	0.25516174	0.74483826
low	mononucs	Q3	core	0.41788223	0.58211777
low	mononucs	Q4	genomic	0.49380268	0.50619732
low	mononucs	Q4	5' end	0.39845806	0.60154194
low	mononucs	Q4	intergenic	0.36908079	0.63091921
low	mononucs	Q4	core	0.52847711	0.47152289
low	mononucs	all	genomic	0.33493128	0.66506872
low	mononucs	all	5' end	0.24634519	0.75365481
low	mononucs	all	intergenic	0.34904693	0.65095307
low	mononucs	all	core	0.34440398	0.65559602

Third, as repair is directed to the genes [1, 7], we can also ask whether the mutation rate and mutational properties differ between intergenic sequence and annotated genes. By determining trinucleotide mutation counts per occurrence of that trinucleotide in genic and intergenic regions separately, we find that in Zhang et al.'s Duplex Sequencing data [4] intergenic sequences have over one and a half the number of mutations as the same trinucleotides when they are genic (mean normalised mutation rate intergenic/mean normalised mutation rate genic: for high depth samples=1.56 +/- 0.39 (sd), paired t test with null of no difference, P-value=2.2 x 10^{-16}, df=63; for low depth samples=1.64 +/- 0.41 (sd), P-value=2.5 x 10^{-14}, df=63). This is similar to the ratio previously suggested to be expected owing to CDS-centred repair [1] when converted to a metric of mutations per bp comparable to ours (ratio intergenic/genic=1.76). In both the high and low depth samples, there is thus evidence that repair has acted differentially on genes compared to intergenic sequence, suggesting that we are unlikely to be capturing false mutations pre-repair. This assumes that the mutability associated with each trinucleotide are similar in non-coding and coding sequences. The equilibrium AT content using only mutational data from intergenic regions we find to be a little lower than genic regions, though not as low as that reported from MA lines (for higher depth samples, intergenic AT*=0.83, and for lower depth, intergenic AT*=0.73, Main Text S3

Table). As then expected, the mutation rate per occurrence of each trinucleotide in intergenic sequence and CDS is highly correlated (for Zhang et al. higher depth samples Pearson correlation r=0.92, P-value=5 x 10^{-27}; lower depth samples: r=0.92, P-value=1 x 10^{-26}; Main Text S18 Fig) this underscoring the centrality of trinucleotide content as the determinant of relative mutation rates. We caution however, that in Wei et al.'s MA data [2], while the intergenic and genic mutations rates are correlated, the correlation is nothing like as strong (for WT Pearson correlation r=0.39, P-value=0.002, Main Text S18 Fig), this potentially reflecting low mutational numbers in rare intergenic sequence, with some trinucleotides having no reported mutation.

One further possibility remains, however, this being that sample preparation affects the DNA and mutations called. DNA damage in library preparation is a possibility, as is oxidative and heat induced damage during ultrasonication [10, 11]. Duplex Sequencing was noted to output a high background mutation frequency, potentially as a consequence of this [6] with an A/T->G/C bias. Zhang et al. [4], alive to such issues, score their mutations against a benchmark of control mutations that have undergone the same sample preparation. That we find the strong A/T->G/C bias in their most extremely likely mutations argues against sample preparation issues. Nonetheless, we can't be sure that sample preparation associated with Duplex Sequencing hasn't affected the mutation biases, especially considering the only other AT* estimate that is as comparably high to that of Zhang et al. [4] was derived from another Duplex Sequencing-based study [6], in contrast to MA experiments (Main Text Table 1). When the best directly determined mutation data and other MA line data disagree so profoundly, close examination of possible biases in all data sets is warranted.

References

1. Foster PL, Lee H, Popodi E, Townes JP, Tang H. Determinants of spontaneous mutation in the bacterium Escherichia coli as revealed by whole-genome sequencing. Proceedings of the National Academy of Sciences of the United States of America. 2015;112(44):E5990-E9. doi: 10.1073/pnas.1512136112. PubMed PMID: WOS:000364164900014.

2. Wei W, Ho W-C, Behringer MG, Miller SF, Bcharah G, Lynch M. Rapid evolution of mutation rate and spectrum in response to environmental and population-genetic challenges. Nature Communications. 2022;13(1). doi: 10.1038/s41467-022-32353-6. PubMed PMID: WOS:000840338100006.

3. Long HA, Sung W, Kucukyildirim S, Williams E, Miller SF, Guo WF, et al. Evolutionary determinants of genome-wide nucleotide composition. Nature Ecology & Evolution. 2018;2(2):237-+. doi: 10.1038/s41559-017-0425-y. PubMed PMID: WOS:000426516400012.

4. Zhang X, Zhang X, Zhang X, Liao Y, Song L, Zhang Q, et al. Spatial vulnerabilities of the Escherichia coli genome to spontaneous mutations revealed with improved duplex sequencing. Genetics. 2018;210(2):547-58. doi: 10.1534/genetics.118.301345. PubMed PMID: WOS:000449398800012.

5. Jee J, Rasouly A, Shamovsky I, Akivis Y, Steinman SR, Mishra B, et al. Rates and mechanisms of bacterial mutagenesis from maximum-depth sequencing. Nature. 2016;534(7609):693-+. doi: 10.1038/nature18313. PubMed PMID: WOS:000378676000038.
6. Bhawsinghka N, Burkholder A, Schaaper RM. Detection of DNA replication errors and 8-oxo-dGTP-mediated mutations in E. coli by Duplex DNA Sequencing. DNA Repair. 2023;123. doi: 10.1016/j.dnarep.2023.103462. PubMed PMID: WOS:000930995700001.
7. Lee H, Popodi E, Tang H, Foster PL. Rate and molecular spectrum of spontaneous mutations in the bacterium Escherichia coli as determined by whole-genome sequencing. Proceedings of the National Academy of Sciences of the United States of America. 2012;109(41):E2774-E83. doi: 10.1073/pnas.1210309109. PubMed PMID: WOS:000310280300007.
8. Yanofsky C, Cox EC, Horn V. The unusual mutagenic specificity of an Escherichia coli mutator gene. Proc Nat Acad Sci USA. 1966;55((2)):274-81. doi: 10.1073/pnas.55.2.274. PubMed PMID: BCI:BCI19674800003380.
9. Fowler RG, Schaaper RM. The role of the mutT gene of Escherichia coli in maintaining replication fidelity. Fems Microbiology Reviews. 1997;21(1):43-54. doi: 10.1016/s0168-6445(97)00045-4. PubMed PMID: WOS:A1997XV13800003.
10. Arbeithuber B, Makova KD, Tiemann-Boege I. Artifactual mutations resulting from DNA lesions limit detection levels in ultrasensitive sequencing applications. DNA Research. 2016;23(6):547-59. doi: 10.1093/dnares/dsw038. PubMed PMID: WOS:000392723300005.
11. Costello M, Pugh TJ, Fennell TJ, Stewart C, Lichtenstein L, Meldrim JC, et al. Discovery and characterization of artifactual mutations in deep coverage targeted capture sequencing data due to oxidative DNA damage during sample preparation. Nucleic Acids Research. 2013;41(6). doi: 10.1093/nar/gks1443. PubMed PMID: WOS:000318063400002.
